# A novel method for inference of acyclic chemical compounds with bounded branch-height based on artificial neural networks and integer programming

**DOI:** 10.1186/s13015-021-00197-2

**Published:** 2021-08-14

**Authors:** Naveed Ahmed Azam, Jianshen Zhu, Yanming Sun, Yu Shi, Aleksandar Shurbevski, Liang Zhao, Hiroshi Nagamochi, Tatsuya Akutsu

**Affiliations:** 1 Department of Applied Mathematics and Physics, Kyoto University, Yoshida Honmachi, Sakyo, Kyoto, 606-8501 Japan; 2 Graduate School of Advanced Integrated Studies in Human Survivability, Kyoto University, Yoshida Nakaadachi-cho, Sakyo, Kyoto, 606-8306 Japan; 3 Bioinformatics Center, Institute for Chemical Research, Kyoto University, Gokasho, Uji, 611-0011 Japan

**Keywords:** QSAR/QSPR, Molecular design, Artificial neural network, Mixed integer linear programming, Enumeration of graphs, Primary, 05C92, 92E10, Secondary, 05C30, 68T07, 90C11, 92-04

## Abstract

Analysis of chemical graphs is becoming a major research topic in computational molecular biology due to its potential applications to drug design. One of the major approaches in such a study is inverse quantitative structure activity/property relationship (inverse QSAR/QSPR) analysis, which is to infer chemical structures from given chemical activities/properties. Recently, a novel two-phase framework has been proposed for inverse QSAR/QSPR, where in the first phase an artificial neural network (ANN) is used to construct a prediction function. In the second phase, a mixed integer linear program (MILP) formulated on the trained ANN and a graph search algorithm are used to infer desired chemical structures. The framework has been applied to the case of chemical compounds with cycle index up to 2 so far. The computational results conducted on instances with *n* non-hydrogen atoms show that a feature vector can be inferred by solving an MILP for up to $n=40$, whereas graphs can be enumerated for up to $n=15$. When applied to the case of chemical acyclic graphs, the maximum computable diameter of a chemical structure was up to 8. In this paper, we introduce a new characterization of graph structure, called “branch-height” based on which a new MILP formulation and a new graph search algorithm are designed for chemical acyclic graphs. The results of computational experiments using such chemical properties as octanol/water partition coefficient, boiling point and heat of combustion suggest that the proposed method can infer chemical acyclic graphs with around $n=50$ and diameter 30.

## Background

In computational molecular biology, various types of data have been utilized, which include sequences, gene expression patterns, and protein structures. Graph structured data have also been extensively utilized, which include metabolic pathways, protein-protein interaction networks, gene regulatory networks, and chemical graphs. Much attention has recently been paid to the analysis of chemical graphs due to its potential applications to computer-aided drug design. One of the major approaches to computer-aided drug design is quantitative structure activity/property relationship (QSAR/QSPR) analysis, the purpose of which is to derive quantitative relationships between chemical structures and their activities/properties. Furthermore, inverse QSAR/QSPR has been extensively studied [1, 2], the purpose of which is to infer chemical structures from given chemical activities/properties. Inverse QSAR/QSPR is often formulated as an optimization problem to find a chemical structure maximizing (or minimizing) an objective function under various constraints.

In both QSAR/QSPR and inverse QSAR/QSPR, chemical compounds are usually represented as vectors of real or integer numbers, which are often called *descriptors* and correspond to *feature vectors* in machine learning. Using these chemical descriptors, various heuristic and statistical methods have been developed for finding optimal or nearly optimal graph structures under given objective functions [1, 3, 4]. Inference or enumeration of graph structures from a given feature vector is a crucial subtask in many of such methods. Various methods have been developed for this enumeration problem [5–8] and the computational complexity of the inference problem has been analyzed [9, 10]. On the other hand, enumeration in itself is a challenging task, since the number of molecules (i.e., chemical graphs) with up to 30 atoms (vertices) C, N, O, and S, may exceed $10^{60}$ [11].

As a new approach, artificial neural network (ANN) and deep learning technologies have recently been applied to inverse QSAR/QSPR. For example, variational autoencoders [12], recurrent neural networks [13, 14], and grammar variational autoencoders [15] have been applied. In these approaches, new chemical graphs are generated by solving a kind of inverse problems on neural networks that are trained using known chemical compound/activity pairs. However, the optimality of the solution is not necessarily guaranteed in these approaches. In order to guarantee the optimality mathematically, a novel approach has been proposed [16] for ANNs, using mixed integer linear programming (MILP).

Recently, a new framework has been proposed [17–19] by combining two previous approaches: efficient enumeration of tree-like graphs [5], and MILP-based formulation of the inverse problem on ANNs [16]. This combined framework for inverse QSAR/QSPR mainly consists of two phases. The first phase solves (I) Prediction Problem, where a feature vector *f*(*G*) of a chemical graph *G* is introduced and a prediction function $\psi _{{{\mathcal {N}}}}$ on a chemical property $\pi$ is constructed with an ANN ${{\mathcal {N}}}$ using a data set of chemical compounds *G* and their values *a*(*G*) of $\pi$. The second phase solves (II) Inverse Problem, where (II-a) given a target value $y^*$ of the chemical property $\pi$, a feature vector $x^*$ is inferred from the trained ANN ${{\mathcal {N}}}$ so that $\psi _{{{\mathcal {N}}}}(x^*)$ is close to $y^*$ and (II-b) then a set of chemical structures $G^*$ such that $f(G^*)= x^*$ is enumerated by a graph search algorithm. In (II-a) of the above-mentioned previous methods [17–19], an MILP is formulated for acyclic chemical compounds. Afterwards, Ito et al. [20] and Zhu et al. [21] designed a method of inferring chemical graphs with cycle index 1 and 2, respectively, by formulating a new MILP and using an efficient algorithm for enumerating chemical graphs with cycle index 1 [22] and cycle index 2 [23, 24]. The computational results conducted on instances with *n* non-hydrogen atoms show that a feature vector $x^*$ can be inferred for up to around $n=40$ whereas graphs $G^*$ can be enumerated for up to around $n=15$.

In this paper, we present a new characterization of graph structure, called “branch-height.” Based on this, we can treat a class of acyclic chemical graphs with a structure that is topologically restricted but frequently appears in a chemical database, formulate a new MILP formulation that can handle acyclic graphs with a large diameter, and design a new graph search algorithm that generates acyclic chemical graphs with up to around 50 vertices. The results of computational experiments using such chemical properties as octanol/water partition coefficient, boiling point and heat of combustion suggest that the proposed method is much more useful than the previous method.

The paper is organized as follows. "Preliminary" section introduces some notions on graphs, a modeling of chemical compounds and a choice of descriptors. "A method for inferring chemical graphs" section reviews the framework for inferring chemical compounds based on ANNs and MILPs. "MILPs for chemical acyclic graphs with bounded branch-height" section introduces a new method of modeling acyclic chemical graphs and proposes a new MILP formulation that represents an acyclic chemical graph *G* with *n* vertices, where our MILP requires only *O*(*n*) variables and constraints when the branch-parameter *k* and the *k*-branch height in *G* (graph topological parameters newly introduced in this paper) is constant. "A new graph search algorithm" section describes the idea of our new dynamic programming type of algorithm that enumerates a given number of acyclic chemical graphs for a given feature vector. "Experimental results" section reports the results on some computational experiments conducted for chemical properties such as octanol/water partition coefficient, boiling point and heat of combustion. "Concluding remarks" section makes some concluding remarks. Appendix A provides the statistical distribution of structural features of acyclic chemical graphs in a chemical graph database. Appendices B and C describe the idea of our MILP formulation and the details of all variables and constraints in the MILP formulation, respectively. Appendix D presents descriptions of our new graph search algorithm.

## Preliminary

This section introduces some notions and terminology on graphs, a modeling of chemical compounds and our choice of descriptors.

Let ${\mathbb {R}}$, ${\mathbb {Z}}$ and ${\mathbb {Z}}_+$ denote the sets of reals, integers and non-negative integers, respectively. For two integers *a* and *b*, let [*a*, *b*] denote the set of integers *i* with $a\le i\le b$.

### Graphs

A *graph* stands for a simple undirected graph, where an edge joining two vertices *u* and *v* is denoted by *uv* $(= vu)$. The sets of vertices and edges of a graph *H* are denoted by *V*(*H*) and *E*(*H*), respectively. Let $H=(V,E)$ be a graph with a set *V* of vertices and a set *E* of edges. For a vertex $v\in V$, the set of neighbors of *v* in *H* is denoted by $N_H(v)$, and the *degree* $\deg _H(v)$ of *v* is defined to be $|N_H(v)|$. The length of a path is defined to be the number of edges in the path. The *distance* ${\text {dist}}_H(u,v)$ between two vertices $u,v\in V$ is defined to be the minimum length of a path connecting *u* and *v* in *H*. The *diameter* ${\text {dia}}(H)$ of *H* is defined to be the maximum distance between two vertices in *H*; i.e., ${\text  {dia}}(H)\triangleq \max _{u,v\in V}{\text  {dist}}_H(u,v)$. Denote by $\ell (P)$ the length of a path *P*.

*Centers of trees* For a tree *T* with an even (resp., odd) diameter *d*, the *center* is defined to be the vertex *v* (resp., the adjacent vertex pair $\{v,v'\}$) that situates in the middle of one of the longest paths, with length *d*. The center of each tree is uniquely determined.

**Fig. 1:**
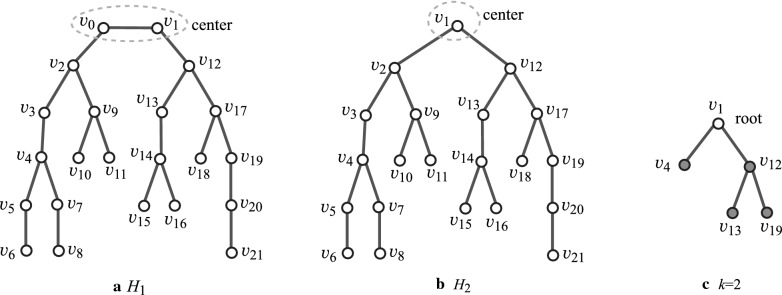
An illustration of rooted trees and a 2-branch-tree: **a** A tree $H_1$ with odd diameter 11; **b** A tree $H_2$ with even diameter 10; **c** The 2-branch-tree of $H_2$

*Rooted trees* A *rooted tree* is defined to be a tree where a vertex (or a pair of adjacent vertices) is designated as the *root*. Let *T* be a rooted tree, where for two adjacent vertices *u* and *v*, vertex *u* is called the parent of *v* if *u* is closer to the root than *v* is. The *height* ${\text  {height}}(v)$ of a vertex *v* in *T* is defined to be the maximum length of a path from *v* to a leaf *u* in the descendants of *v*, where ${\text  {height}}(v)=0$ for each leaf *v* in *T*. Figure 1a and b illustrate examples of trees rooted at the center.

*Degree-bounded trees* For positive integers *a*, *b* and *c* with $b\ge 2$, let *T*(*a*, *b*, *c*) denote the rooted tree such that the number of children of the root is *a*, the number of children of each non-root internal vertex is *b* and the distance from the root to each leaf is *c*. We see that the number of vertices in *T*(*a*, *b*, *c*) is $a(b^c-1)/(b-1)+1$, and the number of non-leaf vertices in *T*(*a*, *b*, *c*) is $a(b^{c-1}-1)/(b-1)+1$. In the rooted tree *T*(*a*, *b*, *c*), we denote the vertices by $v_1,v_2,\ldots ,v_n$ with a breadth-first-search order, and denote the edge between a vertex $v_i$ with $i\in [2,n]$ and its parent by $e_i$, where $n=a(b^c-1)/(b-1)+1$ and each vertex $v_i$ with $i\in [1, a(b^{c-1}-1)/(b-1)+1]$ is a non-leaf vertex. For each vertex $v_i$ in *T*(*a*, *b*, *c*), let $\text  {Cld}(i)$ denote the set of indices *j* such that $v_j$ is a child of $v_i$, and $\text  {prt}(i)$ denote the index *j* such that $v_j$ is the parent of $v_i$ when $i\in [2,n]$. Let $P_{\text  {prc}}(a,b,c)$ be a set of ordered index pairs (*i*, *j*) of vertices $v_i$ and $v_j$ in *T*(*a*, *b*, *c*). We call $P_{\text  {prc}}(a,b,c)$ *proper* if the next conditions hold:

- For each pair of vertices $v_i$ and $v_j$ in *T*(*a*, *b*, *c*) such that $v_i$ is the parent of $v_j$, there is a sequence $(i_1,i_2),(i_2,i_3),\ldots ,(i_{k-1},i_k)$ of index pairs in $P_{\text  {prc}}(a,b,c)$ such that $i_1=i$ and $i_k=j$; and
- Each subtree $H=(V,E)$ of *T*(*a*, *b*, *c*) with $v_1\in V$ is isomorphic to a subtree $H'=(V',E')$ by a graph isomorphism $\psi :V\rightarrow V'$ with $\psi (v_1)=v_1$ so that if $v_j\in V'$ for a pair $(i,j)\in P_{\text  {prc}}(a,b,c)$ then $v_i\in V'$.

Note that a proper set $P_{\text  {prc}}(a,b,c)$ is not necessarily unique.

*Branch-height in trees* In this paper, we introduce “branch-height” of a tree as a new measure to the “agglomeration degree” of trees. We specify a non-negative integer *k*, called a *branch-parameter* to define branch-height. First we regard *T* as a rooted tree by choosing the center of *T* as the root. Figure 1a, b illustrate examples of rooted trees. We introduce the following terminology on a rooted tree *T*.

**Fig. 2:**
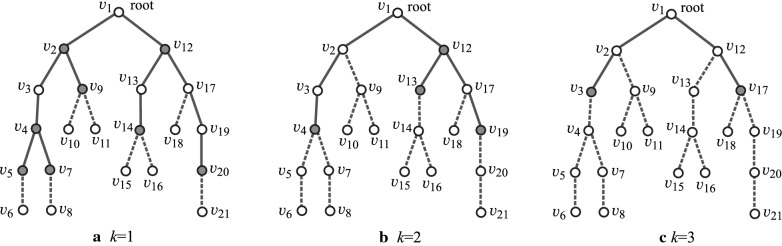
An illustration of the *k*-branches (depicted by gray circles), the *k*-branch-subtree (depicted by solid lines) and *k*-fringe-trees (depicted by dashed lines) of $H_2$: **a** $k=1$; **b** $k=2$; **c** $k=3$

- A *leaf* *k*-*branch*: A non-root vertex *v* in *T* such that ${\text  {height}}(v)= k$.
- A *non-leaf* *k*-*branch*: A non-root vertex *v* in *T* such that *v* has at least two children, and for each child *u* of *v* it holds that ${\text  {height}}(u)\ge k$. We call a leaf or a non-leaf *k*-branch a *k*-*branch*. Figure 2a–c illustrate the *k*-branches of the rooted tree $H_2$ in Fig. 1b for $k=1,2$ and 3, respectively.
- A *k*-*branch-path*: A path *P* in *T* that joins two vertices *u* and $u'$ such that each of *u* and $u'$ is the root or a *k*-branch and *P* does not contain the root or a *k*-branch as an internal vertex.
- The *k*-*branch-subtree* of *T*: The subtree of *T* that consists of the edges in all *k*-branch-paths of *T*. We call a vertex (resp., an edge) in *T* a *k*-*internal vertex* (resp., a *k*-*internal edge*) if it is contained in the *k*-branch-subtree of *T* and a *k*-*external vertex* (resp., a *k*-*external edge*) otherwise. Let $V^{\text  {in}}$ and $V^\text  {ex}$ (resp., $E^{\text  {in}}$ and $E^\text  {ex}$) denote the sets of *k*-internal and *k*-external vertices (resp., edges) in *T*.
- The *k*-*branch-tree* of *T*: The rooted tree obtained from the *k*-branch-subtree of *T* by replacing each *k*-branch-path with a single edge. Figure 1c illustrates the 2-branch-tree of the rooted tree $H_2$ in Fig. 1b. Notice that by our definitions, leaf *k*-branches and non-leaf *k*-branches are leaves and branching points in the *k*-branch-tree.
- A *k*-*fringe-tree*: One of the connected components that consists of the edges not in the *k*-branch-subtree. Each *k*-fringe-tree $T'$ contains exactly one vertex *v* in the *k*-branch-subtree, where $T'$ is regarded as a tree rooted at *v*. Note that the height of any *k*-fringe-tree is at most *k*. Figure 2a–c illustrate the *k*-fringe-trees of the rooted tree $H_2$ in Fig. 1b for $k=1, 2$ and 3, respectively.
- The *k*-*branch-leaf number* ${\text  {bl}}_k(T)$: The number of leaf *k*-branches in *T*. For the trees $H_i$, $i=1,2$ in Fig. 1a, b, it holds that ${\text  {bl}}_0(H_1)= {\text  {bl}}_0(H_2)=8$, ${\text  {bl}}_1(H_1)= {\text  {bl}}_1(H_2)=5$, ${\text  {bl}}_2(H_1)= {\text  {bl}}_2(H_2)=3$ and ${\text  {bl}}_3(H_1)= {\text  {bl}}_3(H_2)=2$.
- The *k*-*branch height* $\text  {bh}_k(T)$ of *T*: The maximum number of *k*-branches along a path from the root to a leaf of *T*; i.e., $\text  {bh}_k(T)$ is the height of the *k*-branch-tree $T^*$ (the maximum length of a path from the root to a leaf in $T^*$). For the example of trees $H_i$, $i=1,2$ in Fig. 1a, b, it holds that $\text  {bh}_0(H_1)=\text  {bh}_0(H_2)=3$, $\text  {bh}_1(H_1)=\text  {bh}_1(H_2)=3$, $\text  {bh}_2(H_1)=\text  {bh}_2(H_2)=2$ and $\text  {bh}_3(H_1)=\text  {bh}_3(H_2)=1$.

Even though this paper deals exclusively with acyclic graphs, we formally introduce the *k*-branch height for chemical cyclic graphs (chemical graphs that contain at least one cycle). The *core* of a chemical cyclic graph *G* is defined to be the induced subgraph $G'$ of *G* that consists of vertices in a cycle or the vertices in a path joining two cycles. A vertex in the core (not in the core) is called a *core vertex* (resp., a non-core vertex). The edges not in the core of a chemical cyclic graph *G* form a collection of trees *T*, which we call a *non-core tree*. Each non-core tree contains exactly one core vertex and is regarded as a tree rooted at the core vertex. The *k*-*branch height* of a chemical cyclic graph *G* is defined to be the maximum of *k*-branch heights over all non-core trees. We observe that most chemical graphs *G* with at most 50 non-hydrogen atoms satisfy $\text  {bh}_2(G)\le 2$. See Appendix A for a summary of statistical feature distribution of chemical graphs registered in the chemical database PubChem [25].

For convenient reference, we summarize the graph-related notation used throughout this paper in Table 1.

**Table 1:** Graph-theoretic notation

Symbol	Designation
General graph notation	
$H = (V, E)$	A graph *H* with a vertex set *V* and edge set *E*
*V*(*H*)	The vertex set of a graph *H*
*E*(*H*)	The edge set of a graph *H*
$N_H(v)$	The number of neighbors of a vertex *v* in a graph *H*
$\deg _H(v)$	The degree $|N_H(v)|$ of a vertex *v* in a graph *H*
${{\text {dist}}}_H(u, v)$	The distance between two vertices *u* and *v* in a graph *H*
$\text {dia}(H)$	The diameter of a graph *H*
$\ell (P)$	The length of a path *P*
Branch-height in a tree *T*	
$V^{{\text {in}}}$	The set of internal vertices for a fixed branch parameter *k*
$V^{\text {ex}}$	The set of external vertices for a fixed branch parameter *k*
$E^{{\text {in}}}$	The set of internal edges for a fixed branch parameter *k*
$E^{\text {ex}}$	The set of external edges for a fixed branch parameter *k*
${\text {bl}}_k(T)$	The *k*-branch-leaf number of *T*
$\text {bh}_k(T)$	The *k*-branch height of *T*

### Modeling of chemical compounds

We represent the graph structure of a chemical compound as a graph with labels on vertices and multiplicity on edges in a hydrogen-suppressed model. Let $\Lambda$ be a set of labels each of which represents a chemical element such as C (carbon), O (oxygen), N (nitrogen) and so on, where we assume that $\Lambda$ does not contain H (hydrogen). Let $\text  {mass}({\mathtt{a}})$ and ${\text  {val}}({\mathtt{a}})$ denote the mass and valence of a chemical element ${\mathtt{a}}\in \Lambda$, respectively. In our model, we use integer $\text  {mass}^*({\mathtt{a}})=\lfloor 10\cdot \text  {mass}({\mathtt{a}})\rfloor$, ${\mathtt{a}}\in \Lambda$, and assume that each chemical element ${\mathtt{a}}\in \Lambda$ has a unique valence ${\text  {val}}({\mathtt{a}})\in [1, 4]$.

We introduce a total order < over the elements in $\Lambda$ according to their mass values; i.e., we write $\mathtt{a<b}$ for chemical elements $\mathtt{a,b}\in \Lambda$ with $\text  {mass}({\mathtt{a}})<\text  {mass}(\mathtt{b})$. A pair of two atoms ${\mathtt{a}}$ and $\mathtt{b}$, $\mathtt{a, b} \in \Lambda$, joined with a bond-multiplicity $m \in [1, 3]$, where $m=1, 2, 3,$ correspond to single, double, and triple bonds, respectively, is denoted by a tuple $\gamma =(\mathtt{a, b}, m)$, called the *adjacency-configuration* of the atom pair. Choose a set $\Gamma _{<}$ of tuples $\gamma =(\mathtt{a,b},m)\in \Lambda \times \Lambda \times [1,3]$ such that $\mathtt{a<b}$. For a tuple $\gamma =(\mathtt{a,b},m)\in \Lambda \times \Lambda \times [1,3]$, let $\overline{\gamma }$ denote the tuple $(\mathtt{b,a},m)$. Set $\Gamma _{>}=\{\overline{\gamma }\mid \gamma \in \Gamma _{<}\}$ and $\Gamma _{=}=\{(\mathtt{a,a},m)\mid {\mathtt{a}}\in \Lambda , m\in [1,3]\}$, and $\Gamma = \Gamma _{<}\cup \Gamma _{=}$.

We use a hydrogen-suppressed model because hydrogen atoms can be added at the final stage.

Let $(H,\alpha ,\beta )$ be a tuple of a graph $H=(V,E)$, a function $\alpha :V\rightarrow \Lambda$ and a function $\beta : E\rightarrow [1,3]$, where $\alpha (v)={\mathtt{a}}$ and $\beta (e)=m$ mean that a chemical element ${\mathtt{a}}$ is assigned to a vertex *v* and a bond-multiplicity *m* is assigned to an edge *e*, respectively. For a notational convenience, we denote the sum of bond-multiplicities of edges incident to a vertex $u \in V$ by

- $$\beta (u) \triangleq \sum _{uv \in E}\beta (uv)$$.

A tuple $G=(H,\alpha ,\beta )$ is called a *chemical graph* over $\Lambda$ and $\Gamma _{<}\cup \Gamma _{=}$ if the following holds:

(i) *H* is connected;
(ii) $(\alpha (u),\alpha (v),\beta (uv))\in \Gamma _{<}\cup \Gamma _{=}$ for each edge $uv\in E$; and
(iii) $\beta (u) \le {\text  {val}}(\alpha (u))$ for each vertex $u\in V$.

A chemical graph $G=(H,\alpha ,\beta )$ is called a “chemical acyclic graph” if the graph *H* is an acyclic graph. Similarly for other types of graphs for *H*.

We define the *bond-configuration* of an edge $e=uv \in E$ in a chemical graph *G* to be a tuple $(\deg _H(u),\deg _H(v),\beta (e))$ such that $\deg _H(u)\le \deg _H(v)$ for the end-vertices *u* and *v* of *e*. Let $\text  {Bc}$ denote the set of bond-configurations $\mu =(d_1,d_2,m)\in [1,4]\times [1,4]\times [1,3]$ such that $\max \{d_1,d_2\}+m \le 5$. We regard that $(d_1,d_2,m)=(d_2,d_1,m)$.

In summary, we give the notation on modeling chemical compounds used throughout this paper in Table 2.

**Table 2:** Notation adopted for modeling chemical compounds

Symbol	Designation
$\Lambda$	A set of labels representing chemical elements
$\text {mass}({\mathtt{a}})$	Atomic mass of chemical element ${\mathtt{a}}\in \Lambda$
${\text {val}}({\mathtt{a}})$	Valence of chemical element ${\mathtt{a}}\in \Lambda$
$\text {mass}^*({\mathtt{a}})$	$\lfloor 10\cdot \text {mass}({\mathtt{a}})\rfloor$, ${\mathtt{a}}\in \Lambda$
${\mathtt{a}}< \mathtt{b}$	A total order over labels in the set $\Lambda$, indicating $\text {mass}({\mathtt{a}}) < \text {mass}(\mathtt{b})$
$\gamma = ({\mathtt{a}}, \mathtt{b}, m)$	Adjacency configuration for an atom pair, ${\mathtt{a}}, \mathtt{b}\in \Lambda$, $m \in [1, 3]$
$\overline{\gamma }$	For an adjacency configuration $\gamma = ({\mathtt{a}}, \mathtt{b}, m)$, $\overline{\gamma } = (\mathtt{b}, {\mathtt{a}}, m)$
$\Gamma _<$	Set of adjacency configurations $\gamma = ({\mathtt{a}}, \mathtt{b}, m) \in \Lambda \times \Lambda \times [1, 3]$ with ${\mathtt{a}}< \mathtt{b}$
$\Gamma _>$	Set of adjacency configurations $\Gamma _> = \{ \overline{\gamma } \mid \gamma \in \Gamma _< \}$
$\Gamma _=$	Set of adjacency configurations, $\Gamma _= = \{ ({\mathtt{a}}, {\mathtt{a}}, m) \mid {\mathtt{a}}\in \Lambda , m = [1, 3] \}$
$\Gamma$	$\Gamma = \Gamma _< \cup \Gamma _=$
$\alpha$	A mapping of atom labels in $\Lambda$ to graph vertices
$\beta$	A mapping of integers in [1, 3] to graph edges, overloaded as $\beta (u) = \sum _{uv \in E(H)} \beta (uv)$ for vertices $u \in V(H)$ in a graph *H*
$\text {Bc}$	Set of bond-configurations $\mu \in [1, 4] \times [1, 4] \times [1, 3]$

### Descriptors

In our method, we use only graph-theoretical descriptors for defining a feature vector, which facilitates our design of an algorithm for constructing graphs. Given a chemical acyclic graph $G=(H,\alpha ,\beta )$, we define a *feature vector* *f*(*G*) that consists of the following 11 kinds of descriptors. We choose an integer $k^*\in [1,4]$ as a branch-parameter.

*General chemical graph descriptors*

- *n*(*G*): the number |*V*| of vertices.
- $\overline{\text  {dia}}(G)\triangleq \text  {dia}(H)/n(G)$: the diameter of *H* divided by $n(G)=|V|$.
- $\overline{\text  {ms}}\triangleq \sum _{v\in V}\text  {mass}^*(\alpha (v))/n(G)$: the average $\hbox {mass}^*$ of atoms in *G*.
- $n_\mathtt{H}(G)$: the number of hydrogen atoms to be added to *G*.

*Descriptors for vertices of certain degree*

- $$\text  {dg}_i^\text  {t}(G)\triangleq |\{v\in V^\text  {t}\mid \deg _{H}(v)=i\}|,$$$$i\in [1,4],$$$$\text  {t}\in \{{\text  {in}},\text  {ex}\}$$: the number of $$k^*$$-internal/$$k^*$$-external vertices of degree *i* in *H*, where the bond-multiplicity of edges incident to a vertex *v* is ignored in the degree of *v*.

*Descriptors for branch-leaf number and branch-height*

- ${\text  {bl}}_{k^*}(G)$: the $k^*$-branch-leaf number of *G*.
- $\text  {bh}_{k^*}(G)$: the $k^*$-branch height of *G*.

*Descriptors for vertex labels*

- $$\text  {ce}_{\mathtt{a}}^\text  {t}(G)\triangleq |\{ v\in V^\text  {t}\mid \alpha (v)={\mathtt{a}}\}|,$$$${\mathtt{a}}\in \Lambda,$$$$\text  {t}\in \{{\text  {in}},\text  {ex}\}$$: the number of $$k^*$$-internal/$$k^*$$-external vertices with chemical element $${\mathtt{a}}\in \Lambda $$.

*Descriptors for the number of bonds*

- $\text  {bd}_m^\text  {t}(G)\triangleq \{e\in E^\text  {t}\mid \beta (e)=m\}$, $m=2, 3$, $\text  {t}\in \{{\text  {in}},\text  {ex}\}$: the number of $k^*$-internal/$k^*$-external edges with bond-multiplicity *m*.

*Descriptors for adjacency-configurations*

- $\text  {ac}_{\gamma }^\text  {t}(G)$, $\gamma \in \Gamma$, $\text  {t}\in \{{\text  {in}},\text  {ex}\}$: the number of $k^*$-internal/$k^*$-external edges $e=uv$ with adjacency-configuration $\gamma =(\mathtt{a,b},m)$ (i.e., $\alpha (u)={\mathtt{a}},\alpha (v)=\mathtt{b}$ and $\beta (e)=m$) in *G*.

*Descriptors for bond-configurations*

- $\text  {bc}_{\mu }^\text  {t}(G)$, $\mu \in \text  {Bc}$, $\text  {t}\in \{{\text  {in}},\text  {ex}\}$: the number of $k^*$-internal/$k^*$-external edges $e=uv$ with bond-configuration $\mu =(d,d',m)$ (i.e., $\deg _H(u)=d, \deg _H(v)=d'$ and $\beta (e)=m$) in *G*.

Note that

$$\begin{aligned} n_\mathtt{H}(G)&\triangleq \sum _{\begin{array}{c} {\mathtt{a}}\in \Lambda , \\ \mathtt{t} \in \{{\text  {in}},\text  {ex}\} \end{array}} {\text  {val}}({\mathtt{a}})\text  {ce}_{\mathtt{a}}^\text  {t}(G) - \sum _{\begin{array}{c} \gamma =(\mathtt{a, b}, m)\in \Gamma ,\\ \mathtt{t} \in \{{\text  {in}},\text  {ex}\} \end{array}} 2m\cdot \text  {ac}_{\gamma }^\text  {t}(G) \\&= \sum _{\begin{array}{c} {\mathtt{a}}\in \Lambda , \\ \mathtt{t}\in \{{\text  {in}},\text  {ex}\} \end{array}} {\text  {val}}({\mathtt{a}})\text  {ce}_{\mathtt{a}}^\text  {t}(G) -2(n(G)-1 + \sum _{\begin{array}{c} m\in [2,3], \\ \mathtt{t}\in \{{\text  {in}},\text  {ex}\} \end{array}} (m-1) \cdot \text  {bd}_m^\text  {t}(G)). \end{aligned}$$

The number *K* of descriptors in our feature vector $x=f(G)$ is $K=2|\Lambda |+2|\Gamma |+50$. Note that the above *K* descriptors are not independent in the sense that some descriptors depend on the combination of other descriptors. For example, descriptor $\text  {bd}_i^{\text  {in}}(G)$ can be determined by $\sum _{\gamma =(\mathtt{a,b},m)\in \Gamma : m=i }\text  {ac}_{\gamma }^{\text  {in}}(G)$.

## A method for inferring chemical graphs

### Framework for the Inverse QSAR/QSPR

**Fig. 3:**
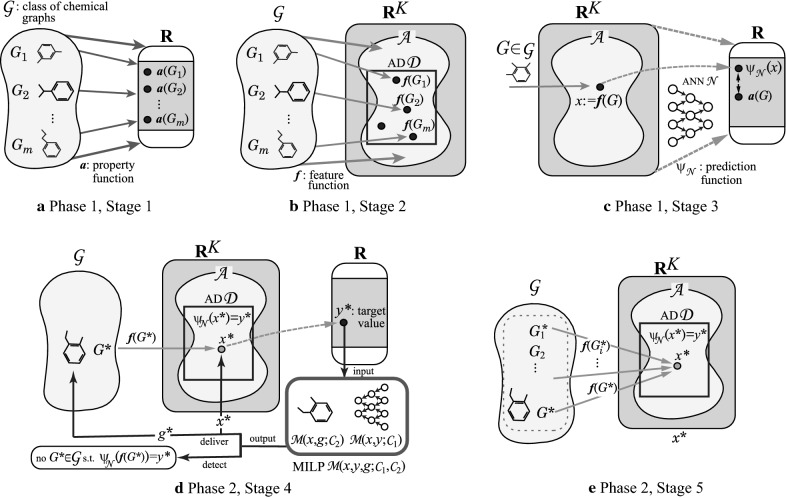
**a**–**c** An illustration of Phase 1: **a** Stage 1 for preparing a data set $D_{\pi }$ for a graph class ${\mathcal {G}}$ and a specified chemical property $\pi$; **b** Stage 2 for introducing a feature function *f* with descriptors; **c** Stage 3 for constructing a prediction function $\psi _{{\mathcal {N}}}$ with an ANN ${{\mathcal {N}}}$; **d**–**e** An illustration of Phase 2: (d) Stage 4 for formulating an MILP ${{\mathcal {M}}}(x,y,g;{\mathcal {C}}_1,{\mathcal {C}}_2)$ and finding a feasible solution $(x^*,g^*)$ of the MILP for a target value $y^*$ so that $\psi _{{\mathcal {N}}}(x^*)=y^*$ (possibly detecting that no target graph $G^*$ exists); (e) Stage 5 for enumerating graphs $G^*\in {\mathcal {G}}$ such that $f(G^*)=x^*$

We review the framework that solves the inverse QSAR/QSPR by using MILPs [20, 21], which is illustrated in Fig. 3. For a specified chemical property $\pi$ such as boiling point, we denote by *a*(*G*) the observed value of the property $\pi$ for a chemical compound *G*. As the first phase, we solve (I) Prediction Problem with the following three steps.

*Phase 1.*

Stage 1: Let $\text  {DB}$ be a set of chemical graphs. For a specified chemical property $\pi$, choose a class ${\mathcal {G}}$ of graphs such as acyclic graphs or monocyclic graphs. Prepare a data set $D_{\pi }=\{G_i\mid i=1,2,\ldots ,m\}\subseteq {\mathcal {G}}\cap \text  {DB}$ such that the value $a(G_i)$ of each chemical graph $G_i$, $i=1,2,\ldots ,m$ is available. Set reals ${\underline{a}}, {\overline{a}}\in {\mathbb {R}}$ so that ${\underline{a}}\le a(G_i)\le {\overline{a}}$, $i=1,2,\ldots ,m$.

Stage 2: Introduce a feature function $f: {\mathcal {G}}\rightarrow {\mathbb {R}}^K$ for a positive integer *K*. We call *f*(*G*) the *feature vector* of $G\in {\mathcal {G}}$, and call each entry of a vector *f*(*G*) a *descriptor* of *G*.

Stage 3: Construct a prediction function $\psi _{{\mathcal {N}}}$ with an ANN ${{\mathcal {N}}}$ that, given a vector in ${\mathbb {R}}^K$, returns a real number in the range $[{\underline{a}},{\overline{a}}]$ so that $\psi _{{\mathcal {N}}}(f(G))$ takes a value nearly equal to *a*(*G*) for many chemical graphs in $\text  {DB}$. See Fig. 3a–c for an illustration of Stages 1, 2, and 3 in Phase 1.

In this paper, we use the range-based method to define an applicability domain (AD) [26] to our inverse QSAR/QSPR. Set $\underline{x_j}$ and $\overline{x_j}$ to be the minimum and maximum values of the *j*-th descriptor $x_j$ in $f(G_i)$, respectively, over all graphs $G_i$, $i=1,2,\ldots ,m$, where we possibly normalize some descriptors such as $\text  {ce}_{\mathtt{a}}^{\text  {in}}(G)$, which is normalized with $\text  {ce}_{\mathtt{a}}^{\text  {in}}(G)/n(G)$. Define our AD ${\mathcal {D}}$ to be the set of vectors $x\in {\mathbb {R}}^K$ such that $\underline{x_j}\le x_j\le \overline{x_j}$ for the variable $x_j$ of each *j*-th descriptor, $j=1,2,\ldots ,k$.

In the second phase, we try to find a vector $x^*\in {\mathbb {R}}^K$ from a target value $y^*$ of the chemical propery $\pi$ such that $\psi _{{\mathcal {N}}}(x^*)=y^*$. Based on the method due to Akutsu and Nagamochi [16], Chiewvanichakorn et al. [18] showed that this problem can be formulated as an MILP. By including a set of linear constraints such that $x\in {\mathcal {D}}$ into their MILP, we obtain the next result.

#### **Theorem 1**

([20, 21]) *Let* ${{\mathcal {N}}}$ *be an ANN with a piecewise-linear activation function for an input vector* $x\in {\mathbb {R}}^K,$ $n_A$ *denote the number of nodes in the architecture and* $n_B$ *denote the total number of break-points over all activation functions. Then there is an MILP* ${{\mathcal {M}}}(x,y;{\mathcal {C}}_1)$ *that consists of variable vectors* $x\in {\mathcal {D}}~(\subseteq {\mathbb {R}}^K)$, $y\in {\mathbb {R}}$*, and an auxiliary variable vector* $z\in {\mathbb {R}}^p$ *for some integer* $p=O(n_A+n_B)$ *and a set* ${\mathcal {C}}_1$ *of* $O(n_A+n_B)$ *constraints on these variables such that:* $\psi _{{{\mathcal {N}}}}(x^*)=y^*$ *if and only if there is a vector* $(x^*,y^*)$ *feasible to* ${{\mathcal {M}}}(x,y;{\mathcal {C}}_1)$.

See Appendix “Upper and lower bounds on descriptors” for the set of constraints to define our AD ${\mathcal {D}}$ in the MILP ${{\mathcal {M}}}(x,y;{\mathcal {C}}_1)$ in Theorem 1.

A vector $x\in {\mathbb {R}}^K$ is called *admissible* if there is a chemical graph $G\in {\mathcal {G}}$ such that $f(G)=x$ [17]. Let ${\mathcal {A}}$ denote the set of admissible vectors $x\in {\mathbb {R}}^K$. To ensure that a vector $x^*$ inferred from a given target value $y^*$ becomes admissible, we introduce a new vector variable $g\in {\mathbb {R}}^{q}$ for an integer *q*. For the class ${\mathcal {G}}$ of chemical acyclic graphs, Azam et al. [17] introduced a set ${\mathcal {C}}_2$ of new constraints with a new vector variable $g\in {\mathbb {R}}^{q}$ for an integer *q* so that

- A feasible solution $(x^*,g^*)$ of a new MILP for a target value $y^*$ delivers a vector $x^*$ with $\psi _{{{\mathcal {N}}}}(x^*)=y^*$, and
- A vector $g^*$ that represents a chemical acyclic graph $G^*\in {\mathcal {G}}$.

Afterwards, for the classes of chemical graphs with cycle index 1 and 2, Ito et al. [17] and Zhu et al. [21] presented such a set ${\mathcal {C}}_2$ of constraints so that a vector $g^*$ in a feasible solution $(x^*,g^*)$ of a new MILP can represent a chemical graph $G^*$ in the class ${\mathcal {G}}$, respectively.

As the second phase, we solve (II) Inverse Problem for the inverse QSAR/QSPR by treating the following inference problems.

(II-a) Inference of Vectors

Input: A real $y^*$ with ${\underline{a}}\le y^*\le {\overline{a}}$.

Output: Vectors $x^*\in {\mathcal {A}}\cap {\mathcal {D}}$ and $g^*\in {\mathbb {R}}^{q}$ such that $\psi _{{\mathcal {N}}}(x^*)=y^*$ and $g^*$ forms a chemical graph $G^*\in {\mathcal {G}}$ with $f(G^*)=x^*$.

(II-b) Inference of Graphs

Input: A vector $$x^*\in {\mathcal {A}}\cap {\mathcal {D}}$$.

Output: All graphs $G^*\in {\mathcal {G}}$ such that $f(G^*)=x^*$.

The second phase consists of the next two steps.

*Phase 2.*

Stage 4:  Formulate Problem (II-a) as the above MILP ${{\mathcal {M}}}(x,y,g;{\mathcal {C}}_1,{\mathcal {C}}_2)$ based on ${\mathcal {G}}$ and ${{\mathcal {N}}}$. Find a feasible solution $(x^*,g^*)$ of the MILP such that

- $$x^*\in {\mathcal {A}}\cap {\mathcal {D}}$$ and $$\psi _{{\mathcal {N}}}(x^*)=y^*$$.

The second requirement may be replaced with inequalities $(1-\varepsilon )y^* \le \psi _{{\mathcal {N}}}(x^*) \le (1+\varepsilon )y^*$ for a tolerance $\varepsilon >0.$

Stage 5:  To solve Problem (II-b), enumerate all (or a specified number) of graphs $G^*\in {\mathcal {G}}$ such that $f(G^*)=x^*$ for the inferred vector $x^*$. See Fig. 3d, e for an illustration of Stages 4 and 5 in Phase 2.

In practical applications, there would be many criteria that a target chemical compound needs to satisfy rather than a single chemical property $\pi$, such as stability and synthesizability. The above five steps in the framework are rather schematic in the sense that it would be necessary to adjust several settings in each stage in order to find a collection of chemical graphs that meet many of those criteria after a repeated application of the framework. For example, we can include in an MILP formulation in Stage 4 additional conditions such as lower and upper bounds on the frequency of adjacency-configurations and extra requirements on substructures of a target chemical graph as long as these conditions can be expressed as linear constraints with integer/real variables. Also an efficient algorithm in Stage 5 can quickly offer a large number of isomers of the same feature vectors, to which we can apply a further screening to choose promising candidates for chemical graphs.

### Our target graph class

In this paper, we choose a branch-parameter $k\ge 1$ and define a class ${\mathcal {G}}$ of chemical acyclic graphs *G* such that

- The maximum degree in *G* is at most 4;
- The *k*-branch height $\text  {bh}_k(G)$ is bounded for a specified branch-parameter *k*; and
- The size of each *k*-fringe-tree in *G* is bounded.

The reason why we restrict ourselves to the graphs in ${\mathcal {G}}$ is that this class ${\mathcal {G}}$ covers a large part of the acyclic chemical compounds registered in the chemical database PubChem. See Appendix A for a summary of the statistical features of the chemical graphs in PubChem in terms of *k*-branch height and the size of 2-fringe-trees. According to this, over 55% (resp., 99%) of acyclic chemical compounds with up to 100 non-hydrogen atoms in PubChem have the maximum degree 3 (resp., 4); and nearly 87% (resp., 99%) of acyclic chemical compounds with up to 50 non-hydrogen atoms in PubChem have the 2-branch height at most 1 (resp., 2). This implies that $k=2$ is sufficient to cover most of chemical acyclic graphs. For $k=2$, over 92% of 2-fringe-trees of chemical compounds with up to 100 non-hydrogen atoms in PubChem obey the following size constraint:

1 $$\begin{aligned} n(T) \le 2\deg _T(r) + 2\hbox { for each 2-fringe-tree }T \hbox { with the root }r. \end{aligned}$$

We formulate an MILP in Stage 4 that, given a target value $y^*$, infers a vector $x^*\in {\mathbb {Z}}_+^K$ with $\psi _{{\mathcal {N}}}(x^*)=y^*$ and a chemical acyclic graph $G^*=(H,\alpha ,\beta )\in {\mathcal {G}}$ with $f(G^*)=x^*$. We here specify some of the features of a graph $G^*\in {\mathcal {G}}$ such as the number of non-hydrogen atoms in order to control the graph structure of target graphs to be inferred and to simplify MILP formulations. In this paper, we specify the following features on a graph $G\in {\mathcal {G}}$: a set $\Lambda$ of chemical elements, a set $\Gamma _{<}$ of adjacency-configurations, the maximum degree, the number of non-hydrogen atoms, the diameter, the *k*-branch height and the *k*-branch-leaf number for a branch-parameter *k*.

More formally, given specified integers $n^*$, $d_\text  {max}$, $\text  {dia}^*$, $k^*$, $\text  {bh}^*$, ${\text  {bl}}^*\in {\mathbb {Z}}$ other than $\Lambda$ and $\Gamma$, let ${\mathcal {H}}(n^*, d_\text  {max}, \text  {dia}^*, k^*, \text  {bh}^*, {\text  {bl}}^*)$ denote the set of acyclic graphs *H* such that

- The maximum degree of a vertex in *H* is at most 3 when $d_\text  {max}=3$ (or equal to 4 when $d_\text  {max}=4$),
- The number *n*(*H*) of vertices in *H* is $n^*$,
- The diameter $\text  {dia}(H)$ of *H* is $\text  {dia}^*$,
- The $k^*$-branch height $\text  {bh}_{k^*}(H)$ is $\text  {bh}^*$,
- The $k^*$-branch-leaf number ${\text  {bl}}_{k^*}(H)$ is ${\text  {bl}}^*$ and
- (1) holds.

To design Stage 4 for our class ${\mathcal {G}}$, we formulate an MILP ${{\mathcal {M}}}(x,g; {\mathcal {C}}_2)$ that infers a chemical graph $G^*=(H,\alpha ,\beta )\in {\mathcal {G}}$ with $H\in {\mathcal {H}}(n^*, d_\text  {max}, \text  {dia}^*, k^*, \text  {bh}^*, {\text  {bl}}^*)$ for a given specification $(\Lambda ,\Gamma ,n^*, d_\text  {max}, \text  {dia}^*, k^*, \text  {bh}^*, {\text  {bl}}^*)$. The details will be given in "MILPs for chemical acyclic graphs with bounded branch-height" section and Appendix C.

Design of Stage 5, i.e., generating chemical graphs $G^*$ that satisfy $f(G^*)=x^*$ for a given feature vector $x^*\in {\mathbb {Z}}_+^K$ is still challenging for a relatively large instance with size $n(G^*)\ge 20$. There have been proposed algorithms for generating chemical graphs $G^*$ in Stage 5 for the classes of graphs with cycle index 0 to 2 [5, 22–24]. All of these are designed based on the branch-and-bound method and can generate a target chemical graph with size $n(G^*)\le 20$. To break this barrier, we newly employ the dynamic programming method for designing an algorithm in Stage 5 in order to generate a target chemical graph $G^*$ with size $n(G^*)=50$. For this, we further restrict the structure of acyclic graphs *G* so that the number ${\text  {bl}}_2(G)$ of leaf 2-branches is at most 3. Among all acyclic chemical compounds with up to 50 non-hydrogen atoms in the chemical database PubChem, the ratio of the number of acyclic chemical compounds *G* with ${\text  {bl}}_2(G)\le 2$ (resp., ${\text  {bl}}_2(G)\le 3$) is 78% (resp., 95%). See "A new graph search algorithm" section and Appendix D for the details on the new algorithm in Stage 5.

To conclude the description of the target graph class to be inferred by the inverse QSAR/QSPR framework developed in this paper, we summarize the global parameters in Table 3.

**Table 3:** Fixed parameters of target graphs

Symbol	Designation
$\Lambda$	A set of atom labels
$\Gamma$	A set of adjacency configurations
$n^*$	Number of vertices
$d_{\max }$	Maximum vertex degree, at most 3 and exactly 4, for $d_{\max } = 3$ and $d_{\max } = 4$, respectively
$\text {dia}^*$	Graph diameter
$k^*$	Branch parameter
$\text {bh}^*$	$k^*$-branch height
${\text {bl}}^*$	$k^*$-branch-leaf number

## MILPs for chemical acyclic graphs with bounded branch-height

In this section, we describe an idea of formulating an MILP ${{\mathcal {M}}}(x,g;{\mathcal {C}}_2)$ to infer a chemical acyclic graph *G* in the class ${\mathcal {G}}$ for a given specification $(\Lambda ,\Gamma ,n^*, d_\text  {max}, \text  {dia}^*,$ $k^*, \text  {bh}^*, {\text  {bl}}^*)$ defined in the previous section. Please refer to Table 3 for a summary of the parameters that we assume to be fixed for a target graph.

### Scheme graphs

**Fig. 4:**
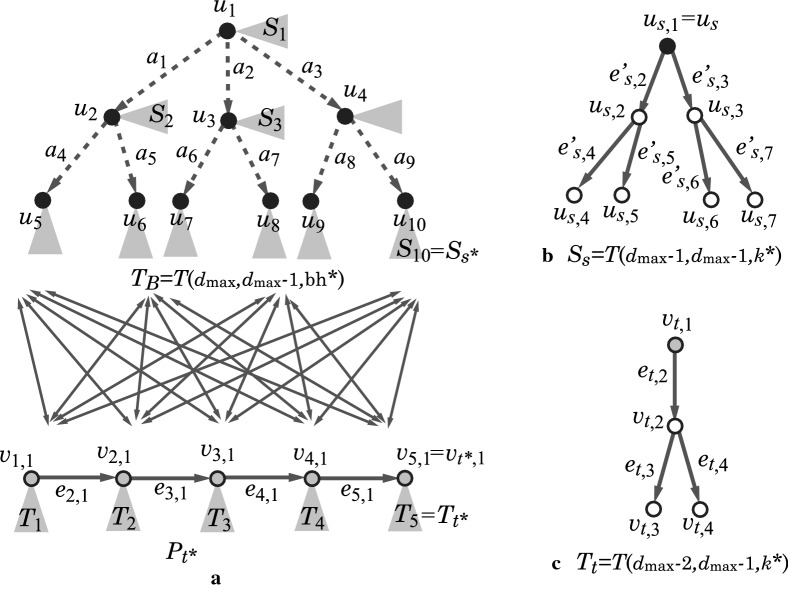
An illustration of scheme graph ${\text {SG}}( d_\text  {max}, k^*, {\text {bh}}^*, t^*)$ with $d_{\text {max}}=3$, $k^*=2$, ${\text {bh}}^*=2$, and $t^*=5$, where the vertices in $T_B$ (resp., in $P_{t^*}$) are depicted with black (resp., gray) circles: **a** A base-tree $T_B$ and a link-path $P_{t^*}$ are joined with directed edges between them; **b** A tree $S_s$ rooted at a vertex $u_s=u_{s,1}\in V_B$; **c** A tree $T_t$ rooted at a vertex $v_t=v_{t,1}\in V_P$

Our new idea of constructing an acyclic graph *H* is as follows. See a rooted tree $T_B=T(d_\text  {max},d_\text  {max}-1,\text  {bh}^*)$ in Fig. 4a.

- From the tree $T_B$, we first choose a subtree *T* including the root $u_1$. We use *T* as the $k^*$-branch-tree of *H*.
- Next, we choose some edges in the tree *T* and replace each of the edges $e=u_i u_j$ with a path $P_e$ between vertices $u_i$ and $u_j$. Let $T^*$ denote the resulting tree. We use $T^*$ as the $k^*$-branch-subtree of *H*.
- Finally, we append to the tree $T^*$ rooted trees with height at most *k* as the $k^*$-fringe-trees of *H*. The resulting tree is a required rooted tree *H*.

In our MILP, we prepare a binary variable for each of the vertices and edges in $T_B$ so that a subtree *T* of $T_B$ can be selected as one of the combinations of these binary values.

To represent a replacement of an edge *e* with a path $P_e$ in our MILP, we introduce a path $P_{t^*}=(v_{1,1},v_{2,1},\ldots ,v_{t^*,1})$ of a sufficiently large length $t^*-1$, and a set *F* of directed edges between the vertices in $T_B$ and $P_{t^*}$ as shown in Fig. 4a. We also introduce a binary variable for each of the vertices and edges in $P_{t^*}$ and *F* in our MILP. When an edge $e=u_i u_j$ is replaced with a path $P_e$, we select an edge from $u_i$ to a vertex $v_{h,1}$ in $P_{t^*}$ and an edge from a vertex $v_{h+p,1}$ so that the edges $(u_i,v_{h,1})$ and $(v_{h+p,1},u_j)$ and the subpath $(v_{h,1},v_{h+1,1},\ldots , v_{h+p,1})$ of $P_{t^*}$ form a path $P_e$. Such a path $P_e$ can be selected as one of the combinations of these binary values. To append rooted trees to tree $T^*$, we prepare a rooted tree with a sufficiently large size at each vertex in $T_B$ and $P_{t^*}$ and introduce a binary variable for each of the vertices and edges in these rooted trees in our MILP. A rooted subtree from each of such rooted trees as a $k^*$-fringe-tree can be selected as one of the combinations of these binary values.

We call the graph that consists of all the above graphs $T_B$, $P_{t^*}$ and the edge set *F* and the set of rooted trees at the vertices in $T_B$ and $P_{t^*}$ a *scheme graph* $\text  {SG}( d_\text  {max}, k^*, \text  {bh}^*, t^*)$.

**Fig. 5:**
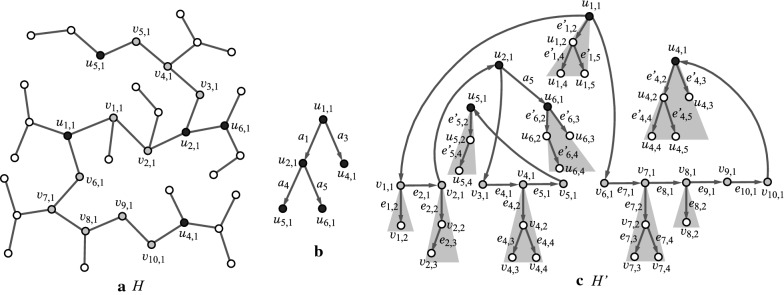
An illustration of selecting a subgraph *H* from the scheme graph $\text  {SG}( d_\text  {max}, k^*, \text  {bh}^*, t^*=n^*-{\text  {bl}}^*-1)$: **a** An acyclic graph $H\in {\mathcal {H}}(n^*, d_\text  {max}, \text  {dia}^*, k^*, \text  {bh}^*, {\text  {bl}}^*)$ with $n^*=37$, $d_\text  {max}=3$, $\text  {dia}^*(H)=17$, $k^*=2$, $\text  {bh}^*=2$ and ${\text  {bl}}^*=3$, where the labels of some vertices indicate the corresponding vertices in the scheme graph $\text  {SG}( d_\text  {max}, k^*, \text  {bh}^*, t^*)$; **b** The $k^*$-branch-tree of *H* for $k^*=2$; **c** An acyclic graph $H'$ selected from $\text  {SG}( d_\text  {max}, k^*, \text  {bh}^*, t^*)$ as a graph that is isomorphic to *H* in (**a**)

Figure 5a illustrates an acyclic graph *H* with $n(H)=37$, $\text  {dia}(H)=17$, $\text  {bh}_2(H)=2$ and ${\text  {bl}}_2(H)=3$, where the maximum degree of a vertex is 3. Figure 5b illustrates the 2-branch-tree of the acyclic graph *H* in Fig. 5a. Figure 5c illustrates a subgraph $H'$ of the scheme graph $\text  {SG}( d_\text  {max}, k^*, \text  {bh}^*, t^*=n^*-{\text  {bl}}^*-1)$ such that $H'$ is isomorphic to the acyclic graph *H* in Fig. 5a.

In this paper, we obtain the following result.

#### **Theorem 2**

*Let*$\Lambda$*be a set of chemical elements,*$\Gamma$*be a set of adjacency-configurations, where*$|\Lambda |\le |\Gamma |$*, and*$K = 2|\Lambda | + 2|\Gamma | + 50$. *Given non-negative integers*$n^*\ge 3$*,*$d_\text  {max}\in \{3,4\}$*,*$\text  {dia}^*\ge 3$*,*$k^*\ge 1$*,*$\text  {bh}^*\ge 1$*and*${\text  {bl}}^*\ge 2$*, there is an MILP*${{\mathcal {M}}}(x,g;{\mathcal {C}}_2)$*that consists of variable vectors*$x\in {\mathbb {R}}^K$*and*$g\in {\mathbb {R}}^q$*for an integer*$q=O( |\Gamma |\cdot [ (d_\text  {max}-1)^{\text  {bh}^*+k^*} +n^*\cdot (d_\text  {max}-1)^{\max \{\text  {bh}^*,k^*\}})])$*and a set*${\mathcal {C}}_2$*of constraints onxandgwith size*$O(|\Gamma | + (d_\text  {max}-1)^{\text  {bh}^*+k^*} +n^*\cdot (d_\text  {max}-1)^{\max \{\text  {bh}^*, k^*\}}) )$*such that:*$(x^*, g^*)$*is feasible to*${{\mathcal {M}}}(x,g; {\mathcal {C}}_2)$*if and only if*$g^*$*forms a chemical acyclic graph*$G=(H,\alpha ,\beta )$*such that*$H\in {\mathcal {H}}(n^*, d_\text  {max}, \text  {dia}^*, k^*, \text  {bh}^*,{\text  {bl}}^*)$*and*$f(G)=x^*$.

Note that our MILP requires only $O(n^*)$ variables and constraints when the branch-parameter $k^*$, the $k^*$-branch height and $|\Gamma |$ are constant.

See Appendices B and C for the details of the MILP formulation and the set of all variables and constraints in the MILP formulation, respectively.

## A new graph search algorithm

Previous methods of inferring chemical graphs [17–19] use a graph search algorithm based on the branch-and-bound algorithm proposed by Fujiwara et al. [5], where an enormous number of chemical graphs are constructed by repeatedly appending and removing a vertex one by one until a target chemical graph is constructed. Their algorithm cannot generate even one acyclic chemical graph when *n*(*G*) is larger than around 20.

This section introduces a new dynamic programming method for designing an algorithm in Stage 5. We consider the following aspects:

- Treat acyclic graphs with a certain limited structure that frequently appears among chemical compounds registered in the chemical database; and
- Instead of manipulating acyclic graphs directly, first compute the frequency vectors $\pmb {f}(G')$ (sub-vectors of the feature vectors $f(G')$, see Appendix D) of subtrees $G'$ of all target acyclic graphs and then construct a limited number of target graphs *G* from the process of computing the vectors.

**Fig. 6:**
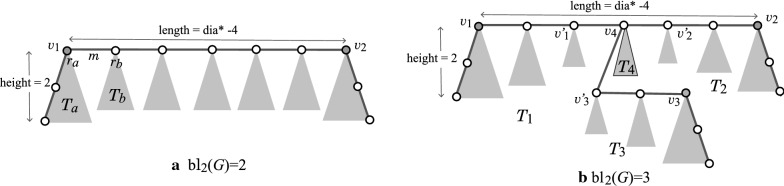
An illustration of chemical acyclic graphs *G* with diameter $\text  {dia}^*$ and ${\text  {bl}}_2(G)=2,3$: **a** A chemical acyclic graph *G* with two leaf 2-branches $v_1$ and $v_2$; **b** A chemical acyclic graph *G* with three leaf 2-branches $v_1, v_2$ and $v_3$

In (a), we choose a branch-parameter $k^*=2$ and treat acyclic graphs *G* that have a small 2-branch number such as ${\text  {bl}}_2(G)\in [2,3]$ and satisfy the size constraint (1) on 2-fringe-trees. Figure 6a, b illustrate chemical acyclic graphs *G* with ${\text  {bl}}_2(G)=2$ and ${\text  {bl}}_2(G)= 3$, respectively.

We design a method in (b) based on the mechanism of dynamic programming in the following way. Define a frequency vector $\pmb {f}(T)$ of each chemical rooted tree *T* to be a vector that consists of the frequency of each chemical element ${\mathtt{a}}\in \Lambda$, each adjacency-configuration ${\mathtt{a}}\in \Lambda$, each bond-configuration $\mu \in \text  {Bc}$, and each degree $\text  {dg}i\in \text  {Dg}$ in *T*. We are given a vector $\pmb {x}^*$ that is the frequency vector $\pmb {f}(G)$ of a chemical acyclic graph *G* to be inferred.

We first construct a set $\text  {FT}$ of chemical rooted trees with height at most $k^*=2$ and compute the frequency vector $\pmb {f}(T)$ of each chemical rooted tree $T\in \text  {FT}$ to obtain the set $\text  {W}(\text  {FT})$ of frequency vectors $\pmb {f}(T), T\in \text  {FT}$. Note that a large number of chemical rooted trees $T\in \text  {FT}$ maps to the same frequency vector $\pmb {w}$ and the size $|\text  {W}(\text  {FT})|$ is considerably smaller than the size $|\text  {FT}|$.

We next combine two chemical rooted trees $T_a,T_b\in \text  {FT}$ to construct a chemical tree $T_{a,b}$ by joining their roots $r_a$ and $r_b$ with an edge $e=r_a r_b$ of a bond-multiplicity *m*, as illustrated in Fig. 6a. In fact, we compute only the feature vector $\pmb {f}(T_{a,b})$ of such a tree $T_{a,b}$ without directly treating the graph structures of $T_a$, $T_b$ and $T_{a,b}$. For this, we add two frequency vectors $\pmb {w}_a,\pmb {w}_b\in \text  {W}(\text  {FT})$ together with an additional term from the bond-multiplicity *m* to obtain the frequency vector $\pmb {w}_{a,b}~(=\pmb {f}(T_{a,b}))$ of such a tree $T_{a,b}$. Given such a vector $\pmb {w}_{a,b}$, we can actually construct a chemical tree $T_{a,b}$ with $\pmb {f}(T_{a,b})=\pmb {w}_{a,b}$ by choosing trees $T_a,T_b\in \text  {FT}$ and combining them with an edge of bond-multiplicity *m*.

Our algorithm for generating a chemical acyclic graph *G* with ${\text  {bl}}_2(G)=2$ continues to compute a set $\text  {W}^{(p)}$ of frequency vectors of chemical trees that can be obtained by combining *p* trees in $\text  {FT}$ for each $p=2,3,\ldots , \lceil (\text  {dia}^*-5)/2\rceil$. Finally, we find a vector pair $(\pmb {w}^1,\pmb {w}^2)$ with $\pmb {w}^1\in \text  {W}^{(\lfloor (\text  {dia}^*-5)/2\rfloor )}$ and $\pmb {w}^2\in \text  {W}^{(\lceil (\text  {dia}^*-5)/2\rceil )}$ such that a vector with $\pmb {w}^1$, $\pmb {w}^2$ and a bond-multiplicity *m* is equal to the given vector $\pmb {x}^*$; i.e., a chemical acyclic graph *G* with $\pmb {f}(G)=\pmb {x}^*$ is obtained by joining chemical trees $T^1$ and $T^2$ with $\pmb {w}^i=\pmb {f}(T_i), i=1,2$ with an edge of bond-multiplicity *m*.

With a slight modification, the algorithm can generate a chemical acyclic graph *G* with ${\text  {bl}}_2(G)=3$.

Appendix D presents the details of our new algorithms for generating acyclic graphs *G* with ${\text  {bl}}_2(G)\in [2,3]$.

## Experimental results

We implemented our method of Stages 1 to 5 for inferring chemical acyclic graphs and conducted experiments to evaluate the computational efficiency for three chemical properties $\pi$: octanol/water partition coefficient (Kow), boiling point (Bp) and heat of combustion (Hc). We executed the experiments on a PC with Two Intel Xeon CPUs E5-1660 v3 @3.00GHz, 32 GB of RAM running under OS: Ubuntu 14.04.6 LTS. We show 2D drawings of some of the inferred chemical graphs, where ChemDoodle version 10.2.0 was used for constructing the drawings.

**Table 4:** Results of Stage 1 in Phase 1

$\pi$	$\Lambda$	$|D_{\pi }|$	$|\Gamma |$	$[{\underline{n}},{\overline{n}}]$	$[\underline{{\text {bl}}},\overline{{\text {bl}}}]$	$[\underline{\text {bh}},\overline{\text {bh}}]$	$[{\underline{a}},{\overline{a}}]$
Kow	C,O,N	216	10	[4, 28]	[0, 2]	[0, 4]	[− 4.2, 8.23]
Bp	C,O,N	172	10	[4, 26]	[0, 1]	[0, 3]	[− 11.7, 404.84]
Hc	C,O,N	128	6	[4, 26]	[0, 1]	[0, 2]	[1346.4, 13304.5]

*Results on Phase 1. * We implemented Stages 1, 2, and 3, in Phase 1 as follows.

Stage 1. We set a graph class ${\mathcal {G}}$ to be the set of all chemical acyclic graphs, and set a branch-parameter $k^*$ to be 2. For each property $\pi \in \{$ Kow, Bp, Hc$\}$, we first select a set $\Lambda$ of chemical elements and then collected a data set $D_{\pi }$ on chemical acyclic graphs over the set $\Lambda$ of chemical elements provided by the Hazardous Substances Data Bank (HSDB) of PubChem. To construct the data set, we eliminated chemical compounds that have at most three carbon atoms or contain a charged element such as $\mathtt{N}^+$ or an element ${\mathtt{a}}\in \Lambda$ whose valence is different from our setting of valence function ${\text  {val}}$.

Table 4 shows the size and range of data sets that we prepared for each chemical property in Stage 1, where we denote the following:

- $\pi$: one of the chemical properties Kow, Bp and Hc;
- $\Lambda$: the set of selected chemical elements (hydrogen atoms are added at the final stage);
- $|D_{\pi }|$: the size of data set $D_{\pi }$ over $\Lambda$ for property $\pi$;
- $|\Gamma |$: the number of different adjacency-configurations over the compounds in $D_{\pi }$;
- $[{\underline{n}},{\overline{n}}]$: the minimum and maximum number *n*(*G*) of non-hydrogen atoms over the compounds *G* in $D_{\pi }$;
- $[{\underline{{\text  {bl}}}},\overline{{\text  {bl}}}]$: the minimum and maximum numbers ${\text  {bl}}_2(G)$ of leaf 2-branches over the compounds *G* in $D_{\pi }$;
- $[{\underline{\text  {bh}}},\overline{\text  {bh}}]$: the minimum and maximum values of the 2-branch height $\text  {bh}_2(G)$ over the compounds *G* in $D_{\pi }$; and
- $[{\underline{a}},{\overline{a}}]$: the minimum and maximum values of *a*(*G*) for $\pi$ over compounds *G* in $D_{\pi }$.

Stage 2. We used a feature function *f* that consists of the descriptors defined in “Descriptors” section.

**Table 5:** Results of Stages 2 and 3 in Phase 1

$\pi$	*K*	Activation	Architecture	L-Time	test $\hbox {R}^2$ (ave.)	test $\hbox {R}^2$ (best)
Kow	76	ReLU	(76, 10, 1)	2.12	0.901	0.951
Bp	76	ReLU	(76, 10, 1)	26.07	0.935	0.965
Hc	68	ReLU	(68, 10, 1)	234.06	0.924	0.988

Stage 3. We used scikit-learn version 0.21.6 with Python 3.7.4 to construct ANNs ${{\mathcal {N}}}$ where the tool and activation function are set to be MLPRegressor and ReLU, respectively. We tested several different architectures of ANNs for each chemical property. To evaluate the performance of the resulting prediction function $\psi _{{\mathcal {N}}}$ with cross-validation, we partition a given data set $D_{\pi }$ into five subsets $D_{\pi }^{(i)}$, $i\in [1,5]$ randomly, where $D_{\pi }\setminus D_{\pi }^{(i)}$ is used for a training set and $D_{\pi }^{(i)}$ is used for a test set in five trials $i\in [1,5]$. For a set $\{y_1,y_2,\ldots ,y_N\}$ of observed values and a set $\{\psi _1,\psi _2,\ldots ,\psi _N\}$ of predicted values, we define the coefficient of determination to be $\text  {R}^2\triangleq 1- \frac{\sum _{j\in [1,N]}(y_j-\psi _j)^2}{\sum _{j\in [1,N]}(y_j-{\overline{y}})^2}$, where ${\overline{y}}= \frac{1}{N}\sum _{j\in [1,N]}y_j$. Table 5 shows the results on Stages 2 and 3, where

- *K*: the number of descriptors for the chemical compounds in data set $D_{\pi }$ for property $\pi$;
- Activation: the choice of activation function;
- Architecture: (*a*, *b*, 1) consists of an input layer with *a* nodes, a hidden layer with *b* nodes and an output layer with a single node, where *a* is equal to the number *K* of descriptors;
- L-time: the average time (in seconds) to construct ANNs for each trial;
- test $\text  {R}^2$ (ave.): the average of coefficient of determination over the five tests; and
- test $\text  {R}^2$ (best): the largest value of coefficient of determination over the five test sets.

From Table 5, we see that the execution of Stage 3 was successful, where the average of test $\text  {R}^2$ is over 0.9 for all three chemical properties.

For each chemical property $\pi$, we selected the ANN ${{\mathcal {N}}}$ that attained the best test $\text  {R}^2$ score among the five ANNs to formulate an MILP ${{\mathcal {M}}}(x,y,z;{\mathcal {C}}_1)$ which will be used in Phase 2.

*Results on Phase 2. * We implemented Stages 4 and 5 in Phase 2 as follows.

Stage 4. In this step, we solve the MILP ${{\mathcal {M}}}(x,y,g;{\mathcal {C}}_1,{\mathcal {C}}_2)$ formulated based on the ANN ${{\mathcal {N}}}$ obtained in Phase 1. To solve an MILP in Stage 4, we use CPLEX version 12.10. In our experiment, we choose a target value $y^* \in [{\underline{a}}, {\overline{a}}]$ and fix or bound some descriptors in our feature vector as follows:

- Set the 2-leaf-branch number ${\text  {bl}}^*$ to be each of 2 and 3;
- Fix the instance size $n^*=n(G)$ to be each integer in $\{26,32,38,44,50\}$;
- Set the diameter $\text  {dia}^*=\text  {dia}(G)$ be one of the integers in $\{ \lceil (2/5)n^*\rceil , \lceil (3/5)n^*\rceil \}$.
- Set the maximum degree $d_\text  {max}:=3$ for $\text  {dia}^*=\lceil (2/5)n^*\rceil$ and $d_\text  {max}:=4$ for $\text  {dia}^*= \lceil (3/5)n^*\rceil$;
- For each instance size $n^*$, test a target value $y^*_{\pi }$ for each chemical property $\pi \in \{$ Kow, Bp, Hc$\}$.

Based on the above setting, we generated six instances for each instance size $n^*$. We set $\varepsilon =0.02$ in Stage 4.

Tables 6, 7 (resp., Tables 8, 9) show the results on Stage 4 for ${\text  {bl}}^*=2$ (resp., ${\text  {bl}}^*=3$), where we denote the following:

- $y^*_{\pi }$: a target value in $[{\underline{a}},{\overline{a}}]$ for a property $\pi$;
- $n^*$: a specified number of vertices in $[{\underline{n}},{\overline{n}}]$;
- $\text  {dia}^*$: a specified diameter in $\{ \lceil (2/5)n^*\rceil , \lceil (3/5)n^*\rceil \}$;
- IP-time: the time (sec.) to an MILP instance to find vectors $x^*$ and $g^*$.

We observe that most of the MILP instances with ${\text  {bl}}^*=2$, $n^*\le 50$ and $\text  {dia}^*\le 30$ (resp., ${\text  {bl}}^*=3$, $n^*\le 50$ and $\text  {dia}^*\le 30$) are solved within one minute (resp., in a few minutes). The previously most efficient MILP formulation for inferring chemical acyclic graphs due to Zhang et al. [19] could solve instances with a relatively small diameter of $\text  {dia}^*=9$ for the case of $d_\text  {max}=4$ and $n^*=20$ and $\text  {dia}^*=8$ for the case of $d_\text  {max}=3$ and $n^*=50$. Our new MILP formulation on chemical acyclic graphs with bounded 2-branch height considerably improved the tractable size of chemical acyclic graphs in Stage 4 for the inference problem (II-a).

**Fig. 7:**
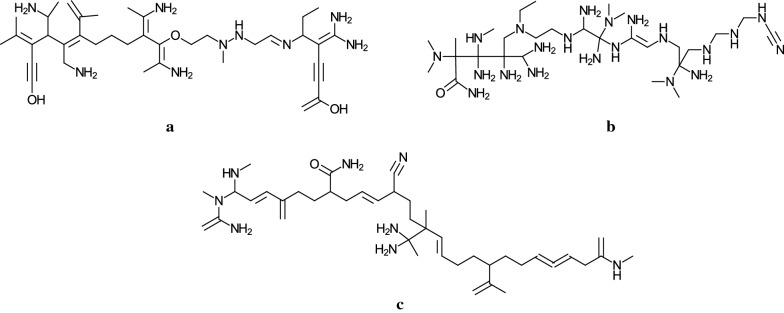
An illustration of chemical acyclic graphs *G* with $n(G)=50$, ${\text  {bl}}_2(G)=2$ and $d_\text  {max}=4$ obtained in Stage 4 by solving an MILP: **a** $y^*_{\text {Kow}}=9$, $\text  {dia}(G)=\lceil (2/5)n^*\rceil =20$; **b** $y^*_{\text {Bp}}=880$, $\text  {dia}(G)= n^*/2 =25$; **c** $y^*_{\text {Hc}}=25000$, $\text  {dia}(G)=\lceil (3/5)n^*\rceil =30$

**Fig. 8:**
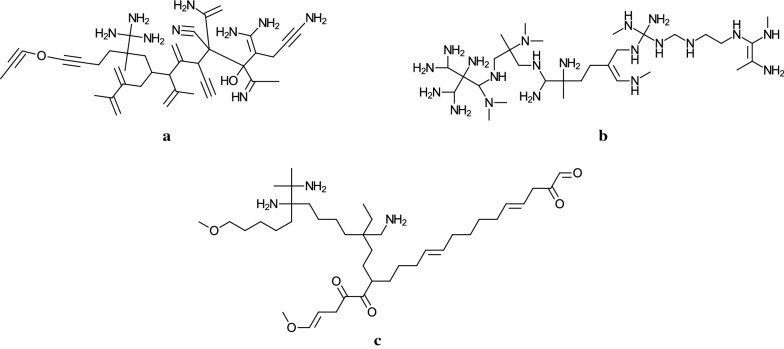
An illustration of chemical acyclic graphs *G* with $n(G)=50$, ${\text  {bl}}_2(G)=3$ and $d_\text  {max}=4$ obtained in Stage 4 by solving an MILP: **a** $y^*_{\text {Kow}}=9$, $\text  {dia}(G)=\lceil (2/5)n^*\rceil =20$; **b** $y^*_{\text {Bp}}=880$, $\text  {dia}(G)= n^*/2 =25$; **c** $y^*_{\text {Hc}}=25,000$, $\text  {dia}(G)=\lceil (3/5)n^*\rceil =30$

Figure 7**a**–**c** illustrate some chemical acyclic graphs *G* with ${\text  {bl}}_2(G)=2$ obtained in Stage 4 by solving an MILP. Remember that these chemical graphs obey the AD ${\mathcal {D}}$ defined in Appendix A.

Figure 8**a**–**c** illustrate some chemical acyclic graphs *G* with ${\text  {bl}}_2(G)=3$ obtained in Stage 4 by solving an MILP.

Stage 5. In this stage, we execute our new graph search algorithms for generating target graphs $G\in {\mathcal {G}}(\pmb {x}^*)$ with ${\text  {bl}}_2(G)\in \{2,3\}$ for a given feature vector $\pmb {x}^*$ obtained in Stage 4.

We introduce a time limit of 10 minutes for each iteration *h* in Step 2 and an execution of Steps 1 and 3 for ${\text  {bl}}^*=2$ (resp., each iteration *h* in Steps 2 and 3 and $\delta _1$ in Step 4 and an execution of Steps 1 and 5 for ${\text  {bl}}^*=3$). In the last step, we choose at most 100 feasible vector pairs and generate a target graph from each of these feasible vector pairs. We also impose an upper bound $\text  {UB}$ on the size $|\text  {W}|$ of a vector set $\text  {W}$ that we maintain during an execution of the algorithm. We executed the algorithm for each of the three bounds $\text  {UB}=10^6, 10^7, 10^8$ until a feasible vector pair is found or the running time exceeds a global time limitation of two hours.

When no feasible vector pair is found by the graph search algorithms, we output the target graph $G^*$ constructed from the vector $g^*$ in Stage 4.

Tables 6, 7 (resp., Tables 8, 9) show the results of Stage 5 for ${\text  {bl}}^*=2$ (resp., ${\text  {bl}}^*=3$), where we denote the following:

- $\#$FP: the number of feasible vector pairs obtained by an execution of the graph search algorithm for a given feature vector $\pmb {x}^*$;
- G-LB: a lower bound on the number of all target graphs $G\in {\mathcal {G}}(\pmb {x}^*)$ for a given feature vector $\pmb {x}^*$;
- $\#$G: the number of all (or up to 100) chemical acyclic graphs *G* such that $f(G)=x^*$ (where at least one such graph *G* has been found from the vector $g^*$ in Stage 4);
- G-time: the running time (sec.) to execute Stage 5 for a given feature vector $\pmb {x}^*$, where “> 2 hours” means that the running time exceeds two hours.

Previously, an instance of chemical acyclic graphs with size $n^*$ up to 16 was solved in Stage 5 by Azam et al. [17]. For the classes of chemical graphs with cycle index 1 and 2, the maximum size of instances solved in Stage 5 by Ito et al. [17] and Zhu et al. [21] was around 18 and 15, respectively. Our new algorithm based on dynamic programming solves instances with $n^*=50$. In our experiments, we also computed a lower bound G-LB on the number of target graphs. We observe that there are over $10^{10}$ or $10^{14}$ target graphs in some cases. Remember that these lower bounds are computed without actually generating each target graph one by one. So when a lower bound is enormously large, this would suggest that we may need to impose some more constraints on the structure of graphs or the range of descriptors to narrow a family of target graphs to be inferred.

**Table 6:** Results of Stages 4 and 5 for ${\text  {bl}}^*= 2$, $d_\text  {max}=3$ and $\text  {dia}^* = \lceil \frac{2}{5}n^* \rceil$

$\pi$	$y^*$	$n^*$	$\text {dia}^*$	IP-time	$\#$FP	G-LB	$\#$G	G-time
Kow	4	26	11	3.95	11,780	$2.4 \times 10^{6}$	100	0.91
	5	32	13	4.81	216	$2.7 \times 10^{4}$	100	10.64
	7	38	16	7.27	19,931	$4.2 \times 10^{7}$	100	48.29
	8	44	18	9.33	241,956	$1.2 \times 10^{13}$	100	119.01
	9	50	20	21.57	58,365	$1.7 \times 10^{10}$	100	110.38
Bp	440	26	11	2.09	22,342	$3.6 \times 10^{7}$	100	2.9
	550	32	13	3.94	748	$5.9 \times 10^{6}$	100	3.77
	660	38	16	6.4	39,228	$7.3 \times 10^{8}$	100	151.25
	770	44	18	7.21	138,076	$3.0 \times 10^{12}$	100	182.66
	880	50	20	9.49	106,394	$3.0 \times 10^{10}$	100	217.18
Hc	13000	26	11	2.94	12	$2.0 \times 10^{1}$	12	0.04
	16500	32	13	7.67	2722	$1.2 \times 10^{7}$	100	0.31
	20000	38	16	10.5	1830	$9.7 \times 10^{5}$	100	1.06
	23000	44	18	13.62	12,336	$4.7 \times 10^{8}$	100	142.02
	25000	50	20	15.1	136,702	$5.3 \times 10^{14}$	100	22.26

**Table 7:** Results of Stages 4 and 5 for ${\text  {bl}}^*= 2$, $d_\text  {max}=4$ and $\text  {dia}^* = \lceil \frac{3}{5}n^* \rceil$

$\pi$	$y^*$	$n^*$	$\text {dia}^*$	IP-time	$\#$FP	G-LB	$\#$G	G-time
Kow	4	26	16	16.21	4198	$3.5 \times 10^{5}$	100	1.18
	5	32	20	24.74	1650	$5.3 \times 10^{6}$	100	0.69
	7	38	23	38.88	154,408	$9.5 \times 10^{9}$	100	67.31
	8	44	27	38.73	1,122,126	$8.5 \times 10^{13}$	100	660.37
	9	50	30	31.59	690,814	$1.1 \times 10^{15}$	100	238.02
Bp	440	26	16	12.44	8156	$2.6 \times 10^{6}$	100	2.74
	550	32	20	23.22	38,600	$4.4 \times 10^{8}$	100	12.72
	660	38	23	20.62	52,406	$1.1 \times 10^{9}$	100	197.89
	770	44	27	50.55	23,638	$6.8 \times 10^{8}$	100	244.56
	880	50	30	48.37	40,382	$2.2 \times 10^{11}$	100	884.99
Hc	13000	26	16	23.26	249	$2.7 \times 10^{3}$	100	0.06
	16500	32	20	44.2	448	$6.9 \times 10^{4}$	100	0.63
	20000	38	23	96.02	3330	$6.1 \times 10^{6}$	100	15.16
	23000	44	27	82.34	43,686	$1.5 \times 10^{10}$	100	152.96
	25000	50	30	83.81	311,166	$1.3 \times 10^{13}$	100	287.95

**Table 8:** Results of Stages 4 and 5 for ${\text  {bl}}^*= 3$, $d_\text  {max}=3$ and $\text  {dia}^* = \lceil \frac{2}{5}n^* \rceil$

$\pi$	$y^*$	$n^*$	$\text {dia}^*$	IP-time	$\#$FP	G-LB	$\#$G	G-time
Kow	4	26	11	3.1	511	$3.6 \times 10^{3}$	100	14.31
	5	32	13	4.72	3510	$6.8 \times 10^{6}$	100	851.21
	7	38	16	5.82	11,648	$1.2 \times 10^{8}$	100	612.86
	8	44	18	9.69	17,239	$2.2 \times 10^{8}$	100	703.92
	9	50	20	22.53	60,792	$3.9 \times 10^{12}$	100	762.17
Bp	440	26	11	3.01	66	$9.0 \times 10^{2}$	66	902.77
	550	32	13	4.29	308	$1.0 \times 10^{7}$	100	2238.62
	660	38	16	5.86	303	$1.8 \times 10^{7}$	100	3061.11
	770	44	18	14.39	19,952	$4.7 \times 10^{10}$	100	678.26
	880	50	20	10.39	17,993	$7.1 \times 10^{12}$	100	4151.07
Hc	13000	26	11	3.05	340	$1.5 \times 10^{4}$	100	1.57
	16500	32	13	5.81	600	$3.1 \times 10^{8}$	100	921.55
	20000	38	16	15.67	18,502	$6.2 \times 10^{8}$	100	1212.54
	23000	44	18	21.15	5064	$6.9 \times 10^{9}$	100	1279.95
	25000	50	20	31.90	41,291	$2.4 \times 10^{12}$	100	668.5

**Table 9:** Results of Stages 4 and 5 for ${\text  {bl}}^*= 3$, $d_\text  {max}=4$ and $\text  {dia}^* = \lceil \frac{3}{5}n^* \rceil$

$\pi$	$y^*$	$n^*$	$\text {dia}^*$	IP-time	$\#$FP	G-LB	$\#$G	G-time
Kow	4	26	16	9.94	100	$2.5 \times 10^{4}$	100	6.73
	5	32	20	16.58	348	$1.4 \times 10^{8}$	100	3400.74
	7	38	23	33.71	17,557	$1.2 \times 10^{11}$	100	2652.38
	8	44	27	34.28	0	0	1	>2 hours
	9	50	30	68.74	80,411	$6.4 \times 10^{15}$	100	6423.85
Bp	440	26	16	14.16	150	$1.8 \times 10^{5}$	100	29.72
	550	32	20	18.94	305	$1.4 \times 10^{7}$	100	2641.9
	660	38	23	21.15	1155	$2.0 \times 10^{9}$	100	4521.66
	770	44	27	25.6	1620	$4.3 \times 10^{8}$	100	175.2
	880	50	30	63.22	0	0	1	>2 hours
Hc	13000	26	16	31.87	12	$2.7 \times 10^{4}$	12	0.66
	16500	32	20	41.03	392	$3.4 \times 10^{8}$	100	2480.34
	20000	38	23	48.48	630	$1.4 \times 10^{5}$	100	105.59
	23000	44	27	143.75	341	$7.8 \times 10^{8}$	100	5269.1
	25000	50	30	315.91	10,195	$3.8 \times 10^{9}$	100	5697.08

**Fig. 9:**
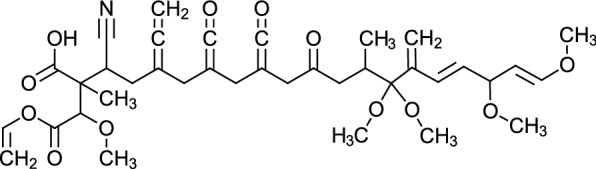
An illustration of a chemical acyclic graph *G* inferred for three chemical properties Kow, Bp and Hc simultaneously, where $y^*_{\text {Kow}} =4.0$, $y^*_{\text {Bp}} =400.0$ and $y^*_{\text {Hc}} =13000.0$, $n^* =50$, ${\text  {bl}}^* =2$, $\text  {dia}^* = 25$, and $d_\text  {max}=4$

*An additional experiment* We also conducted some additional experiment to demonstrate that our MILP-based method is flexible to control conditions on inference of chemical graphs. In Stage 3, we constructed an ANN ${{\mathcal {N}}}_{\pi }$ for each of the three chemical properties $\pi \in \{$ Kow, Bp, Hc$\}$, and formulated the inverse problem of each ANN ${{\mathcal {N}}}_{\pi }$ as an MILP ${{\mathcal {M}}}_{\pi }$. Since the set of descriptors is common to all three properties Kow, Bp and Hc, it is possible to infer a chemical acyclic graph *G* that satisfies a target value $y^*_{\pi }$ for each of the three properties at the same time (if one exists). We specify the size of graph so that $n^* =50$, ${\text  {bl}}^* =2$, $\text  {dia}^* = 25$ and $d_\text  {max}=4$, and set target values with $y^*_{\text {Kow}} =4.0$, $y^*_{\text {Bp}} =400.0$ and $y^*_{\text {Hc}} =13000.0$ in an MILP that consists of the three MILP ${{\mathcal {M}}}_{\text {Kow}}$, ${{\mathcal {M}}}_{\text {Hc}}$ and ${{\mathcal {M}}}_{\text {Bp}}$. The MILP was solved in 18930 seconds and we obtained a chemical acyclic graph *G* illustrated in Fig. 9. We continued to execute Stage 5 for this instance to generate more target graphs $G^*$. Table 10 shows that 100 target graphs are generated by our new dynamic programming algorithm.

**Table 10:** Results of Stages 4 and 5 for ${\text  {bl}}^*= 2$, $d_\text  {max}=4$, $n^* = 50$ and $\text  {dia}^* = 25$

$\pi$	$y^*$	$n^*$	$\text {dia}^*$	IP-time	$\#$FP	G-LB	$\#$G	G-time
Kow	4	50	25	18930.46	117,548	$2.4 \times 10^{11}$	100	423.53
Bp	400							
Hc	1300							

## Concluding remarks

In this paper, we introduced a new measure, branch-height of a tree, and showed that many chemical compounds in the chemical database have a simple structure where the number of 2-branches is small. Based on this, we proposed a new method of applying the framework for inverse QSAR/QSPR [17–19] to the case of acyclic chemical graphs where Azam et al. [17] inferred chemical graphs with around 20 non-hydrogen atoms and Zhang et al. [19] solved an MILP of inferring a feature vector for an instance with diameter 9. In our method, we formulated a new MILP in Stage 4 specialized for acyclic chemical graphs with a small branch number and designed a new graph search algorithm in Stage 5 that computes frequency vectors of graphs in a dynamic programming scheme.

We implemented our new method and conducted some experiments on chemical properties such as octanol/water partition coefficient, boiling point and heat of combustion.

The resulting method improved the performance so that chemical graphs with around 50 non-hydrogen atoms and around diameter 30 can be inferred. Since there are many acyclic chemical compounds having large diameters, this is a significant improvement.

It is left as a future work to design MILPs and graph search algorithms based on the new idea of the paper for classes of graphs with a higher rank. Recently, a method for inferring a chemical cyclic graph with any rank has been designed by Akutsu and Nagamochi [27] based on the ideas in this paper. The method is also designed so that a target chemical graph to be inferred can be specified in a more flexible way, where we can include a prescribed substructure of graphs such as a benzene ring into a target chemical graph while imposing constraints on a global topological structure of a target graph at the same time.

## Figures and Tables

**Fig. 10:**
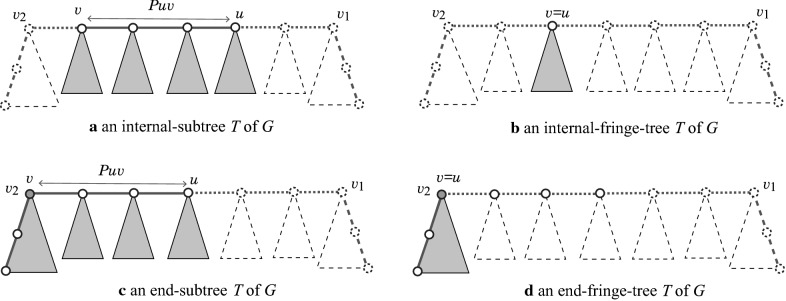
An illustration of subtrees *T* of a chemical acyclic graph *G* in Fig. 6a, where the vertices/edges in *T* are depicted by solid lines: **a** An internal-subtree *T* of *G*; **b** An internal-fringe-tree *T* of *G*; **c** An end-subtree *T* of *G*; **d** An end-fringe-tree *T* of *G*

**Fig. 11:**
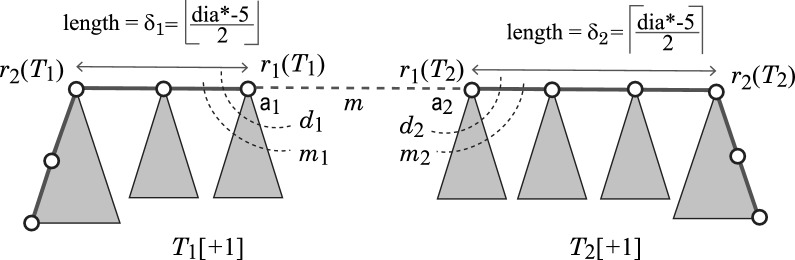
An illustration of combining two bi-rooted trees $T_1=T_{\pmb {w}^1}$ and $T_2=T_{\pmb {w}^2}$ with a new edge with multiplicity *m* joining vertices $r_1(T_1)$ and $r_1(T_2)$ to construct a target graph *G*, where ${\mathtt{a}}_i\in \Lambda$, $d_i\in [1,d_\text  {max}-1]$, $m_i\in [d_i,{\text  {val}}({\mathtt{a}}_i)-1]$, $i = 1, 2$, and $m \in [1, \min \{3, {\text  {val}}({\mathtt{a}}_1) - m_1, {\text  {val}}({\mathtt{a}}_2) - m_2\} ]$

**Fig. 12:**
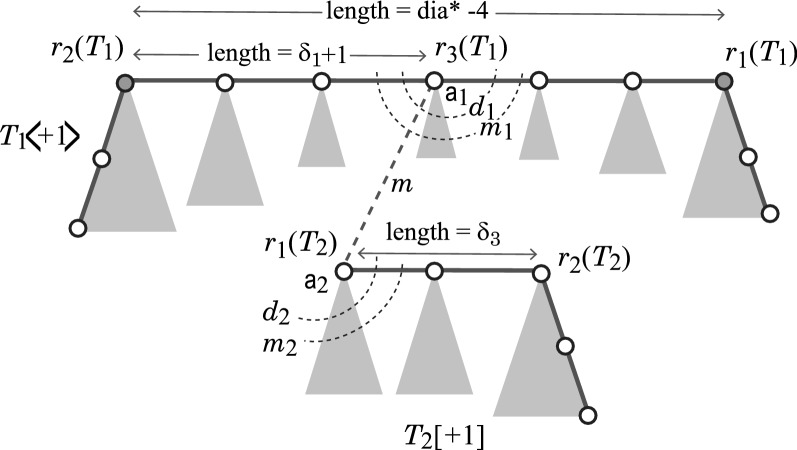
An illustration of combining a tri-rooted $T_1=T_{\pmb {w}^1}$ and a bi-rooted tree $T_2=T_{\pmb {w}^2}$ with a new edge joining vertices $r_3(T_1)$ and $r_1(T_2)$ to construct a target graph *G*

**Fig. 13:**
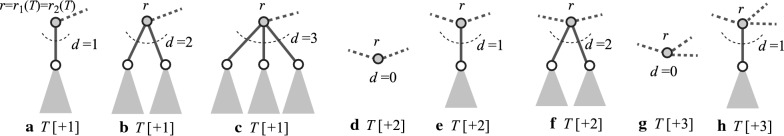
An illustration of fictitious rooted trees $T[+p]$, $p\in [1,3]$ for rooted trees *T* with $r=r_1(T)=r_2(T)$ and $d=\deg _T(r)$, where a dashed line depicts a fictitious edge incident to the terminal $r_1(T)=r_2(T)$: (**a**) $T[+1]$ and $d=1$; (**b**) $T[+1]$ and $d=2$; (**c**) $T[+1]$ and $d=3$; (**d**) $T[+2]$ and $d=0$; (**e**) $T[+2]$ and $d=1$; (**f**) $T[+2]$ and $d=2$; (**g**) $T[+3]$ and $d=0$; (**h**) $T[+3]$ and $d=1$

**Fig. 14:**
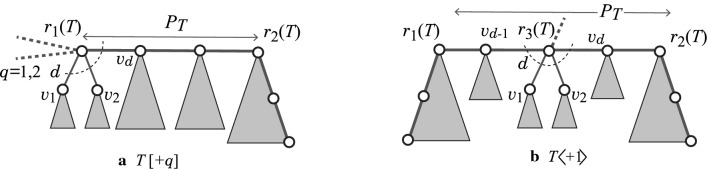
An illustration of fictitious trees $T[+q]$ and $T\langle +1\rangle$ for bi-rooted trees and tri-rooted trees *T*: **a** $T[+q]$ of a bi-rooted tree *T*; **b** $T\langle +1\rangle$ of a tri-rooted tree *T*

**Fig. 15:**
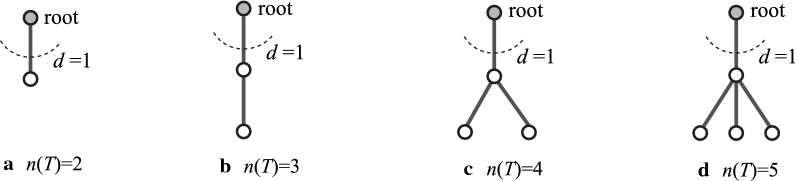
An illustration of rooted trees *T* with height at most 2 and only one child satisfying the size constraint: **a** case of $n(T)=2$; **b** case of $n(T)=3$; **c** case of $n(T)=4$; **d** case of $n(T)=5$

**Fig. 16:**
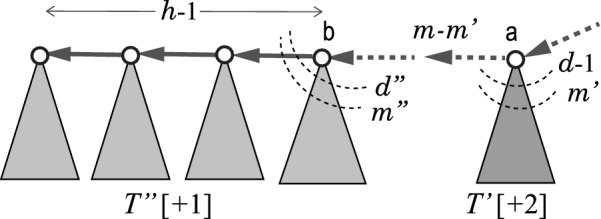
An illustration of appending a rooted tree $T'$ to a bi-rooted tree $T''$ to compute a vector $\pmb {w}\in \text  {W}_{\text  {end}}^{(h)}({\mathtt{a}}, d , m )$ from the frequency vectors $\pmb {w}'=\pmb {f}(T'[+2])\in \text  {W}_{\text  {inl}}^{(0)}({\mathtt{a}}, d -1, m')$ of a rooted tree $T'$ and $\pmb {w}'' =\pmb {f}(T''[+1]) \in \text  {W}_{\text  {end}}^{(h-1)}(\mathtt{b}, d'', m'')$ of a bi-rooted tree $T''$

**Fig. 17:**
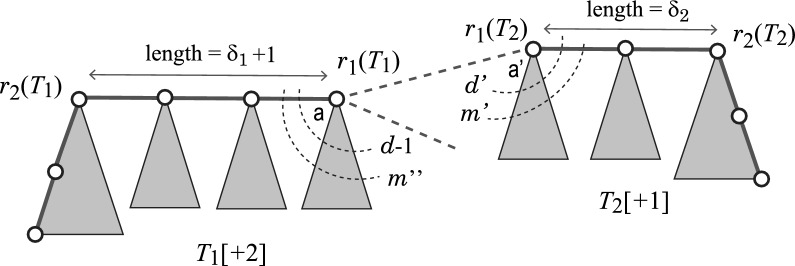
An illustration of computing the frequency vector $\pmb {w}=\pmb {f}(T\langle +1\rangle ) \in \text  {W}_{\text  {main}}^{(\delta _1+1)}({\mathtt{a}}, d, m)$ of a tri-rooted tree *T* from the frequency vectors $\pmb {w}^1=\pmb {f}(T_1[+2])\in \text  {W}_{\text  {end}+2}^{(\delta _1+1)}({\mathtt{a}}, d-1, m'')$ and $\pmb {w}^2=\pmb {f}(T_2[+1])\in \text  {W}_{\text  {end}}^{(\delta _2)}({\mathtt{a}}', d', m')$ for bi-rooted trees $T_1$ and $T_2$

## Data Availability

Source code of the implementation of our algorithm is freely available from https://github.com/ku-dml/mol-infer.

## Appendix A: Statistical features of molecular structures

We observe the following features of the graph-theoretical structure of chemical graphs registered in the chemical database PubChem. Let $\text  {DB}^{(\le n)}$ denote the set of chemical graphs with at most *n* non-hydrogen atoms that are registered in chemical database PubChem (downloaded a copy on March 21, 2019). The *cycle index* (or *rank*) of a chemical graph $G=(H=(V,E),\alpha ,\beta )$ is defined to be $|E|-(|V|-1)$ (i.e., the minimum number of edges to be removed to make the graph *H* acyclic). We call a chemical graph a *rank-r* chemical graph if the rank of the graph is *r*. The *core* of a chemical cyclic graph *G* is defined to be the induced subgraph $G'$ of *G* such that $G'$ consists of vertices in a cycle or vertices in a path joining two cycles. A vertex in the core (not in the core) is called a *core vertex* (resp., a non-core vertex). The edges not in the core of a chemical cyclic graph *G* form a collection of trees *T*, which we call a *non-core tree*. Each non-core tree contains exactly one core vertex and is regarded as a tree rooted at the core vertex. The *k*-*branch height* of a chemical cyclic graph *G* is defined to be the maximum of *k*-branch heights over all non-core trees.

Let $\rho _r$ (%) denote the ratio of the number of chemical graphs with rank at most $r\in [0,4]$ to the number of all chemical graphs in PubChem. See Table 11.

**Table 11:** The percentage $\rho _r$ of the number of chemical compounds with rank at most $r\in [0,4]$ over all chemical compounds in PubChem

$\rho _0$	$\rho _1$	$\rho _2$	$\rho _3$	$\rho _4$
$2.9\%$	$16.3\%$	$44.5\%$	$68.8\%$	$84.7\%$

Let $\rho _0^{(d)}$ (%) denote the ratio of the number of chemical graphs in $\text  {DB}^{(\le 100)}$ such that the maximum degree is at most $d\in [3,4]$ to the number of all chemical graphs in $\text  {DB}^{(\le 100)}$. Let $\rho _r^{(d)}$ (%), $r\in [1,4]$ denote the ratio of the number of rank-*r* chemical graphs in $\text  {DB}^{(\le 100)}$ such that the maximum degree of a non-core vertex is at most $d\in [3,4]$ to the number of all rank-*r* chemical graphs in $\text  {DB}^{(\le 100)}$. See Table 12.

**Table 12:** The percentage $\rho _r^{(d)}$ of the number of chemical compounds with rank $r\in [0,4]$ such that the maximum degree of a non-core vertex is at most $d\in [3,4]$ over all rank-*r* chemical compounds in $\text  {DB}^{(\le 100)}$

$\rho _0^{(3)}$	$\rho _0^{(4)}$	$\rho _1^{(3)}$	$\rho _1^{(4)}$	$\rho _2^{(3)}$	$\rho _2^{(4)}$	$\rho _3^{(3)}$	$\rho _3^{(4)}$	$\rho _4^{(3)}$	$\rho _4^{(4)}$
$55.55\%$	$99.85\%$	$68.30\%$	$99.97\%$	$84.46\%$	$99.99\%$	$87.11\%$	$99.99\%$	$87.75\%$	$99.99\%$

Let $\rho _r(k,h)$ (%), $r\in [0,4]$, $k=2$, $h\in [1,2]$ denote the ratio of the number of rank-*r* chemical graphs in $\text  {DB}^{(\le 50)}$ such that the *k*-branch height is at most *h* to the number of all rank-*r* chemical graphs in $\text  {DB}^{(\le 50)}$. See Table 13. We see that most chemical graphs *G* with at most 50 non-hydrogen atoms satisfy $\text  {bh}_2(G)\le 2$.

**Table 13:** The percentage $\rho _r(k,h)$ (%) of the number of rank-*r* chemical graphs in $\text  {DB}^{(\le 50)}$ such that the *k*-branch height is at most *h* to the number of all rank-*r* chemical graphs in $\text  {DB}^{(\le 50)}$

$\rho _0(2,1)$	$\rho _0(2,2)$	$\rho _1(2,1)$	$\rho _1(2,2)$	$\rho _2(2,1)$	$\rho _3(2,1)$	$\rho _4(2,1)$
$87.23\%$	$99.46\%$	$88.13\%$	$98.76 \%$	$96.39\%$	$99.17\%$	$99.43\%$

We show the distribution of 2-branch height over alkans C$_n$H$_{2n+2}$. Let $\text  {Aln}(n)$ denote the set of all alkans with *n* carbon atoms, where $|\text  {Aln}(25)|=36,797,588$. Let $\rho _{\text  {Aln}}(2,h)$ (%), $h\in [1,4]$ denote the ratio of the number of alkans in $\text  {Aln}(25)$ such that the 2-branch height is at most *h* to the number of alkans in $\text  {Aln}(25)$. See Table 14.

**Table 14:** The percentage $\rho _{\text  {Aln}}(2,h)$ (%) of the number of alkans in $\text  {Aln}(25)$ such that the 2-branch height is at most *h* to the number of alkans in $\text  {Aln}(25)$

$\rho _{\text {Aln}}(2,1)$	$\rho _{\text {Aln}}(2,2)$	$\rho _{\text {Aln}}(2,3)$	$\rho _{\text {Aln}}(2,4)$
$49.03\%$	$97.67\%$	$99.99\%$	$100.00\%$

Let $\rho _{\text  {2bt}}(\delta )$ denote the ratio of the number of acyclic chemical graphs in $\text  {DB}^{(\le 50)}$ such that the degree of the root of the 2-branch-tree is $\delta \in [1,4]$ to the number of all acyclic chemical graphs in $\text  {DB}^{(\le 50)}$. See Table 15.

**Table 15:** The percentage $\rho _{\text  {2bt}}(\delta )$ of the number of acyclic chemical graphs in $\text  {DB}^{(\le 50)}$ such that the degree of the root of the 2-branch-tree is $\delta \in [1,4]$ to the number of all acyclic chemical graphs in $\text  {DB}^{(\le 50)}$

$\rho _{\text {2bt}}(1)$	$\rho _{\text {2bt}}(2)$	$\rho _{\text {2bt}}(3)$	$\rho _{\text {2bt}}(4)$
$6.39\%$	$83.58\%$	$9.30\%$	$0.73\%$

Among the 2-fringe-trees *T* of all acyclic chemical graphs in $\text  {DB}^{(\le 100)}$, over $90\%$ of them satisfy $n\le 2d+2$ for the number $n=|V(T)|$ of non-hydrogen atoms in a 2-fringe-tree *T* and the number *d* of non-hydrogen atoms adjacent to the root in *T*.

Let ${\mathcal {FT}}_{0,2}$ denote the set of all 2-fringe-trees that appear in an acyclic chemical graph in $\text  {DB}^{(\le 100)}$, and ${\mathcal {FT}}_{0,2}^{(\delta )}$, $\delta \in [1,3]$ denote the set of all 2-fringe-trees $T\in {\mathcal {FT}}_{0,2}$ that have $\delta$ children (i.e., the degree of the root is $\delta$). Let $\rho _{2\delta +2}^{(\delta )}$ (%) denote the ratio of the number of 2-fringe-trees in ${\mathcal {FT}}_{0,2}^{(\delta )}$ that have at most $2\delta +2$ vertices to the number of 2-fringe-trees in ${\mathcal {FT}}_{0,2}^{(\delta )}$. See Table 16.

**Table 16:** The percentage $\rho _{2\delta +2}^{(\delta )}$ (%) of the number of 2-fringe-trees in ${\mathcal {FT}}_{0,2}^{(\delta )}$ that have at most $2\delta +2$ vertices to the number of 2-fringe-trees in ${\mathcal {FT}}_{0,2}^{(\delta )}$

$\rho _{4}^{(1)}$	$\rho _{6}^{(2)}$	$\rho _{8}^{(3)}$
$93.77\%$	$93.99\%$	$92.01\%$

## Appendix B: Formulating an MILP based on scheme graphs

This section shows how to formulate an MILP based on a scheme graph.

### Scheme graphs

Let $t^*$, $s^*$, and $c^*$, be integers such that

- $$t^*= n^* - (\text  {bh}^* -1) - (k^*+1){\text  {bl}}^*$$;
- $s^*=a(b^c-1)/(b-1)+1$ for $a=d_\text  {max}$, $b=d_\text  {max}-1$ and $c=\text  {bh}^*$; and
- $$c^*=s^*-1$$.

Let a scheme graph $\text  {SG}( d_\text  {max}, k^*, \text  {bh}^*, t^*)$ consist of a tree $T_B$, a path $P_{t^*}$, a set $\{S_s\mid s\in [1,s^*]\}$ of trees, a set $\{T_t\mid t\in [1,t^*]\}$ of trees, and a set of directed edges between $T_B$ and $P_{t^*}$ so that an acyclic graph $H\in {\mathcal {H}}(n^*, d_\text  {max}, \text  {dia}^*, k^*, \text  {bh}^*, {\text  {bl}}^*)$ will be constructed in the following way:

(i) The $k^*$-branch-tree of *H* will be chosen as a subtree of $T_B=(V_B,E_B)$;
(ii) Each $k^*$-fringe-tree rooted at a vertex $u_s\in V(T_B)$ of *H* will be chosen as a subtree of $S_s$;
(iii) Each $k^*$-branch-path of *H* (except for its end-vertices) will be chosen as a subpath of $P_{t^*}$ or as an edge in $T_B$;
(iv) Each $k^*$-fringe-tree rooted at a vertex $v_t\in V(P_{t^*})$ of *H* will be chosen as a subtree of $T_t$; and
(v) An edge (*u*, *v*) directed from $T_B$ to $P_{t^*}$ will be selected as an initial edge of a $k^*$-branch-path of *H* and an edge (*v*, *u*) directed from $P_{t^*}$ to $T_B$ will be selected as an ending edge of a $k^*$-branch-path of *H*.

More formally, each component of a scheme graph $\text  {SG}( d_\text  {max}, k^*, \text  {bh}^*, t^*)$ is defined as follows.

(i) $$T_B =(V_B=\{u_1,u_2,\ldots ,u_{s^*}\},$$$$E_B=\{a_1,a_2,\ldots ,a_{c^*}\})$$, called a *base-tree* is a tree rooted at a vertex $$u_1$$ that is isomorphic to the rooted tree $$T(d_\text  {max}, d_\text  {max}- 1, \text  {bh}^*)$$. Regard $$T_B$$ as an ordered tree by introducing a total order for each set of siblings and call the first (resp., last) child in a set of siblings the leftmost (resp. rightmost) child, which defines the leftmost (rightmost) path from the root $$u_1$$ to a leaf in $$T_B$$, as illustrated in Fig. 4a.
  For each vertex $u_s\in V_B$, let $E_B(s)$ denote the set of indices *i* of edges $a(i)\in E_B$ incident to $u_s$ and $\text  {Cld}_B(s)$ denote the set of indices *i* of children $u_i\in V_B$ of $u_s$ in the tree $T_B$.
  For each integer $d\in [0,k^*]$, let $V_B(d)$ denote the set of indices *s* of vertices $u_s\in V_B$ whose depth is *d* in the tree $T_B$, where $V_B(\text  {bh}^*)$ is the set of indices *s* of leaves $u_s$ of $T_B$.
  Regard each edge $a_i\in E_B$ as a directed edge $(u_s,u_{s'})$ from one end-vertex $u_s$ of $a_i$ to the other end-vertex $u_{s'}$ of $a_i$ such that $s=\text  {prt}(s')$ (i.e., $u_s$ is the parent of $u_{s'}$), where $\text  {head}(i)$ and $\text  {tail}(i)$ denote the head $u_{s'}$ and tail $u_s$ of edge $a_i\in E_B$, respectively.
  For each index $s\in [1,s^*]$, let $E_B^+(s)$ (resp., $E_B^-(s)$) denote the set of indices *i* of edges $a_i\in E_B$ such that the tail (resp., head) of $a_i$ is vertex $u_{s}$.
  Let $L_B$ denote the set of indices of leaves of $T_B$, and $s^\text  {left}$ (resp., $s^\text  {right}$) denote the index $s\in L_B$ of the leaf $u_s$ at which the leftmost (resp., rightmost) path from the root ends.
  For each leaf $u_s$, $s\in L_B$, let $V_{B,s}$ (resp., $E_{B,s}$) denote the set of indices *s* of non-root vertices $u_s$ (resp., indices *i* of edges $a(i)\in E_B$) along the path from the root to the leaf $u_s$ in the tree $T_B$.
  For the example of a base-tree $T_B$ with $\text  {bh}^*=2$ in Fig. 4, it holds that $L_B=\{5,6,7,8,9,10\}$, $s^\text  {left}=5$, $s^\text  {right}=10$, $E_{B,s^\text  {left}}=\{1,4\}$ and $V_{B,s^\text  {left}}=\{2,5\}$.
(ii) $S_s$, $s\in [1,s^*]$ is a tree rooted at vertex $u_s\in V_B$ in $T_B$ that is isomorphic to the rooted tree $T(d_\text  {max}-1, d_\text  {max}-1, k^*)$, as illustrated in Fig. 4b. Let $u_{s,i}$ and $e'_{s,i}$ denote the vertex and edge in $S_s$ that correspond to the *i*-th vertex and the *i*-th edge in $T(d_\text  {max}-1, d_\text  {max}-1, k^*)$, respectively. Regard each edge $e'_{s,i}$ as a directed edge $(u_{s,\text  {prt}(i)},u_{s,i})$. For this, each vertex $u_s\in V_B$ is also denoted by $u_{s,1}$.
(iii) $P_{t^*}=(V_P=\{v_1$, $v_2$, $\ldots$, $v_{t^*}\}$, $E_P=\{e_2$, $e_3$, $\ldots$, $e_{t^*}\})$, called a *link-path* with size $t^*$ is a directed path from vertex $v_1$ to vertex $v_{t^*}$, as illustrated in Fig. 4a. Each edge $e_t\in E_P$ is directed from vertex $v_{t-1}$ to vertex $v_t$.
(iv) $T_t$, $t\in [1,t^*]$ is a tree rooted at vertex $v_t$ in $P_{t^*}$ that is isomorphic to the rooted tree $T(d_\text  {max}-2, d_\text  {max}-1, k^*)$, as illustrated in Fig. 4c. Let $v_{t,i}$ and $e_{t,i}$ denote the vertex and edge in $T_t$ that correspond to the *i*-th vertex and the *i*-th edge in $T(d_\text  {max}-2, d_\text  {max}-1, k^*)$, respectively. Regard each edge $e_{t,i}$ as a directed edge $(v_{t,\text  {prt}(i)},u_{t,i})$. For this, each vertex $v_t\in V_P$ is also denoted by $v_{t,1}$.
(v) For every pair (*s*, *t*) with $s\in [1,s^*]$ and $t\in [1,t^*]$, join vertices $u_{s}$ and $v_{t}$ with directed edges $(u_{s},v_{t})$ and $(v_{t},u_{s})$, as illustrated in Fig. 4a.

We explain the basic idea of an MILP in Theorem 2. The MILP mainly consists of the following three types of constraints.

1. Constraints for selecting an acyclic graph *H* as a subgraph of the scheme graph $\text  {SG}( d_\text  {max}, k^*, \text  {bh}^*, t^*);$
2. Constraints for assigning chemical elements to vertices and multiplicity to edges to determine a chemical graph $G=(H,\alpha ,\beta )$; and
3. Constraints for computing descriptors from the selected acyclic chemical graph *G*.

In the constraints of C1, more formally we prepare the following.

(i) In the scheme graph $\text  {SG}( d_\text  {max}, k^*, \text  {bh}^*, t^*)$, we prepare a binary variable *u*(*s*, 1) for each vertex $u_s=u_{s,1}\in V_B$, $s\in [1,s^*]$ so that vertex $u_s=u_{s,1}$ becomes a $k^*$-branch of a selected graph *H* if and only if $u(s,1)=1$. The subgraph of the base-tree $T_B$ that consists of vertices $u_s=u_{s,1}$ with $u(s,1)=1$ will be the $k^*$-branch-tree of the graph *H*. We also prepare a binary variable *a*(*i*), $i\in [1,c^*]$ for each edge $a_i\in E_B$, where $c^*=s^*-1$. For a pair of a vertex $u_{s,1}$ and a child $u_{s',1}$ of $u_{s,1}$ such that $u(s,1)=u(s',1)=1$, either the edge $a_i=(u_{s,1},u_{s',1})$ is used in the selected graph *H* (when $a(i)=1$) or a path $P_i=(u_{s,1},v_{t',1},v_{t'+1,1},\ldots ,v_{t'',1},u_{s',1})$ from vertex $u_{s,1}$ to vertex $u_{s',1}$ is constructed in *H* with an edge $(u_{s,1},v_{t',1})$, a subpath $(v_{t',1},v_{t'+1,1},\ldots ,v_{t'',1})$ of the link-path $P_{t^*}$ and an edge $(v_{t'',1},u_{s',1})$ (when $a(i)=0$). For example, vertices $u_{1,1}$ and $u_{2,1}$ are connected by a path $P_1=(u_{1,1},v_{1,1},v_{2,1},u_{2,1})$ in the selected graph $H'$ in Fig. 5c.
(ii) Let
  - $$n_\text  {tree}^{\text  {S}} =1+(d_\text  {max}-1)((d_\text  {max}-1)^{k^*}-1)/(d_\text  {max}-2),$$
  - $$n_\text  {tree}^{\text  {T}} =1+(d_\text  {max}-2)((d_\text  {max}-1)^{k^*}-1)/(d_\text  {max}-2),$$
  where $n_\text  {tree}^{\text  {S}}$ (resp., $n_\text  {tree}^{\text  {T}}$) is the number of vertices in the rooted tree $T(d_\text  {max}-1, d_\text  {max}-1, k^*)$ (resp., $T(d_\text  {max}-2, d_\text  {max}-1, k^*)$). In each tree $S_s$, $s\in [1,s^*]$ (resp., $T_t$, $t\in [1,t^*]$) in the scheme graph, we prepare a binary variable *u*(*s*, *i*) (resp., *v*(*t*, *i*)) for each vertex $u_{s,i}$, $i\in [2, n_\text  {tree}^{\text  {S}}]$ (resp., $v_{t,i}$, $i\in [2, n_\text  {tree}^{\text  {T}}]$) so that $u(s,i)=1$ (resp., $v(t,i)=1$) means that the corresponding vertex $u_{s,i}$ (resp., $v_{t,i}$) is used as a vertex in a selected graph *H*. The (non-empty) subgraph of a tree $S_s$ (resp., $T_t$) that consists of vertices $u_{s,i}$ with $u(s,i)=1$ (resp., $v_{t,i}$ with $v(t,i)=1$) will be a $k^*$-fringe-tree of a selected graph *H*.
(iii) In the link-path $P_{t^*}$, we prepare a binary variable *e*(*t*), $t\in [2,t^*]$ for each edge $e_{t,1}=(v_{t-1,1}, v_{t,1})\in E_P$ so that $e(t)=1$ if and only if edge $e_{t,1}$ is used in some path $P_i=(u_{s,1},v_{t',1},v_{t'+1,1},\ldots ,v_{t'',1},u_{s',1})$ constructed in (i).
(iv) For each pair (*s*, *t*) of $s\in [1,s^*]$ and $t\in [1,t^*]$, we prepare a binary variable *e*(*s*, *t*) (resp., *e*(*t*, *s*)) so that $e(s,t')=1$ (resp., $e(t'',s)=1$) if and only if directed edge $(u_{s,1},v_{t',1})$ (resp., $(v_{t'',1},u_{s,1})$) is used as the first edge (resp., last edge) of some path $P_i=(u_{s,1},v_{t',1},v_{t'+1,1},\ldots , v_{t'',1},u_{s',1})$ constructed in (i).

Based on these, we include constraints with some more additional variables so that a selected subgraph *H* is a connected acyclic graph. See constraints (12) to (32) in Appendix C for the details.

In the constraints of C2, we prepare an integer variable ${\widetilde{\alpha }}(u)$ for each vertex *u* in the scheme graph that represents the chemical element $\alpha (u)\in \Lambda$ if *u* is in a selected graph *H* (or ${\widetilde{\alpha }}(u)=0$ otherwise) and an integer variable ${\widetilde{\beta }}(e)\in [0,3]$ (resp., ${\widehat{\beta }}(e)\in [0,3]$) for each edge *e* (resp., $e=e(s,t)$ or *e*(*t*, *s*), $s\in [1,s^*]$, $t\in [1,t^*]$) in the scheme graph that represents the multiplicity $\beta (e)\in [1,3]$ if *e* is in a selected graph *H* (or ${\widetilde{\beta }}(e)$ or ${\widehat{\beta }}(e)$ takes 0 otherwise). This determines a chemical graph $G=(H,\alpha ,\beta )$. Also we include constraints for a selected chemical graph *G* to satisfy the valence condition $(\alpha (u),\alpha (v),\beta (uv))\in \Gamma$ for each edge $uv\in E$. See constraints (33) to (47) in Appendix C for the details.

In the constraints of C3, we introduce a variable for each descriptor and constraints with some more variables to compute the value of each descriptor in *f*(*G*) for a selected chemical graph *G*. See constraints (48) to (75) in Appendix C for the details.

## Appendix C: All constraints in an MILP formulation for chemical acyclic graphs

To formulate an MILP that represents a chemical graph, we distinguish a tuple $(\mathtt{a,b},m)$ from a tuple $(\mathtt{b,a},m)$. For a tuple $\gamma =(\mathtt{a,b},m)\in \Lambda \times \Lambda \times \{1,2,3\}$, let $\overline{\gamma }$ denote the tuple $(\mathtt{b, a}, m)$. Let $\Gamma _{<}\triangleq \{\overline{\gamma }\mid \gamma \in \Gamma _{>}\}$. We call a tuple $\gamma =(\mathtt{a, b}, m) \in \Lambda \times \Lambda \times \{1, 2, 3\}$ *proper* if $m \le \min \{ {\text  {val}}({\mathtt{a}}), {\text  {val}}(\mathtt{b})\}$ and $m \le \max \{ {\text  {val}}({\mathtt{a}}), {\text  {val}}(\mathtt{b}) \}-1$, where the latter is assumed because otherwise *G* must consist of two atoms of $\mathtt{a=b}$. Assume that each tuple $\gamma \in \Gamma$ is proper. Let $\epsilon$ be a fictitious chemical element that represents null, call a tuple $(\mathtt{a,b},0)$ with $\mathtt{a,b}\in \Lambda \cup \{\epsilon \}$ *fictitious*, and define $\Gamma _0$ to be the set of all fictitious tuples; i.e., $\Gamma _0=\{(\mathtt{a,b},0) \mid \mathtt{a,b}\in \Lambda \cup \{\epsilon \}\}$. To represent chemical elements $\mathtt{e}\in \Lambda \cup \{\epsilon \}\cup \Gamma$ in an MILP, we encode these elements $\mathtt{e}$ into some integers denoted by $[\mathtt{e}]$. Assume that, for each element ${\mathtt{a}}\in \Lambda$, $[{\mathtt{a}}]$ is a positive integer and that $[\epsilon ]=0$.

### Upper and lower bounds on descriptors

In our formulation of an MILP for inferring a vector $x^*$ in Stage 4, we fix the following descriptors as specified constants: the number *n*(*G*) of vertices, the diameter $\text  {dia}(G)$, and the number ${\text  {bl}}_{k^*}(G)$ of leaf $k^*$-leaf branches, which are set to be given integers $n^*$, $\text  {dia}^*$, and ${\text  {bl}}^*$, respectively. For each of the other descriptors, we specify a lower bound $\text  {LB}$ and an upper bound $\text  {UB}$ on the value so that the descriptor takes a value from the range between $\text  {LB}$ and $\text  {UB}$.

*constants*

- $n^*\ge 5$: the size *n*(*G*) of *G*;
- $$\text  {LB}_\text  {dg}^\text  {t}(i), \text  {UB}_\text  {dg}^\text  {t}(i)\in [0,n^*],$$$$i\in [1,4], \text  {t}\in \{{\text  {in}},\text  {ex}\}$$: lower and upper bounds on the number $$\text  {dg}_i^\text  {t}(G)$$ of $$k^*$$-internal/$$k^*$$-external vertices of degree *i* in *G*;
- $\text  {LB}_\text  {ce}^\text  {t}({\mathtt{a}}), \text  {UB}_\text  {ce}^\text  {t}({\mathtt{a}})\in [0,n^*]$, ${\mathtt{a}}\in \Lambda , \text  {t}\in \{{\text  {in}},\text  {ex}\}$: lower and upper bounds on the number $\text  {ce}_{\mathtt{a}}^\text  {t}(G)$ of $k^*$-internal/$k^*$-external vertices *v* with $\alpha (v)={\mathtt{a}}$ in *G*;
- $$\text  {LB}_\text  {bd}^\text  {t}(m)$$, $$\text  {UB}_\text  {bd}^\text  {t}(m)\in [0,n^*-1],$$$$m\in [2,3],$$$$\text  {t}\in \{{\text  {in}},\text  {ex}\}$$: lower and upper bounds on the number $$\text  {bd}_m^\text  {t}(G)$$ of $$k^*$$-internal/$$k^*$$-external edges *e* with $$\beta (e)=m$$ in *G*;
- $$\text  {LB}_\text  {ac}^\text  {t}(\gamma ),$$$$\text  {UB}_\text  {ac}^\text  {t}(\gamma )\in [0,n^*-1],$$$$\text  {t}\in \{{\text  {in}},\text  {ex}\},$$$$\gamma \in \Gamma _{<}\cup \Gamma _{=}$$: lower and upper bounds on the number $$ \text  {ac}_{\gamma }^\text  {t}(G)$$ of $$k^*$$-internal/$$k^*$$-external edges *e* with adjacency-configuration $$\gamma $$ in *G*;
- $\text  {LB}_\text  {bc}^\text  {t}(\mu ), \text  {UB}_\text  {bc}^\text  {t}(\mu )\in [0,n^*-1], \text  {t}\in \{{\text  {in}},\text  {ex}\}$, $\mu \in \text  {Bc}$: lower and upper bounds on the number $\text  {bc}_{\mu }^\text  {t}(G)$ of $k^*$-internal/$k^*$-external edges *e* with bond-configuration $\mu$ in *G*;

*variables**x**for descriptors *

- $\text  {dg}^{\text  {in}}(i), \text  {dg}^\text  {ex}(i)\in [0,n^*]$, $i\in [1,4]$: $\text  {dg}^{\text  {in}}(i)$ (resp., $\text  {dg}^\text  {ex}(i)$) represents $\text  {dg}_i^{\text  {in}}(G)$ (resp., $\text  {dg}_i^\text  {ex}(G)$);
- $\text  {ce}^{\text  {in}}({\mathtt{a}}), \text  {ce}^\text  {ex}({\mathtt{a}})\in [0,n^*]$, ${\mathtt{a}}\in \Lambda$: $\text  {ce}^{\text  {in}}({\mathtt{a}})$ (resp., $\text  {ce}^\text  {ex}({\mathtt{a}})$) represents $\text  {ce}_{\mathtt{a}}^{\text  {in}}(G)$ (resp., $\text  {ce}_{\mathtt{a}}^\text  {ex}(G)$);
- $\text  {bd}^{\text  {in}}(m), \text  {bd}^\text  {ex}(m)\in [0,2n^*]$, $m\in [1,3]$: $\text  {bd}^{\text  {in}}(m)$ (resp., $\text  {bd}^\text  {ex}(m)$) represents $\text  {bd}_m^{\text  {in}}(G)$ (resp., $\text  {bd}_m^\text  {ex}(G)$);
- $\text  {ac}^{\text  {in}}(\gamma ), \text  {ac}^\text  {ex}(\gamma )\in [0,n^*]$, $\gamma \in \Gamma _{<}\cup \Gamma _{=}$: $\text  {ac}^{\text  {in}}(\gamma )$ (resp., $\text  {ac}^\text  {ex}(\gamma )$) represents $\text  {ac}_{\gamma }^{\text  {in}}(G)$ (resp., $\text  {ac}_{\gamma }^\text  {ex}(G)$);
- $\text  {bc}^{\text  {in}}(\mu ), \text  {bc}^\text  {ex}(\mu )\in [0,n^*-1]$, $\mu \in \text  {Bc}$: $\text  {bc}^{\text  {in}}(\mu )$ (resp., $\text  {bc}^\text  {ex}(\mu )$) represents $\text  {bc}_{\mu }^{\text  {in}}(G)$ (resp., $\text  {bc}_{\mu }^\text  {ex}(G)$);

*constraints *

2 $$\begin{aligned} \text  {LB}_\text  {dg}^\text  {t}(i)&\le \text  {dg}^\text  {t}(i) \le \text  {UB}_\text  {dg}^\text  {t}(i),\quad i\in [1,4], ~ \text  {t}\in \{{\text  {in}},\text  {ex}\}, \end{aligned}$$

3 $$\begin{aligned} \text  {LB}_\text  {ce}^\text  {t}({\mathtt{a}})&\le \text  {ce}^\text  {t}({\mathtt{a}}) \le \text  {UB}_\text  {ce}^\text  {t}({\mathtt{a}}) ,\quad {\mathtt{a}}\in \Lambda , ~ \text  {t}\in \{{\text  {in}},\text  {ex}\}, \end{aligned}$$

4 $$\begin{aligned} \text  {LB}_\text  {bd}^\text  {t}(m)&\le \text  {bd}^\text  {t}(m) \le \text  {UB}_\text  {bd}^\text  {t}(m),\quad m\in [2,3], ~ \text  {t}\in \{{\text  {in}},\text  {ex}\}, \end{aligned}$$

5 $$\begin{aligned} \text  {LB}_\text  {ac}^\text  {t}(\gamma )&\le \text  {ac}^\text  {t}(\gamma ) \le \text  {UB}_\text  {ac}^\text  {t}(\gamma ),\quad \gamma \in \Gamma , ~ \text  {t}\in \{{\text  {in}},\text  {ex}\}, \end{aligned}$$

6 $$\begin{aligned} \text  {LB}_\text  {bc}^\text  {t}(\mu )&\le \text  {bc}^\text  {t}(\mu ) \le \text  {UB}_\text  {bc}^\text  {t}(\mu ),\quad \mu \in \text  {Bc}, ~ \text  {t}\in \{{\text  {in}},\text  {ex}\}. \end{aligned}$$

We use the range-based method to define an applicability domain for our method. For this, we find the range (the minimum and maximum) of each descriptor over all relevant chemical compounds and represent each range as a set of linear constraints in the constraint set ${\mathcal {C}}_1$ of our MILP formulation. Recall that $D_{\pi }$ stands for a set of chemical graphs used for constructing a prediction function. However, the number of examples in $D_{\pi }$ may not be large enough to capture a general feature on the structure of chemical graphs. For this, we also use some data set from the whole set $\text  {DB}$ of chemical graphs in a database. Let $\text  {DB}_{{\mathcal {G}}}^{(i)}$ denote the set of chemical graphs $G\in \text  {DB}\cap {\mathcal {G}}$ such that $n(G)=i$ for each integer $i\ge 1$. Based on this, we assume that the given lower and upper bounds on the above descriptors satisfy the following. For each $\text  {t}\in \{{\text  {in}},\text  {ex}\}$,

7 $$\begin{aligned}&\min _{G\in D_{\pi }\cup \text  {DB}_{{\mathcal {G}}}^{(n^*)}} \frac{\text  {dg}_i^\text  {t}(G)}{n(G)} \le \frac{\text  {LB}_\text  {dg}^\text  {t}(i) }{n^*} \le \frac{\text  {UB}_\text  {dg}^\text  {t}(i) }{n^*} \le \max _{G\in D_{\pi }\cup \text  {DB}_{{\mathcal {G}}}^{(n^*)}} \frac{\text  {dg}_i^\text  {t}(G)}{n(G)}, \nonumber \\&\quad i\in [1,4], \end{aligned}$$

8 $$\begin{aligned}&\min _{G\in D_{\pi }\cup \text  {DB}_{{\mathcal {G}}}^{(n^*)}} \frac{\text  {ce}_{\mathtt{a}}^\text  {t}(G)}{n(G)} \le \frac{ \text  {LB}_\text  {ce}^\text  {t}({\mathtt{a}}) }{n^*} \le \frac{ \text  {UB}_\text  {ce}^\text  {t}({\mathtt{a}}) }{n^*} \le \max _{G\in D_{\pi }\cup \text  {DB}_{{\mathcal {G}}}^{(n^*)}} \frac{\text  {ce}_{\mathtt{a}}^\text  {t}(G)}{n(G)}, \nonumber \\&\quad {\mathtt{a}}\in \Lambda , \end{aligned}$$

9 $$\begin{aligned}&\min _{G\in D_{\pi }\cup \text  {DB}_{{\mathcal {G}}}^{(n^*)}} \frac{\text  {bd}_m^\text  {t}(G)}{n(G) - 1} \le \frac{ \text  {LB}_\text  {bd}^\text  {t}(m) }{ n^* - 1} \le \frac{ \text  {UB}_\text  {bd}^\text  {t}(m) }{ n^* - 1} \le \max _{G\in D_{\pi }\cup \text  {DB}_{{\mathcal {G}}}^{(n^*)}} \frac{\text  {bd}_m^\text  {t}(G)}{n(G) - 1}, \nonumber \\&\quad m\in [2,3], \end{aligned}$$

10 $$\begin{aligned}&\min _{G\in D_{\pi }\cup \text  {DB}_{{\mathcal {G}}}^{(n^*)}} \frac{\text  {ac}_{\gamma }^\text  {t}(G)}{n(G) - 1} \le \frac{ \text  {LB}_\text  {ac}^\text  {t}(\gamma )}{ n^* - 1} \le \frac{ \text  {UB}_\text  {ac}^\text  {t}(\gamma )}{ n^* - 1} \le \max _{G\in D_{\pi }\cup \text  {DB}_{{\mathcal {G}}}^{(n^*)}} \frac{\text  {ac}_{\gamma }^\text  {t}(G)}{n(G) - 1}, \nonumber \\&\quad \gamma \in \Gamma , \end{aligned}$$

11 $$\begin{aligned}&\min _{G\in D_{\pi }\cup \text  {DB}_{{\mathcal {G}}}^{(n^*)}} \frac{\text  {bc}_{\mu }^\text  {t}(G)}{n(G) - 1} \le \frac{\text  {LB}_\text  {bc}^\text  {t}(\mu )}{ n^* - 1} \le \frac{\text  {UB}_\text  {bc}^\text  {t}(\mu )}{ n^* - 1} \le \max _{G\in D_{\pi }\cup \text  {DB}_{{\mathcal {G}}}^{(n^*)}} \frac{\text  {bc}_{\mu }^\text  {t}(G)}{n(G) - 1}, \nonumber \\&\quad \mu \in \text  {Bc}. \end{aligned}$$

### Construction of scheme graph

We infer a subgraph *H* such that the maximum degree is $d_\text  {max}\in \{3,4\}$, $n(H)=n^*$, $\text  {bh}_{k^*}(H)=\text  {bh}^*$, and ${\text  {bl}}_{k^*}(H)={\text  {bl}}^*$. For this, we first construct the scheme graph $\text  {SG}( d_\text  {max}, k^*, \text  {bh}^*, t^*)$. We then prepare a binary variable *u*(*s*, *i*) (resp., *v*(*t*, *i*)) for each vertex $u_{s,i}$ in tree $S_s$ (resp., $v_{t,i}$ in tree $T_t$).

Recall that when the two end-vertices of edge $$a_i=(u_{s,1},u_{s',1}) \in E_B=\{a_1,a_2,\ldots ,a_{c^*}\}$$ is connected in a selected subgraph *H*, either edge $$a_i$$ is directly used in *H* or a path $$P_i=(u_{s,1},v_{t',1},v_{t'+1,1},\ldots ,$$ $$v_{t'',1},u_{s',1})$$ from $$u_{s,1}$$ to $$u_{s',1}$$ visiting some vertices in $$P_{t^*}$$ is constructed in *H*. We regard the index *i* of each edge $$a_i\in E_B=\{a_1,a_2,\ldots ,a_{c^*}\}$$ as the “color” of the edge, and define the color set of $$E_B$$ to be $$[1,c^*]$$. To introduce necessary linear constraints that can construct such a path $$P_i$$ properly in our MILP, we assign the color *i* to the vertices $$v_{t',1},v_{t'+1,1},\ldots ,$$ $$v_{t'',1}$$ in $$P_{t^*}$$ when a path $$P_i=(u_{s,1},v_{t',1},v_{t'+1,1},\ldots ,$$ $$v_{t'',1},u_{s',1})$$ is used in *H*.

*constants *

Integers $$d_\text  {max}\in \{3,4\}$$, $$n^*\ge 3$$, $$\text  {dia}^*\ge 3$$, $$k^*\ge 1$$, $$\text  {bh}^*\ge 1$$ and $${\text  {bl}}^*\ge 2$$;

*variables *

- $a(i)\in \{0,1\}$, $i\in E_B$: *a*(*i*) represents edge $a_i\in E_B$ ($a(i)=1$, $i\in E_B$) ($a(i)=1$ $\Leftrightarrow$ edge $a_i$ is used in *H*);
- $e(s,t),e(t,s)\in \{0,1\}$, $s\in [1,s^*]$, $t\in [1,t^*]$: *e*(*s*, *t*) (resp., *e*(*t*, *s*)) represents direction $(u_{s,1}, v_{t,1})$ (resp., $(v_{t,1}, u_{s,1})$), where $e(s,t)=1$ (resp., $e(t,s)=1$) $\Leftrightarrow$ edge $u_{s,1},v_{t,1}$ is used in *H* and direction $(u_{s,1}, v_{t,1})$ (resp., $(v_{t,1}, u_{s,1})$) is assigned to edge $u_{s,1} v_{t,1}$;
- $\chi (t)\in [0,c^*]$, $t\in [1,t^*]$: $\chi (t)$ represents the color $c\in [0,c^*]$ assigned to vertex $v_{t,1}$ ($\chi (t)=c$ $\Leftrightarrow$ vertex $v_{t,1}$ is assigned color *c*, where $\chi (t)=c=0$ iff $v_{t,1}$ is not in *H*);
- $$\delta _{\text  {clr}}(t,c)\in \{0,1\}$$, $$t\in [1,t^*]$$, $$c\in [0,c^*]$$ ($$\delta _{\text  {clr}}(t,c)=1$$ $$\Leftrightarrow $$ $$\chi (t)=c$$);
- $\text  {clr}(c)\in [0,t^*]$, $c\in [0,c^*]$: the number of vertices $v_{t,i}$ with color *c*;
- $\deg ^\text  {b+}(s)\in [0,4]$, $s\in [1,s^*]$: the out-degree of vertex $u_{s,1}$ in the $k^*$-branch-subtree of *H*;
- $\deg ^\text  {b-}(s)\in [0,4]$, $s\in [1,s^*]$: the in-degree of vertex $u_{s,1}$ in the $k^*$-branch-subtree of *H*;

*constraints *

12 $$\begin{aligned}&\sum _{c\in [0,c^*]} \delta _\text  {clr}(t,c)=1, ~ \sum _{c\in [0,c^*]}c\cdot \delta _\text  {clr}(t,c)=\chi (t), \quad t\in [1,t^*],\end{aligned}$$

13 $$\begin{aligned}&\sum _{t\in [1,t^*]} \delta _\text  {clr}(t,c)=\text  {clr}(c), \quad c\in [0,c^*], \end{aligned}$$

14 $$\begin{aligned}&t^*(1-a(i))\ge \text  {clr}(i), \quad i\in [1,c^*], \end{aligned}$$

15 $$\begin{aligned}&e(s,t)+e(t,s)\le 1, \quad s\in [1,s^*], t\in [1,t^*], \end{aligned}$$

16 $$\begin{aligned}&\sum _{s\in [1,s^*]\setminus \{\text  {head}(c)\}} e(t,s)\le 1-\delta _\text  {clr}(t,c), \sum _{s\in [1,s^*]\setminus \{\text  {tail}(c)\}} e(s,t)\le 1-\delta _\text  {clr}(t,c), \nonumber \\&\quad c\in [1,c^*], t\in [1,t^*], \end{aligned}$$

17 $$\begin{aligned} {} & \sum _{ i\in E_B^-(s)}a(i)+ \sum _{t\in [1,t^*] } e(t,s) = \deg ^{\text  {b-}}(s), ~~ \sum _{ i\in E_B^+(s)}a(i)+ \sum _{t\in [1,t^*] } e(s,t) = \deg ^{\text  {b+}}(s),\nonumber \\&\quad \deg ^{\text  {b-}}(s)+\deg ^{\text  {b+}}(s) \le d_{\text  {max}}, ~~ s\in [1,s^*]. \end{aligned}$$

### Selecting a subgraph

From the scheme graph $\text  {SG}( d_\text  {max}, k^*, \text  {bh}^*, t^*)$, we select a subgraph *H* such that $n(H)=n^*$, $\text  {dia}(H)=\text  {dia}^*$, $\text  {bh}_{k^*}(H)=\text  {bh}^*$, and ${\text  {bl}}_{k^*}(H)={\text  {bl}}^*$.

*constants *

- Integers $$d_\text  {max}\in \{3,4\}$$, $$n^*\ge 3$$, $$\text  {dia}^*\ge 3$$, $$k^*\ge 1$$, $$\text  {bh}^*\ge 1$$ and $${\text  {bl}}^*\ge 2$$;
- For each tree $S_s=T(d_\text  {max}-1, d_\text  {max}-1, k^*)$, prepare
  - the set $\text  {Cld}_{\text  {S}}(i)$ of the indices of children of a vertex $v_i$;
  - the index $\text  {prt}(i)$ of the parent of a non-root vertex $v_i$;
  - the set $\text  {Dsn}_S(d)$ of indices *i* of a vertex $v_i$ whose depth is *d*;
  - a proper set $P_{\text  {prc}}(d_\text  {max}-1, d_\text  {max}-1, k^*)$ of index pairs,
  - where we denote $P_{\text  {prc}}(d_\text  {max}-1, d_\text  {max}-1, k^*)$ by $P_{S,\text  {prc}}$;
- For each tree $T_t=T(d_\text  {max}-2, d_\text  {max}-1, k^*)$, prepare
  - the set $\text  {Cld}_{\text  {T}}(i)$ of the indices of children of a vertex $v_i$;
  - the index $\text  {prt}(i)$ of the parent of a non-root vertex $v_i$;
  - a proper set $P_{\text  {prc}}(d_\text  {max}-2, d_\text  {max}-1, k^*)$ of index pairs,
  - where we denote $P_{\text  {prc}}(d_\text  {max}-2, d_\text  {max}-1, k^*)$ by $P_{T,\text  {prc}}$;

*variables *

- $$\sigma (s)\in \{0,1\}$$, $$s\in [1,s^* ]$$: ($$\sigma (s)=1$$$$\Leftrightarrow $$ vertex $$u_{s,1}$$ is a non-leaf $$k^*$$-branch or a root);
- $u(s,i)\in \{0,1\}$, $s\in [1,s^* ]$, $i\in [1,n_\text  {tree}^{\text  {S}}]$: *u*(*s*, *i*) represents vertex $u_{s,i}$ ($u(s,i)=1$ $\Leftrightarrow$ vertex $u_{s,i}$ is used in *H* and edge $e'_{s,i}$ $(i\ge 2)$ is used in *H*), ($u(s,1)=1$ and $\sigma (s)=0$ $\Leftrightarrow$ vertex $u_{s,1}$ is a leaf $k^*$-branch);
- $v(t,i)\in \{0,1\}$, $t\in [1,t^* ]$, $i\in [1,n_\text  {tree}^{\text  {T}}]$: *v*(*t*, *i*) represents vertex $v_{t,i}$ ($v(t,i)=1$ $\Leftrightarrow$ vertex $v_{t,i}$ is used in *H* and edge $e_{t,i}$ $(i\ge 2)$ is used in *H*);
- $e(t)\in \{0,1\}$, $t\in [1,t^*+1]$: *e*(*t*) represents edge $e_{t,1}=v_{t-1,1} v_{t,1}$, where $e_{1,1}$ and $e_{t^*+1,1}$ are fictitious edges ($e(t)=1$ $\Leftrightarrow$ edge $e_{t,1}$ is used in *H*);

*constraints *

18 $$\begin{aligned} {} & u(s,i)\ge u(s,j), ~~s\in [1,s^*], (i,j)\in P_{S,\text  {prc}}, \end{aligned}$$

19 $$\begin{aligned} {} & v(t,i)\ge v(t,j), ~~t\in [1,t^*], (i,j)\in P_{T,\text  {prc}}, \end{aligned}$$

20 $$\begin{aligned} {} & \sum _{ s\in [1,s^*], i\in [1, n_{\text  {tree}}^{\text  {S}}]}u(s,i) + \sum _{t\in [1,t^*], i\in [1, n_{\text  {tree}}^{\text  {T}}]}v(t,i)=n^*, \end{aligned}$$

21 $$\begin{aligned} {} & \sum _{i\in [1, n_{\text  {tree}}^{\text  {S}}]}u(s,i) \le 2+ 2 \sum _{j\in \text  {Cld}_{\text  {S}}(1)}u(s,j), \quad s\in [1,s^*], \end{aligned}$$

22 $$\begin{aligned} {} & \sum _{i\in [1, n_{\text  {tree}}^{\text  {T}}]}v(t,i) \le 2+ 2 \sum _{j\in \text  {Cld}_{\text  {T}}(1)}v(t,j), \quad t\in [1,t^*], \end{aligned}$$

23 $$\begin{aligned} {} & e(t+1)+\sum _{s\in [1,s^*]}e(t,s) =v(t,1) , ~~ e(t) +\sum _{s\in [1,s^*]}e(s,t) =v(t,1) , \nonumber \\&\sum _{c\in [1,c^*]} \delta _\text  {clr}(t,c) = v(t,1), \nonumber \\&\quad \text{(where } e(1)=e(t^*+1)=0), \quad t\in [1,t^*], \end{aligned}$$

24 $$\begin{aligned} {} & c^*\cdot ( 1-e(t+1)) \ge \chi (t) - \chi (t+1) \ge v(t,1) -e(t+1), \quad t\in [1,t^*-1], \end{aligned}$$

25 $$\begin{aligned} {} & a(i)+\sum _{t\in [1,t^*]} e(t,i+1)= u(i+1,1), \quad i\in [1,c^*], \end{aligned}$$

26 $$\begin{aligned} {} & \sigma (s)\le u(s,1), \quad s\in [1,s^*], \end{aligned}$$

27 $$\begin{aligned} {} & \sigma (s)=u(s,1)=1, \text{ if } u_s \text{ is } \text{ the } \text{ root }, \end{aligned}$$

28 $$\begin{aligned} {} & (d_{\text  {max}}-1)\sigma (s)\ge \sum _{s'\in {\text  {Cld}_B(s)}} u(s',1)\ge 2\sigma (s), ~ \sum _{i\in {\text  {Dsn}}_S(k^*)} u(s,i)\ge u(s,1)-\sigma (s),  \\&\quad s\in [1,s^*], u_s\ne {\text  {root}}, \end{aligned}$$

29 $$\begin{aligned} {} & \sum _{ s\in [2,s^*] }(u(s,1) -\sigma (s)) = {\text  {bl}}^* , ~~ \sum _{s\in V_B(\text  {bh}^*)} u(s,1) \ge 1, \end{aligned}$$

30 $$\begin{aligned} {} & \sum _{s\in V_{B,s^\text  {left}}} u(s,1) + \sum _{i\in E_{B,s^\text  {left}}} \text  {clr}(i) = \Big \lceil \frac{\text  {dia}^*}{2} \Big \rceil - k^*, \end{aligned}$$

31 $$\begin{aligned} {} & \sum _{s\in V_{B,s^\text  {right}}} u(s,1) + \sum _{i\in E_{B,s^\text  {right}}} \text  {clr}(i) = \Big \lfloor \frac{\text  {dia}^*}{2} \Big \rfloor - k^*, \end{aligned}$$

32 $$\begin{aligned} {} & \sum _{i\in V_{B,s}} u(i,1) + \sum _{i\in E_{B,s}} \text  {clr}(i) \le \Big \lfloor \frac{\text  {dia}^*}{2} \Big \rfloor - k^*, \quad s\in L_B\setminus \{s^\text  {left},s^\text  {right}\}. \end{aligned}$$

Constraints (21) and (22) represent an extension of constraint (1) on the size of 2-fringe-trees to the case of a general branch-parameter $k^*$.

### Assigning multiplicity

We prepare an integer variable ${\widetilde{\beta }}(e)$ or ${\widehat{\beta }}(e)$ for each edge *e* in the scheme graph $\text  {SG}( d_\text  {max}, k^*, \text  {bh}^*, t^*)$ to denote the multiplicity of *e* in a selected graph *H* and include necessary constraints for the variables to satisfy in *H*.

*constants *

- Prepare functions $\text  {tail}$ and $\text  {head}$ such that $a_{i}=(u_{\text  {tail}(i)}, u_{\text  {head}(i)})\in E_B$;
- Assume that each edge in a tree $S_s$, $s\in [1,s^*]$ (resp., $T_t$, $t\in [1,t^*]$) is denoted by $e'_{s,i}$ (resp., $e_{t,i}$) with the integer $i\in [2,n_\text  {tree}^{\text  {S}}]$ of the head $u_{s,i}$ (resp., $v_{t,i}$) of the edge;

*variables *

- ${\widetilde{\beta }}(i)\in [0,3]$, $i\in [1,c^*]$: ${\widetilde{\beta }}(i)$ represents the multiplicity of edge $a_{i}$, where ${\widetilde{\beta }}(i)=0$ if edge $a_{i}$ is not in an inferred chemical graph *G*;
- ${\widetilde{\beta }}(p,i)\in [0,3]$, $p\in [1,s^*+ t^*]$, $i\in [2,n_\text  {tree}^{\text  {S}}]$: ${\widetilde{\beta }}(p,i)$ with $p\le s^*$ (resp., $p>s^*$) represents the multiplicity of edge $e'_{p,i}$ (resp., $e_{p-s^*,i}$);
- ${\widetilde{\beta }}(t,1)\in [0,3]$, $t\in [1, t^*+1]$: ${\widetilde{\beta }}(t,1)$ represents the multiplicity of edge $e_{t,1}$;
- ${\widehat{\beta }}(s,t)\in [0,3]$, $s\in [1,s^*]$, $t\in [1,t^*]$: ${\widehat{\beta }}(s,t)$ represents the multiplicity of edge $u_{s,1}v_{t,1}$;

*constraints *

33 $$\begin{aligned} {} & a(i)\le {\widetilde{\beta }}(i)\le 3 a(i), \quad i\in [1,c^*], \end{aligned}$$

34 $$\begin{aligned} {} & u(s,i) \le {\widetilde{\beta }}(s,i)\le 3 u(s,i), \quad s\in [1,s^*], i\in [2,n_\text  {tree}^{\text  {S}}], \end{aligned}$$

35 $$\begin{aligned} {} & v(t,i)\le {\widetilde{\beta }}(s^*+ t,i)\le 3 v(t,i), \quad t\in [1,t^*], i\in [2,n_\text  {tree}^{\text  {T}}], \end{aligned}$$

36 $$\begin{aligned} {} & e(t)\le {\widetilde{\beta }}(t,1)\le 3 e(t), \quad t\in [1,t^*+1], \end{aligned}$$

37 $$\begin{aligned} {} & e(s,t)+e(t,s)\le {\widehat{\beta }}(s,t)\le 3 e(s,t)+3 e(t,s) , \quad s\in [1,s^*], t\in [1,t^*]. \end{aligned}$$

### Assigning chemical elements and valence condition

We include constraints so that each vertex *v* in a selected graph *H* satisfies the valence condition; i.e., $\beta (v)\le {\text  {val}}(\alpha (v))$. With these constraints, a chemical acyclic graph $G=(H,\alpha ,\beta )$ on a selected subgraph *H* will be constructed.

*constants *

- A set $\Lambda \cup \{\epsilon \}$ of chemical elements, where $\epsilon$ denotes null;
- A coding $[{\mathtt{a}}]$, ${\mathtt{a}}\in \Lambda \cup \{\epsilon \}$ such that $[\epsilon ]=0$; $[{\mathtt{a}}]\ge 1$, ${\mathtt{a}}\in \Lambda$; and $[{\mathtt{a}}]\ne [\mathtt{b}]$ if ${\mathtt{a}}\ne \mathtt{b}$; Let $[\Lambda ]$ and $[\Lambda \cup \{\epsilon \}]$ denote $\{[{\mathtt{a}}]\mid {\mathtt{a}}\in \Lambda \}$ and $\{[{\mathtt{a}}]\mid {\mathtt{a}}\in \Lambda \cup \{\epsilon \}\}$, respectively;
- A valence function: $${\text  {val}}: \Lambda \rightarrow [1,4]$$;
- Let $E_B(s)$ denote the set of indices *i* of all edges $a_i\in E_B$ adjacent to vertex $u_{s,1}$ in $T_B$.

*variables *

- ${\widetilde{\alpha }}(p,i)\in [\Lambda \cup \{\epsilon \}]$, $p\in [1,s^*+ t^*]$, $i\in [1,n_\text  {tree}^{\text  {S}}]$: ${\widetilde{\alpha }}(p,i)$ with $p\le s^*$ (resp., $p>s^*$) represents $\alpha (u_{p,i})$ (resp., $\alpha (v_{p-s^*,i})$);
- $$\delta _{\alpha }(p,i,{\mathtt{a}})\in \{0,1\}$$, $$p\in [1,s^*+ t^*]$$, $$i\in [1,n_\text  {tree}^{\text  {S}}]$$, $${\mathtt{a}}\in \Lambda \cup \{\epsilon \}$$: $$\delta _{\alpha }(p,i,{\mathtt{a}})=1$$$$\Leftrightarrow $$$$\alpha (u_{p,i})={\mathtt{a}}$$ for $$p\le s^*$$ and $$\alpha (v_{p-s^*,i})={\mathtt{a}}$$ for $$p> s^*$$;
- $$\delta _{{\widetilde{\beta }}}(i,m)\in \{0,1\}$$, $$p\in [1,s^*+ t^*]$$, $$i\in [1,c^*]$$, $$m\in [0,3]$$: $$\delta _{{\widetilde{\beta }}}(i,m)=1$$$$\Leftrightarrow $$ the multiplicity of edge $$a_i$$ in an inferred chemical graph *G* is *m*;
- $$\delta _{{\widetilde{\beta }}}(p,i,m)\in \{0,1\}$$, $$p\in [1,s^*+ t^*]$$, $$i\in [2,n_\text  {tree}^{\text  {S}}]$$, $$m\in [0,3]$$: $$\delta _{{\widetilde{\beta }}}(p,i,m)=1$$$$\Leftrightarrow $$ the multiplicity of edge $$e'_{p,i}$$, $$p\le s^*$$ (or $$e_{p-s^*,i}$$, $$p>s^*$$) in *G* is *m*;
- $$\delta _{{\widetilde{\beta }}}(t,1,m)\in \{0,1\}$$, $$t\in [1, t^*+1]$$, $$m\in [0,3]$$: $$\delta _{{\widetilde{\beta }}}(t,1,m)=1$$$$\Leftrightarrow $$ the multiplicity of edge $$e_{t}$$ in *G* is *q*;
- $$\delta _{{\widehat{\beta }}}(s,t,m)\in \{0,1\}$$, $$s\in [1,s^*]$$, $$t\in [1,t^*]$$, $$m\in [0,3]$$: $$\delta _{{\widehat{\beta }}}(s,t,m)=1$$$$\Leftrightarrow $$ the multiplicity of edge $$u_{s,1}v_{t,1}$$ in *G* is *m*;

*constraints *

38 $$\begin{aligned} {} & \sum _{{\mathtt{a}}\in \Lambda \cup \{\epsilon \}} \delta _{\alpha }(p,i,{\mathtt{a}})=1, \quad p\in [1,s^*+ t^*], i\in [1,n_\text  {tree}^{\text  {S}}], \end{aligned}$$

39 $$\begin{aligned} {} & \sum _{{\mathtt{a}}\in \Lambda \cup \{\epsilon \}}[{\mathtt{a}}]\cdot \delta _{\alpha }(p,i,{\mathtt{a}}) ={\widetilde{\alpha }}(p,i), \quad p\in [1,s^*+ t^*], i\in [1,n_\text  {tree}^{\text  {S}}], \end{aligned}$$

40 $$\begin{aligned} {} & \sum _{m\in [0,3]} \delta _{{\widetilde{\beta }}}(i,q)=1, ~~~ \sum _{m\in [1,3]} m\cdot \delta _{{\widetilde{\beta }}}(i,m)={\widetilde{\beta }}(i), \quad i\in [1,c^*], \end{aligned}$$

41 $$\begin{aligned} {} & \sum _{m\in [0,3]} \delta _{{\widetilde{\beta }}}(p,i,m)= 1, ~~ \sum _{m\in [1,3]} m\cdot \delta _{{\widetilde{\beta }}}(p,i,m)={\widetilde{\beta }}(p,i), \nonumber \\&\quad p\in [1,s^*+ t^*], i\in [2,n_\text  {tree}^{\text  {S}}], \end{aligned}$$

42 $$\begin{aligned} {} & \sum _{m\in [0,3]} \delta _{{\widetilde{\beta }}}(t,1,q)= 1, ~ \sum _{m\in [1,3]} m \cdot \delta _{{\widetilde{\beta }}}(t,1,m)= {\widetilde{\beta }}(t,1), \quad t\in [1,t^*+1], \end{aligned}$$

43 $$\begin{aligned} {} & \sum _{m\in [0,3]} \delta _{{\widehat{\beta }}}(s,t,m)= 1, ~ \sum _{m\in [0,3]} m\delta _{{\widehat{\beta }}}(s,t,m)= {\widehat{\beta }}(s,t), \nonumber \\&\quad s\in [1,s^*], t\in [1,t^*], \end{aligned}$$

44 $$\begin{aligned} {} & \sum _{i\in E_B(s)} {\widetilde{\beta }}(i) +\sum _{t\in [1,t^*]}{\widehat{\beta }}(s,t) +\sum _{j\in \text  {Cld}_{\text  {S}}(1)}{\widetilde{\beta }}(s,j) \le \sum _{{\mathtt{a}}\in \Lambda }{\text  {val}}({\mathtt{a}}) \cdot \delta _{\alpha }(s,1,{\mathtt{a}}), \nonumber \\&\quad s\in [1,s^*], \end{aligned}$$

45 $$\begin{aligned} {} & \sum _{s\in [1,s^*]}{\widehat{\beta }}(s,t) +{\widetilde{\beta }}(t,1) + {\widetilde{\beta }}(t+1,1) + \sum _{j\in \text  {Cld}_{\text  {T}}(1)} {\widetilde{\beta }}(s^*+ t,j) \le \sum _{{\mathtt{a}}\in \Lambda }{\text  {val}}({\mathtt{a}})\delta _{\alpha }(s^*+ t,1,{\mathtt{a}}), \nonumber \\&\quad t\in [1,t^*], \end{aligned}$$

46 $$\begin{aligned} {} & {\widetilde{\beta }}(s,i) +\sum _{j\in \text  {Cld}_{\text  {S}}(i)}{\widetilde{\beta }}(s,j) \le \sum _{{\mathtt{a}}\in \Lambda }{\text  {val}}({\mathtt{a}}) \delta _{\alpha }(s,i,{\mathtt{a}}), \quad s\in [1,s^*], i\in [2, n_\text  {tree}^{\text  {S}}], \end{aligned}$$

47 $$\begin{aligned} {} & {\widetilde{\beta }}(s^*+t,i) +\sum _{j\in \text  {Cld}_{\text  {T}}(i)}{\widetilde{\beta }}(s^*+t,j) \le \sum _{{\mathtt{a}}\in \Lambda }{\text  {val}}({\mathtt{a}}) \delta _{\alpha }(s^*+t,i,{\mathtt{a}}), \nonumber \\&\quad t\in [1,t^*], i\in [2, n_\text  {tree}^{\text  {T}}]. \end{aligned}$$

### Descriptors on mass, the numbers of elements and bonds

We include constraints to compute descriptors $\overline{\text  {ms}}(G)$, $\text  {ce}_{\mathtt{a}}(G)$ (${\mathtt{a}}\in \Lambda )$, $\text  {bd}_m(G)$ ($m\in [2,3]$) and $n_\mathtt{H}(G)$ according to the definitions in "Modeling of chemical compounds" section.

*constants *

- A function $\text  {mass}^*:\Lambda \rightarrow {\mathbb {Z}}$ (we let $\text  {mass}({\mathtt{a}})$ denote the observed mass of a chemical element ${\mathtt{a}}\in \Lambda$, and define $\text  {mass}^*({\mathtt{a}})=\lfloor 10\cdot \text  {mass}({\mathtt{a}})\rfloor$);

*variables *

- $$\text  {Mass}\in {\mathbb {Z}}$$: $$\text  {Mass}$$ represents $$\sum _{v\in V} \text  {mass}^*(\alpha (v))$$;
- $$\text  {bd}(m)\in [0,2n^*]$$, $$m\in [1,3]$$;
- $\text  {n}_\mathtt{H}\in [0,4n^*]$: the number $n_\mathtt{H}(G)$ of hydrogen atoms to be included to *G*;

*constraints *

48 $$\begin{aligned} {} & \sum _{p\in [1,s^*+ t^*]} \delta _{\alpha }(p,1,{\mathtt{a}}) = \text  {ce}^{\text  {in}}({\mathtt{a}}), ~~~ \sum _{p\in [1,s^*+ t^*], i\in [2, n_\text  {tree}^{\text  {S}}] } \delta _{\alpha }(p,i,{\mathtt{a}})= \text  {ce}^\text  {ex}({\mathtt{a}}), \nonumber \\&\quad {\mathtt{a}}\in \Lambda , \end{aligned}$$

49 $$\begin{aligned} {} & \sum _{{\mathtt{a}}\in \Lambda }\text  {mass}^*({\mathtt{a}}) (\text  {ce}^{\text  {in}}({\mathtt{a}})+\text  {ce}^\text  {ex}({\mathtt{a}})) =\text  {Mass}, \end{aligned}$$

50 $$\begin{aligned} {} & \sum _{i\in [1,c^*]} \delta _{{\widetilde{\beta }}}(i,q) + \sum _{s\in [1,s^*], t\in [1,t^*]} \delta _{{\widehat{\beta }}}(s,t,q) + \sum _{ t\in [2,t^*]}\delta _{{\widetilde{\beta }}}(t,1,q) = \text  {bd}^{\text  {in}}(m), \nonumber \\&\sum _{p\in [1,s^*+ t^*], i\in [2, n_\text  {tree}^{\text  {S}}] } \delta _{{\widetilde{\beta }}}(p,i,m) = \text  {bd}^\text  {ex}(m), \nonumber \\&\quad m\in [1,3], \end{aligned}$$

51 $$\begin{aligned} {} & \sum _{{\mathtt{a}}\in \Lambda }{\text  {val}}({\mathtt{a}}) (\text  {ce}^{\text  {in}}({\mathtt{a}}) + \text  {ce}^\text  {ex}({\mathtt{a}}) ) - 2(n^*-1+\text  {bd}^{\text  {in}}(2)+\text  {bd}^\text  {ex}(2) \nonumber \\&\quad + 2\text  {bd}^{\text  {in}}(3) + 2\text  {bd}^\text  {ex}(3) ) =\text  {n}_\mathtt{H}. \end{aligned}$$

### Descriptor for the Number of Specified Degree

We include constraints to compute descriptors $\text  {dg}_i(G)$ ($i\in [1,4]$) according to the definitions in "Modeling of chemical compounds" section. We also add constraints so that the maximum degree of a vertex in *H* is at most 3 (resp., equal to 4) when $d_\text  {max}=3$ (resp., $d_\text  {max}=4)$.

*variables *

- $\deg (p,i)\in [0,4]$, $p\in [1,s^*+ t^*]$, $i\in [1,n_\text  {tree}^{\text  {S}}]$: $\deg (p,i)$ represents $\deg _H(u_{p,i})$ for $p\le s^*$ or $\deg _H(v_{p-s^*,i})$ for $p> s^*$;
- $$\delta _{\deg }(p,i,d)\in \{0,1\}$$, $$p\in [1,s^*+ t^*]$$, $$i\in [1,n_\text  {tree}^{\text  {S}}]$$, $$d\in [0,4]$$: $$\delta _{\deg }(p,i,d)=1$$$$\Leftrightarrow $$$$\deg (p,i)=d$$;

*constraints *

52 $$\begin{aligned} {} & \sum _{ i\in E_B(s)}a(i)+ \sum _{t\in [1,t^*] }(e(s,t)+e(t,s)) + \sum _{j\in {\text  {Cld}}_{\text  {S}}(1)}u(s,j) =\deg (s,1),  \\&\quad s\in [1,s^*], \end{aligned}$$

53 $$\begin{aligned} {} & u(s,i)+ \sum _{j\in {\text  {Cld}}_{\text  {S}}(i)}u(s,j)=\deg (s,i), \quad s\in [1,s^*], i\in [2,n_{\text  {tree}}^{\text  {S}}], \end{aligned}$$

54 $$\begin{aligned} {} & 2v(t,1) + \sum _{j\in {\text  {Cld}}_{\text  {T}}(1)}v(t,j)=\deg (s^*+ t,1), \quad t\in [1,t^*], \end{aligned}$$

55 $$\begin{aligned} {} & v(t,i)+ \sum _{j\in {\text  {Cld}}_{\text  {T}}(i)}v(t,j)=\deg (s^*+ t,i), \quad t\in [1,t^*], i\in [2,n_{\text  {tree}}^{\text  {T}}], \end{aligned}$$

56 $$\begin{aligned} {} & \sum _{d\in [0,4]} \delta _{\deg }(p,i,d)= 1, ~~ \sum _{d\in [1,4]}d\cdot \delta _{\deg }(p,i,d)= \deg (p,i), \\&\quad p\in [1,s^*+ t^*], i\in [1,n_{\text  {tree}}^{\text  {S}}], \end{aligned}$$

57 $$\begin{aligned} {} & \sum _{p\in [1,s^*+ t^*]}\delta _{\deg }(p,1,d) = \text  {dg}^{\text  {in}}(d), ~~ \sum _{p\in [1,s^*+ t^*], i\in [2,n_{\text  {tree}}^{\text  {S}}]}\delta _{\deg }(p,i,d) = \text  {dg}^\text  {ex}(d), \nonumber \\&\quad d\in [1,4], \end{aligned}$$

58 $$\begin{aligned} {} & \text  {dg}^{\text  {in}}(4)+ \text  {dg}^\text  {ex}(4)\ge 1 \text{(resp., } = 0) \quad \text{ when } d_\text  {max}=4 \text{(resp., } =3). \end{aligned}$$

### Descriptor for the number of adjacency-configurations

We include constraints to compute descriptors $\text  {ac}_{\gamma }(G)$ ($\gamma =(\mathtt{a,b},m)\in \Gamma$) according to the definitions in "Modeling of chemical compounds" section.

*constants *

- A set $\Gamma =\Gamma _{<}\cup \Gamma _{=}\cup \Gamma _{>}$ of proper tuples $(\mathtt{a,b},m)\in \Lambda \times \Lambda \times [1,3]$;
- The set $$\Gamma _0=\{(\mathtt{a,b},0)\mid \mathtt{a,b}\in \Lambda \cup \{\epsilon \}\}$$;

*variables *

- $$\delta _{\tau }(i,\gamma )\in \{0,1\}$$, $$i\in [1,c^*]$$, $$\gamma \in \Gamma \cup \Gamma _0 $$: $$\delta _{\tau }(i, \gamma )=1$$$$\Leftrightarrow $$ edge $$a_{i}$$ is assigned tuple $$\gamma $$; i.e., $$\gamma =({\widetilde{\alpha }}(\text  {tail}(i),1), {\widetilde{\alpha }}(\text  {head}(i),1), {\widetilde{\beta }}(i))$$;
- $$\delta _{\tau }(t,1,\gamma )\in \{0,1\}$$, $$t\in [2,t^*]$$, $$\gamma \in \Gamma \cup \Gamma _0 $$: $$\delta _{\tau }(t,1, \gamma )=1$$$$\Leftrightarrow $$ edge $$e_{t,1}$$ is assigned tuple $$\gamma $$; i.e., $$\gamma =({\widetilde{\alpha }}(s^*+ t-1,1), {\widetilde{\alpha }}(s^*+ t,1), {\widetilde{\beta }}(t,1))$$;
- $$\delta _{\tau }(p,i,\gamma )\in \{0,1\}$$, $$p\in [1,s^*+ t^*]$$, $$i\in [2,n_\text  {tree}^{\text  {S}}]$$, $$\gamma \in \Gamma \cup \Gamma _0 $$: $$\delta _{\tau }(p,i, \gamma )=1$$$$\Leftrightarrow $$ edge $$e'_{p,i}$$, $$p\le s^*$$ (or $$e_{p-s^*,i}$$, $$p> s^*$$) is assigned tuple $$\gamma $$; i.e., $$\gamma =({\widetilde{\alpha }}(p,\text  {prt}(i)), {\widetilde{\alpha }}(p,i), {\widetilde{\beta }}(p,i))$$;
- $$\delta _{{\widehat{\tau }}}(s,t,\gamma )\in \{0,1\}$$, $$s\in [1,s^*]$$, $$t\in [1,t^*]$$, $$\gamma \in \Gamma \cup \Gamma _0 $$: $$\delta _{{\widehat{\tau }}}(s,t, \gamma )=1$$$$\Leftrightarrow $$ edge $$u_{s,1}v_{t,1}$$ is assigned tuple $$\gamma $$; i.e., $$\gamma =({\widetilde{\alpha }}(s,1), {\widetilde{\alpha }}(s^*+ t,1), {\widehat{\beta }}(s,t))$$;

*constraints *

59 $$\begin{aligned} {} & \sum _{(\mathtt{a,b},m)\in \Gamma \cup \Gamma _0 } [{\mathtt{a}}]\delta _{\tau }(i,(\mathtt{a,b},m))= {\widetilde{\alpha }}(\text  {tail}(i),1),\nonumber \\&\sum _{(\mathtt{a,b},m)\in \Gamma \cup \Gamma _0 } [\mathtt{b}]\delta _{\tau }(i,(\mathtt{a,b},m))= {\widetilde{\alpha }}(\text  {head}(i),1),\nonumber \\&\sum _{(\mathtt{a,b},m)\in \Gamma \cup \Gamma _0 } m\cdot \delta _{\tau }(i,(\mathtt{a,b},m))={\widetilde{\beta }}(i), \nonumber \\&\sum _{\gamma \in \Gamma \cup \Gamma _0 } \delta _{\tau }(i,\gamma )= 1,\quad i\in [1,c^*], \end{aligned}$$

60 $$\begin{aligned} {} & \sum _{(\mathtt{a,b},m)\in \Gamma \cup \Gamma _0 } [{\mathtt{a}}] \delta _\tau (t,1,(\mathtt{a,b},m) )= {\widetilde{\alpha }}(s^*+ t - 1,1),\nonumber \\&\sum _{(\mathtt{a,b},m)\in \Gamma \cup \Gamma _0 } [\mathtt{b}] \delta _\tau (t,1,(\mathtt{a,b},m) )= {\widetilde{\alpha }}(s^*+ t,1),\nonumber \\&\sum _{(\mathtt{a,b},m)\in \Gamma \cup \Gamma _0 } m\cdot \delta _\tau (t,1,(\mathtt{a,b},m) )= {\widetilde{\beta }}(t,1), \nonumber \\&\sum _{\gamma \in \Gamma \cup \Gamma _0 } \delta _{\tau }(t,1,\gamma )=1 , \quad t\in [2,t^*], \end{aligned}$$

61 $$\begin{aligned} {} & \sum _{(\mathtt{a,b},m)\in \Gamma \cup \Gamma _0 } [{\mathtt{a}}] \delta _\tau (p,i, (\mathtt{a,b},m) )= {\widetilde{\alpha }}(p,\text  {prt}(i)),\nonumber \\&\sum _{(\mathtt{a,b},m)\in \Gamma \cup \Gamma _0 } [\mathtt{b}] \delta _\tau (p,i, (\mathtt{a,b},m) )= {\widetilde{\alpha }}(p,i),\nonumber \\&\sum _{(\mathtt{a,b},m)\in \Gamma \cup \Gamma _0 } m\cdot \delta _\tau (p,i,(\mathtt{a,b},m) )= {\widetilde{\beta }}(p,i), \nonumber \\&\sum _{\gamma \in \Gamma \cup \Gamma _0 } \delta _{\tau }(p, i,\gamma )=1 , \quad p\in [1,s^*+ t^*], i\in [2, n_{\text  {tree}}^{\text  {S}}], \end{aligned}$$

62 $$\begin{aligned} {} & \sum _{(\mathtt{a,b},m)\in \Gamma \cup \Gamma _0 } [{\mathtt{a}}]\delta _{{\widehat{\tau }}}(s,t,(\mathtt{a,b},m))= {\widetilde{\alpha }}(s,1),\nonumber \\&\sum _{(\mathtt{a,b},m)\in \Gamma \cup \Gamma _0 } [\mathtt{b}]\delta _{{\widehat{\tau }}}(s,t,(\mathtt{a,b},m))= {\widetilde{\alpha }}(s^*+ t,1), \nonumber \\&\sum _{(\mathtt{a,b},m)\in \Gamma \cup \Gamma _0 } m\cdot \delta _{{\widehat{\tau }}}(s,t,(\mathtt{a,b},m))= {\widehat{\beta }}(s,t), \nonumber \\&\sum _{\gamma \in \Gamma \cup \Gamma _0 } \delta _{{\widehat{\tau }}}(s,t,\gamma )= 1, \quad s\in [1,s^*], t\in [1,t^*], \end{aligned}$$

63 $$\begin{aligned} {} & \sum _{i\in [1,c^*]} (\delta _\tau (i, \gamma )+\delta _\tau (i, \overline{\gamma } ) ) +\sum _{s\in [1,s^*], t\in [1,t^*]} (\delta _{{\widehat{\tau }}}(s,t, \gamma ) +\delta _{{\widehat{\tau }}}(s,t, \overline{\gamma } ) ) \nonumber \\&\quad +\sum _{t\in [2,t^*]} (\delta _\tau (t,1, \gamma )+\delta _\tau (t,1, \overline{\gamma } ) ) = \text  {ac}^{\text  {in}}(\gamma ), \quad \gamma \in \Gamma _{<}, \end{aligned}$$

64 $$\begin{aligned} {} & \sum _{i\in [1,c^*]} \delta _\tau (i, \gamma ) +\sum _{s\in [1,s^*], t\in [1,t^*]}\delta _{{\widehat{\tau }}}(s,t, \gamma ) + \sum _{t\in [2,t^*]} \delta _\tau (t,1, \gamma ) = \text  {ac}^{\text  {in}}(\gamma ), \nonumber \\&\quad \gamma \in \Gamma _{=}, \end{aligned}$$

65 $$\begin{aligned} {} & \sum _{p\in [1,s^*+ t^*], i\in [2, n_\text  {tree}^{\text  {S}}] } (\delta _\tau (p,i, \gamma )+\delta _\tau (p,i, \overline{\gamma } ) ) = \text  {ac}^\text  {ex}(\gamma ), \quad \gamma \in \Gamma _{<}, \end{aligned}$$

66 $$\begin{aligned} {} & \sum _{p\in [1,s^*+ t^*], i\in [2, n_\text  {tree}^{\text  {S}}] } \delta _\tau (p,i, \gamma ) = \text  {ac}^\text  {ex}(\gamma ), \quad \gamma \in \Gamma _{=}. \end{aligned}$$

### Descriptor for bond-configuration

We include constraints to compute the descriptors for bond-configuration $\text  {bd}_{\mu }(G)$, $\mu \in \text  {Bc}$, according to the definition.

*variables *

- $$\text  {bc}(\mu )\in [0,n^*-1]$$, $$\mu \in \text  {Bc}$$;
- $$\delta _{\text  {dc}}(i,d,d',m)\in \{0,1\}$$, $$i\in [1,c^*]$$, $$d,d'\in [0, 4]$$, $$m\in [0,3]$$: $$\delta _{\text  {dc}}(i,d,d',m)=1$$$$\Leftrightarrow $$$$\deg _H(u_{{\text  {tail}}(i)})=d$$, $$\deg _H(u_{{\text  {head}}(i)})=d'$$ and $$\beta (a_i)=m\in [1,3]$$ in *G*;
- $$\delta _{\text  {dc}}(t,1,d,d',m)\in \{0,1\}$$, $$t\in [2,t^*]$$, $$d,d'\in [0, 4]$$, $$m\in [0,3]$$: $$\delta _{\text  {dc}}(t,1,d,d',m)=1$$$$\Leftrightarrow $$$$\deg _H(v_{t-1,1})=d$$, $$\deg _H(v_{t,1})=d'$$ and $$\beta (e_{t,1})=m\in [1,3]$$ in *G*;
- $$\delta _{\text  {dc}}(p,i,d,d',m)\in \{0,1\}$$, $$p\in [1,s^*+ t^*]$$, $$i\in [2,n_\text  {tree}^{\text  {S}}]$$, $$d,d'\in [0, 4]$$, $$m\in [0,3]$$: $$\delta _{\text  {dc}}(p,i,d,d',m)=1$$$$\Leftrightarrow $$$$\deg _H(u_{p,\text  {prt}(i)})=d$$, $$\deg _H(u_{p,i})=d'$$ and $$\beta (e'_{p,i})=m\in [1,3]$$ for $$p\le s^*$$ (or $$\deg _H(v_{p-s^*,\text  {prt}(i)})=d$$, $$\deg _H(v_{p-s^*,i})=d'$$ and $$\beta (e_{p-s^*,i})=m\in [1,3]$$ for $$p> s^*$$) in *G*;
- $$\delta _{\widehat{\text  {dc}}}(s,t,d,d',m)\in \{0,1\}$$, $$s\in [1,s^*]$$, $$t\in [1,t^*]$$, $$d,d'\in [0, 4]$$, $$m\in [0,3]$$: $$\delta _{\widehat{\text  {dc}}}(s,t,d,d',1)=1$$$$\Leftrightarrow $$$$\deg _H(u_{s,1})=d$$, $$\deg _H(v_{t,1})=d'$$ and $$\beta (u_{s,1}v_{t,1})=m\in [1,3]$$ in *G*;

*constraints *

67 $$\begin{aligned} {} & \sum _{d,d'\in [0, 4],m\in [0,3]} \delta _{\text  {dc}}(i,d,d',m)=1, ~~ \sum _{d,d'\in [0, 4],m\in [0,3] } m\cdot \delta _{\text  {dc}}(i,d,d',m)={\widetilde{\beta }}(i), \\&\sum _{d\in [1,4],d'\in [0,4],m\in [0,3]} d\cdot \delta _{\text  {dc}}(i,d,d',m)=\deg (\text  {tail}(i),1), \\&\sum _{d\in [0,4],d'\in [1,4],m\in [0,3]} d'\cdot \delta _{\text  {dc}}(i,d,d',m)=\deg (\text  {head}(i),1), \quad i\in [1,c^*], \end{aligned}$$

68 $$\begin{aligned} {} & \sum _{d,d'\in [0, 4],m\in [0,3] } \delta _{\text  {dc}}(t,1,d,d',m)= 1, \nonumber \\&\sum _{d,d'\in [0, 4],m\in [0,3] } m\cdot \delta _{\text  {dc}}(t,1,d,d',m) ={\widetilde{\beta }}(t,1), \\&\sum _{d\in [1,4],d'\in [0,4],m\in [0,3]} d\cdot \delta _{\text  {dc}}(t,1,d,d',m) =\deg (s^*+ t-1,1),  \\&\sum _{d\in [0,4],d'\in [1,4],m\in [0,3]} d'\cdot \delta _{\text  {dc}}(t,1,d,d',m) =\deg (s^*+ t,1), \quad t\in [2,t^*], \end{aligned}$$

69 $$\begin{aligned} {} & \sum _{\begin{array}{c} d,d'\in [0, 4], \\ m\in [0,3] \end{array} } \delta _{\text  {dc}}(p,i,d,d',m)= 1, \quad p\in [1,s^*+ t^*], i\in [2, n_{\text  {tree}}^{\text  {S}}], \end{aligned}$$

70 $$\begin{aligned} {} & \sum _{\begin{array}{c} d,d'\in [0, 4], \\ m\in [0,3] \end{array}} m\cdot \delta _{\text  {dc}}(s,i,d,d',m) ={\widetilde{\beta }}(s,i), \quad s\in [1,s^*], i\in [2,n_{\text  {tree}}^{\text  {S}}], \end{aligned}$$

71 $$\begin{aligned} {} & \sum _{\begin{array}{c} d,d'\in [0, 4], \\ m\in [0,3] \end{array}} m\cdot \delta _{\text  {dc}}(s^*+ t,i,d,d',m) ={\widetilde{\beta }}(s^*+ t,i), \quad t\in [1,t^*], i\in [2,n_{\text  {tree}}^{\text  {T}}], \end{aligned}$$

72 $$\begin{aligned} {} & \sum _{\begin{array}{c} d\in [1,4],d'\in [0,4],\\ m\in [0,3] \end{array}} d \cdot \delta _{\text  {dc}}(p,i,d,d',m) =\deg (p,\text  {prt}(i)),  \\&\sum _{\begin{array}{c} d\in [0,4],d'\in [1,4],\\ m\in [0,3] \end{array}} d'\cdot \delta _{\text  {dc}}(t,i,d,d',m) =\deg (p, i), \quad p\in [1,s^*+ t^*], i\in [2,n_{\text  {tree}}^{\text  {S}}], \end{aligned}$$

73 $$\begin{aligned} {} & \sum _{d,d'\in [1, 4], m\in [0,3] } \delta _{\widehat{\text  {dc}}}(s,t,d,d',m)=1, \\&\sum _{d,d'\in [1, 4], m\in [0,3] } m\cdot \delta _{\widehat{\text  {dc}}}(s,t,d,d',m)={\widehat{\beta }}(s,t),\\&\sum _{d\in [1,4],d'\in [0,4], m\in [0,3]}d \cdot \delta _{\widehat{\text  {dc}}}(s,t,d,d',m) =\deg (s,1),  \\&\sum _{d\in [0,4],d'\in [1,4], m\in [0,3]} d' \cdot \delta _{\widehat{\text  {dc}}}(s,t,d,d',m) =\deg (s^*+ t,1),  \\&\quad s\in [1,s^*], t\in [1,t^*], \end{aligned}$$

74 $$\begin{aligned} {} & \sum _{i\in [1,c^*]}(\delta _{\text  {dc}}(i,d,d',m) + \delta _{\text  {dc}}(i,d',d,m)) \\&\quad + \sum _{t\in [2,t^*]}(\delta _{\text  {dc}}(t,1,d,d',m) + \delta _{\text  {dc}}(t,1,d',d,m) )  \\&\quad + \sum _{ s\in [1,s^*], t\in [1,t^*] } ( \delta _{\widehat{\text  {dc}}}(s,t,d,d',m) + \delta _{\widehat{\text  {dc}}}(s,t,d',d,m)) =\text  {bc}^{{\text  {in}}}(\mu ), \\&\sum _{ p\in [1,s^*+ t^*], i\in [2, n_{\text  {tree}}^{\text  {S}}] } ( \delta _{\text  {dc}}(p,i,d,d',m) + \delta _{\text  {dc}}(p,i,d',d,m) ) =\text  {bc}^\text  {ex}(\mu ), \\&\quad \mu =(d,d',m)\in {\text  {Bc}}, d<d', \end{aligned}$$

75 $$\begin{aligned} {} & \sum _{i\in [1,c^*]}\delta _{\text  {dc}}(i,d,d,m) + \sum _{t\in [2,t^*]}\delta _{\text  {dc}}(t,1,d,d,m) \nonumber \\&\quad + \sum _{ s\in [1,s^*], t\in [1,t^*] } \delta _{\widehat{\text  {dc}}}(s,t,d,d,m) =\text  {bc}^{\text  {in}}(\mu ), \nonumber \\&\sum _{ p\in [1,s^*+ t^*], i\in [2, n_{\text  {tree}}^{\text  {S}}] } \delta _{\text  {dc}}(p,i,d,d,m) =\text  {bc}^\text  {ex}(\mu ), \quad \mu =(d,d,m)\in \text  {Bc}. \end{aligned}$$

## Appendix D: Descriptions of new graph search algorithms

### Multi-rooted trees and frequency vectors

For a finite set *A* of elements, let ${\mathbb {Z}}_+^A$ denote the set of functions $\pmb {w}:A\rightarrow {\mathbb {Z}}_+$. A function $\pmb {w}\in {\mathbb {Z}}_+^A$ is called a *non-negative integer vector* (or a vector) on *A* and the value $\pmb {x}(a)$ for an element $a\in A$ is called the *entry* of $\pmb {x}$ for $a\in A$. For a vector $\pmb {w}\in {\mathbb {Z}}_+^A$ and an element $a\in A$, let $\pmb {w}+\pmb {1}_{a}$ (resp., $\pmb {w}-\pmb {1}_{a}$) denote the vector $\pmb {w}'$ such that $\pmb {w}'(a)=\pmb {w}(a)+1$ (resp., $\pmb {w}'(a)=\pmb {w}(a)-1$) and $\pmb {w}'(b)=\pmb {w}(b)$ for the other elements $b\in A\setminus \{a\}$. For a vector $\pmb {w}\in {\mathbb {Z}}_+^A$ and a subset $B\subseteq A$, let $\pmb {w}_{[B]}$ denote the *projection* of $\pmb {w}$ to *B*; i.e., $\pmb {w}_{[B]}\in {\mathbb {Z}}_+^B$ such that $\pmb {w}_{[B]}(b)=\pmb {w}(b)$, $b\in B$.

Let $\text  {Bc}$ denote the set of tuples $\mu =(d_1,d_2,k)\in [1,4]\times [1,4]\times [1,3]$ (bond-configuration) such that $\max \{d_1,d_2\}+k\le 4$. For two tuples $\mu =(d_1,d_2,k), \mu '=(d'_1,d'_2,k')\in \text  {Bc}$, we write $\mu \ge \mu '$ if

- $$\max \{d_1,d_2\}\ge \max \{d'_1,d'_2\}$$, $$\min \{d_1,d_2\}\ge \min \{d'_1,d'_2\}$$ and $$k\ge k'$$,

and write $\mu > \mu '$ if

- $$\mu \ge \mu '$$ and $$\mu \ne \mu '$$.

Let $\text  {Dg}=\{\text  {dg}1, \text  {dg}2, \text  {dg}3, \text  {dg}4\}$, where $\text  {dg}i$ denotes the number of vertices with degree *i*.

Henceforth we deal with vectors $\pmb {w}$ that have their $\pmb {w}_{\text  {in}}$ and $\pmb {w}_{\text  {ex}}$ components, both $\pmb {w}_{{\text  {in}}}, \pmb {w}_{\text  {ex}}\in {\mathbb {Z}}_+^{  {\Lambda \cup \Gamma \cup {\text  {Bc}}\cup {\text  {Dg}}}}$, and for convenience we write $\pmb {w}= (\pmb {w}_{\text  {in}}, \pmb {w}_\text  {ex})$ in the sense of concatenation.

For a vector $\pmb {x}= (\pmb {x}_{\text  {in}}, \pmb {x}_\text  {ex})$ with $\pmb {x}_{\text  {in}}, \pmb {x}_\text  {ex}\in {\mathbb {Z}}_+^{  {\Lambda \cup \Gamma \cup \text  {Bc}\cup \text  {Dg}}}$, let ${\mathcal {G}}(\pmb {x})$ denote the set of chemical acyclic graphs *G* whose 2-internal (resp., 2-external) vertices/edges are determined by the vector $\pmb {x}_{\text  {in}}$ (resp., $\pmb {x}_\text  {ex}$); i.e., *G* satisfies the following:

- $\text  {ce}_{\mathtt{a}}^{\text  {in}}(G)=\pmb {x}_{\text  {in}}({\mathtt{a}})$ and $\text  {ce}_{\mathtt{a}}^\text  {ex}(G)=\pmb {x}_\text  {ex}({\mathtt{a}})$ for each chemical element ${\mathtt{a}}\in \Lambda$,
- $\text  {ac}_{\gamma }^{\text  {in}}(G)= \pmb {x}_{\text  {in}}(\gamma )$ and $\text  {ac}_{\gamma }^\text  {ex}(G)=\pmb {x}_\text  {ex}(\gamma )$ for each adjacency-configuration $\gamma \in \Gamma$,
- $\text  {bc}_{\mu }^{\text  {in}}(G)=\pmb {x}_{\text  {in}}(\mu )$ and $\text  {bc}_{\mu }^\text  {ex}(G)=\pmb {x}_\text  {ex}(\mu )$ for each bond-configuration $\mu \in \text  {Bc}$,
- $\text  {dg}_i^{\text  {in}}(G)=\pmb {x}_{\text  {in}}(\text  {dg}i)$ and $\text  {dg}_i^\text  {ex}(G)=\pmb {x}_\text  {ex}(\text  {dg}i)$ for each degree $\text  {dg}i\in \text  {Dg}$.

Throughout the section, let $k^*=2$ be a branch-parameter, $\pmb {x}^*= (\pmb {x}_{\text  {in}}^*, \pmb {x}_\text  {ex}^*)$ be a given feature vector with $\pmb {x}_{\text  {in}}^*, \pmb {x}_\text  {ex}^*\in {\mathbb {Z}}_+^  {\Lambda \cup \Gamma \cup \text  {Bc}\cup \text  {Dg}}$, and $\text  {dia}^*$ be an integer. We infer a chemical acyclic graph $G\in {\mathcal {G}}(\pmb {x}^*)$ such that ${\text  {bl}}_2(G)\in [2,3]$ and the diameter of *G* is $\text  {dia}^*$, where $n^*=\sum _{{\mathtt{a}}\in \Lambda }(\pmb {x}^*_{\text  {in}}({\mathtt{a}})+\pmb {x}^*_\text  {ex}({\mathtt{a}}) )$. Note that any other descriptors of $G\in {\mathcal {G}}(\pmb {x}^*)$ can be determined by the entries of vector $\pmb {x}^*$.

To infer a chemical acyclic graph $G\in {\mathcal {G}}(\pmb {x}^*)$, we consider a connected subgraph *T* of *G* that consists of

76 $$\begin{aligned} \begin{array}{l} \text{- } \text{ a } \text{ subtree } \text{ of } \text{ the } \text{2-branch-subtree } G' \text{ of } G \text{ and } \\ \text{- } \text{ the } \text{2-fringe-trees } \text{ rooted } \text{ at } \text{ vertices } \text{ in } G'. \end{array} \end{aligned}$$

Our method first generates a set $\text  {FT}$ of all possible rooted trees *T* that can be a 2-fringe-tree of a chemical graph $G\in {\mathcal {G}}(\pmb {x}^*)$, and then extends the trees *T* by repeatedly appending a tree in $\text  {FT}$ until a chemical graph $G\in {\mathcal {G}}(\pmb {x}^*)$ is formed. In the extension, we actually manipulate the “frequency vectors” of trees defined below.

To specify which part of a given tree *T* plays the role of 2-internal vertices/edges or 2-external vertices/edges in a chemical graph $G\in {\mathcal {G}}(\pmb {x}^*)$ to be inferred, we designate at most three vertices $r_1(T)$, $r_2(T)$, and $r_3(T)$, in *T* as *terminals*, and call *T* *rooted* (resp., *bi-rooted* and *tri-rooted*) if the number of terminals is one (resp., two and three). For a rooted tree (resp., bi- or tri-rooted tree) *T*, let ${\widetilde{V}}_{{\text  {in}}}$ denote the set of vertices contained in a path between two terminals of *T*, ${\widetilde{E}}_{{\text  {in}}}$ denote the set of edges in *T* between two vertices in ${\widetilde{V}}_{{\text  {in}}}$, and define ${\widetilde{V}}_{\text  {ex}}\triangleq V(T)\setminus {\widetilde{V}}_{{\text  {in}}}$ and ${\widetilde{E}}_{\text  {ex}}\triangleq E(T)\setminus {\widetilde{E}}_{{\text  {in}}}$. For a bi- or tri-rooted tree *T*, define the *backbone path* $P_T$ of *T* to be the path of *T* between vertices $r_1(T)$ and $r_2(T)$.

Given a chemical acyclic graph *T*, define $\pmb {f}_\mathtt{t}(T)$, $\mathtt{t}\in \{{\text  {in}},\text  {ex}\}$, to be the vector $\pmb {w}\in {\mathbb {Z}}_+^{\Lambda \cup \Gamma \cup \text  {Bc}\cup \text  {Dg}}$ that consists of the following entries:

- $$\pmb {w}({\mathtt{a}})=|\{v\in {\widetilde{V}}_\mathtt{t} \mid \alpha (v)={\mathtt{a}}\}|$$, $${\mathtt{a}}\in \Lambda $$,
- $$\pmb {w}(\gamma )=|\{uv\in {\widetilde{E}}_\mathtt{t} \mid \{\alpha (u),\alpha (v)\}=\{\mathtt{a,b}\}, \beta (uv)=q\}|$$, $$\gamma =(\mathtt{a,b}, q)\in \Gamma $$,
- $$\pmb {w}(\mu )=|\{uv\in {\widetilde{E}}_\mathtt{t}\mid \{\deg _T(u),\deg _T(v)\}=\{ d,d'\}, \beta (uv)=m\}|$$, $$\mu =(d,d', m)\in \text  {Bc}$$,
- $$\pmb {w}(\text  {dg}i)=|\{v\in {\widetilde{V}}_\mathtt{t}\mid \deg _T(v)=i\}|$$, $$\text  {dg}i\in \text  {Dg}$$.

Define $\pmb {f}(T)\triangleq (\pmb {f}_{\text  {in}}(T),\pmb {f}_\text  {ex}(T))$. The entry for an element $\mathtt{e}\in   {\Lambda \cup \Gamma \cup \text  {Bc}\cup \text  {Dg}}$ in $\pmb {f}_\mathtt{t}(T)$, $\mathtt{t}\in \{{\text  {in}},\text  {ex}\}$ is denoted by $\pmb {f}_\mathtt{t}(\mathtt{e}; T)$. For a subset *B* of ${\Lambda \cup \Gamma \cup \text  {Bc}\cup \text  {Dg}}$, let $\pmb {f}_{\mathtt{t}[B]}(T)$ denote the projection of $\pmb {f}_\mathtt{t}(T)$ onto *B*.

Our aim is to generate all chemical bi-rooted (resp., tri-rooted) trees *T* with diameter $\text  {dia}^*$ such that $\pmb {f}(T)=\pmb {x}^*$.

### A new algorithm for computing chemical bi-rooted trees *G* with ${\text {bl}}_2(G)=2$

This section describes a sketch of our new graph search algorithm for the case of ${\text  {bl}}_2(G)=2$. See Appendix “[Sec Sec30]” for a sketch of a new algorithm for the case of ${\text  {bl}}_2(G)=3$.

We call a chemical graph $G\in {\mathcal {G}}(\pmb {x}^*)$ with diameter $\text  {dia}^*$ and ${\text  {bl}}_2(G)=2$ a *target graph*.

A chemical acyclic graph *G* with ${\text  {bl}}_2(G)=2$ has exactly two leaf 2-branches $v_i$, $i=1,2$, where the length of the path between the two leaf 2-branches $v_1$ and $v_2$ of a target graph *G* is $\text  {dia}^*-2k^*=\text  {dia}^*-4$. We observe that a connected subgraph *T* of a target graph *G* that satisfies (76) for ${\text  {bl}}_2(G)=2$ is a chemical rooted or bi-rooted tree with roots *u* and *v*, where possibly $u = v$. We call such a subgraph *T* an *internal-subtree* (resp., *end-subtree*) of *G* if neither (resp., one) of *u* and *v* is a 2-branch in *G*. When $u=v$, we call an internal-subtree (resp., end-subtree) *T* of *G* an *internal-fringe-tree* (resp., *end-fringe-tree*) of  *G*. Figure 10a–d illustrates an internal-subtree, an internal-fringe-tree, an end-subtree and an end-fringe-tree of *G*.

Let $\delta _1=\lfloor \frac{\text  {dia}^* - 5}{2} \rfloor$ and $\delta _2 =\text  {dia}^*-5-\delta _1=\lceil \frac{\text  {dia}^* - 5}{2} \rceil$. We regard a target graph $G\in {\mathcal {G}}(\pmb {x}^*)$ with ${\text  {bl}}_2(G)=2$ and diameter $\text  {dia}^*$ as a combination of two chemical bi-rooted trees $T_1$ and $T_2$ with $\ell (P_{T_i})=\delta _i$, $i=1,2$, joined by an edge $e=r_1(T_1)r_1(T_2)$, as illustrated in Fig. 11.

We start with generating chemical rooted trees and then iteratively extend chemical bi-rooted trees *T* with $\ell (P_T)=1,2,\dots ,\delta _1$, before we finally combine two chemical bi-rooted trees $T_1$ and $T_2$ with $\ell (P_{T_i})=\delta _i$. To describe our algorithm, we introduce some notation.

- Let ${\mathcal {T}}(\pmb {x}^*)$ denote the set of all bi-rooted trees *T* (where possibly $r_1(T)=r_2(T)$) such that $\pmb {f}_{{\text  {in}}}(T)\le \pmb {x}_{{\text  {in}}}^*$ and $\pmb {f}_{\text  {ex}}(T)\le \pmb {x}_{\text  {ex}}^*$, which is a necessary condition for *T* to be an internal-subtree or end-subtree of a target graph $G\in {\mathcal {G}}(\pmb {x}^*)$.
- Let ${\mathcal {FT}}$ denote the set of all rooted trees $T\in {\mathcal {T}}(\pmb {x}^*)$ that can be a 2-fringe-tree of a target graph *G*, where *T* satisfies the size constraint (1) of 2-fringe-trees.
- For each integer $h\in [1,\text  {dia}^*-4]$, let ${\mathcal {T}}_{\text  {end}}^{(h)}$ denote the set of all bi-rooted trees $T\in {\mathcal {T}}(\pmb {x}^*)$ that can be an end-subtree of a target graph *G* such that $\ell (P_T)=h$, and each 2-fringe-tree $T_v$ rooted at a vertex *v* in $P_T$ belongs to ${\mathcal {FT}}$.

The idea of our new algorithm is to compute only the set $\text  {W}_{\text  {end}}^{(h)}$ of frequency vectors $\pmb {w}$ of end trees, whose size $|\text  {W}_{\text  {end}}^{(h)}|$ is much more restricted than that of ${\mathcal {T}}_{\text  {end}}^{(h)}$. We compute the set $\text  {W}_{\text  {end}}^{(h)}$ of frequency vectors $\pmb {w}$ of trees in ${\mathcal {T}}_{\text  {end}}^{(h)}$ iteratively for each integer $h\ge 0$. During the computation, we keep a sample of a tree $T_{\pmb {w}}$ for each frequency vector $\pmb {w}$ so that a final step can construct some number of target graphs *G* by assembling these sample trees. Based on this, we generate target graphs $G\in {\mathcal {G}}(\pmb {x}^*)$ by the following steps:

1. (i) Compute ${\mathcal {FT}}$ by a branch-and-bound procedure that generates all possible rooted trees $T\in {\mathcal {T}}(\pmb {x}^*)$ (where $r_1(T)=r_2(T)$) that can be a 2-fringe-tree of a target graph $G\in {\mathcal {G}}(\pmb {x}^*)$;
  (ii) Compute the set $\text  {W}^{(0)}$ of all vectors $\pmb {w}=(\pmb {w}_{\text  {in}},\pmb {w}_\text  {ex})$ such that $\pmb {w}_{\text  {in}}=\pmb {f}_{{\text  {in}}}(T)$ and $\pmb {w}_\text  {ex}=\pmb {f}_{\text  {ex}}(T)$ for some tree $T\in {\mathcal {FT}}$, and let $\text  {W}_{\text  {end}}^{(0)} \subseteq \text  {W}^{(0)}$ be those trees with height exactly 2;
  (iii) For each vector $\pmb {w}=(\pmb {w}_{\text  {in}},\pmb {w}_\text  {ex})\in \text  {W}^{(0)}$, choose a sample tree $T_{\pmb {w}}\in {\mathcal {FT}}$ such that $\pmb {w}_{\text  {in}}=\pmb {f}_{{\text  {in}}}(T)$ and $\pmb {w}_\text  {ex}=\pmb {f}_{\text  {ex}}(T)$, and store these sample trees;
2. For each integer $h=1,2,\ldots , \delta _2$, iteratively execute the next:
  (i) Compute the set $\text  {W}_{\text  {end}}^{(h)}$ of all vectors $\pmb {w}=(\pmb {w}_{\text  {in}},\pmb {w}_\text  {ex})$ such that $\pmb {w}_{\text  {in}}=\pmb {f}_{{\text  {in}}}(T)$ and $\pmb {w}_\text  {ex}=\pmb {f}_{\text  {ex}}(T)$ for some bi-rooted tree $T\in {\mathcal {T}}_{\text  {end}}^{(h)}$, where such a vector $\pmb {w}$ is obtained from a combination of vectors $\pmb {w}'\in \text  {W}^{(0)}$ and $\pmb {w}''\in \text  {W}_{\text  {end}}^{(h-1)}$;
  (ii) For each vector $\pmb {w}\in \text  {W}_{\text  {end}}^{(h)}$, store a sample tree $T_{\pmb {w}}$, which is obtained from a combination of sample trees $T_{\pmb {w}'}$ with $\pmb {w}'\in \text  {W}^{(0)}$ and $T_{\pmb {w}''}$ with $\pmb {w}''\in \text  {W}_{\text  {end}}^{(h-1)}$;
3. We call a pair of vectors $\pmb {w}^1\in \text  {W}_{\text  {end}}^{(\delta _1)}$ and $\pmb {w}^2\in \text  {W}_{\text  {end}}^{(\delta _2)}$ *feasible*, if it admits a target graph $G\in {\mathcal {G}}(\pmb {x}^*)$ such that $\pmb {w}_{{\text  {in}}}^1+\pmb {w}_{{\text  {in}}}^2\le \pmb {x}^*_{{\text  {in}}}$ and $\pmb {w}_{\text  {ex}}^1+\pmb {w}_{\text  {ex}}^2\le \pmb {x}^*_{\text  {ex}}$. Find the set $\text  {W}_\text  {pair}$ of all feasible pairs of vectors $\pmb {w}^1$ and $\pmb {w}^2$;
4. For each feasible vector pair $(\pmb {w}^1,\pmb {w}^2)\in \text  {W}_\text  {pair}$, construct a corresponding target graph *G* by combining the corresponding samples trees $T_{\pmb {w}^1}$ and $T_{\pmb {w}^2}$, as illustrated in Fig. 11.

Detailed descriptions of the five steps in the above algorithm can be found in Appendix “Case of two leaf 2-branches”.

For a relatively large instance with $n^*\ge 40$ and $\text  {dia}^*\ge 20$, the number $|\text  {W}_\text  {pair}|$ of feasible vector pairs in Step 4 is still very large. In fact, the size $|\text  {W}_{\text  {end}}^{(h)}|$ of a vector set $\text  {W}_{\text  {end}}^{(h)}$ to be computed in Step 2 can also be considerably large during an execution of the algorithm. For such a case, we impose a time limitation on the running time for computing $\text  {W}_{\text  {end}}^{(h)}$ and a memory limitation on the number of vectors stored in a vector set $\text  {W}_{\text  {end}}^{(h)}$. With these limitations, we can compute only a limited subset ${\widehat{\text  {W}}}_{\text  {end}}^{(h)}$ of each vector set $\text  {W}_{\text  {end}}^{(h)}$ in Step 2. Even with such a subset ${\widehat{\text  {W}}}_{\text  {end}}^{(h)}$, we still can find a large size of a subset ${\widehat{\text  {W}}}_\text  {pair}$ of $\text  {W}_\text  {pair}$ in Step 3.

Our algorithm also delivers a lower bound on the number of all target graphs $G\in {\mathcal {G}}(\pmb {x}^*)$ in the following way. In Step 1, we also compute the number $t(\pmb {w})$ of trees $T\in {\mathcal {FT}}$ such that $\pmb {w}=\pmb {f}(T)$ for each $\pmb {w}\in \text  {W}^{(0)}$. In Step 2, when a vector $\pmb {w}$ is constructed from two vectors $\pmb {w}'$ and $\pmb {w}''$, we iteratively compute the number $t(\pmb {w})$ of trees *T* such that $\pmb {w}=\pmb {f}(T)$ by $t(\pmb {w}):=t(\pmb {w}')\times t(\pmb {w}'')$. In Step 3, when a feasible vector pair $(\pmb {w}^1,\pmb {w}^2)\in \text  {W}_\text  {pair}$ is obtained, we know that the number of the corresponding target graphs *G* is $t(\pmb {w}^1)\times t(\pmb {w}^2)$. Possibly we compute a subset ${\widehat{\text  {W}}}_\text  {pair}$ of $\text  {W}_\text  {pair}$ in Step 3. Then $(1/2)\sum _{(\pmb {w}^1,\pmb {w}^2)\in {\widehat{\text  {W}}}_\text  {pair}}t(\pmb {w}^1)\times t(\pmb {w}^2)$ gives a lower bound on the number of target graphs $G\in {\mathcal {G}}(\pmb {x}^*)$, where we divided by 2 since an axially symmetric target graph *G* can correspond to two vector pairs in $\text  {W}_\text  {pair}$.

### A sketch of algorithm for computing chemical tri-rooted trees *G* with ${\text {bl}}_2(G)=3$

We call a chemical graph $G\in {\mathcal {G}}(\pmb {x}^*)$ with diameter $\text  {dia}^*$ and ${\text  {bl}}_2(G)=3$ a *target graph*. Let $n^*_{\text  {inl}}\triangleq \sum _{{\mathtt{a}}\in \Lambda } \pmb {x}^*_{{\text  {in}}}({\mathtt{a}})$, which is the number of 2-internal vertices in a target graph $G\in {\mathcal {G}}(\pmb {x}^*)$.

A chemical acyclic graph *G* with ${\text  {bl}}_2(G)=3$ has exactly three leaf 2-branches $v_i$, $i=1, 2, 3$, and exactly one 2-internal vertex $v_4$ adjacent to three 2-internal vertices $v'_i$, $i=1, 2, 3$, as illustrated in Fig. 6(b). We call vertex $v_4$ the *joint-vertex* of *G*. Without loss of generality assume that the length of the path $P_{v_1,v_2}$ between $v_1$ and $v_2$ is $\text  {dia}^*-4$ and that the length of the path $P_{v_1,v'_1}$ is not smaller than that of $P_{v_2,v'_2}$.

Analogously with the case of ${\text  {bl}}_2(G)=2$, we define *internal-subtree* (resp., *end-subtree*, *internal-fringe-tree*, and *end-fringe-tree*) of *G*, to be a connected subgraph $G'$ that satisfies (76). Observe that *G* can be partitioned into three end-subtrees $T_i$, $i=1, 2, 3$, the 2-fringe-tree $T_4$ rooted at the joint-vertex $v_4$ and three edges $v'_i v_4$, $i=1,2,3$, where the backbone path $P_{T_i}$ connects leaf 2-branch $v_i$ and vertex $v'_i$. In particular, we call the end-subtree of *G* that consists of $T_1$, $T_2$, $T_4$, and edges $v'_i v_4$, $i=1,2$, the *main-subtree* of *G*, which consists of the path $P_{v_1, v_2}$ and all the 2-fringe-trees rooted at vertices in $P_{v_1, v_2}$. We call $T_3$ the *co-subtree* of *G*.

Let $\delta _i$, $i=1,2,3$ denote the length of the backbone path of $T_i$. Note that

- $$\delta _1+\delta _2+2=\text  {dia}^*-4$$ and $$ \delta _1\ge \delta _2\ge \delta _3= n^*_{\text  {inl}} - \text  {dia}^* + 2$$,

from which it follows that

- $$\delta _2 \in [\delta _3, \lfloor \text  {dia}^*/2 \rfloor -3 ] $$ and $$\delta _1 \in [\lceil \text  {dia}^*/2\rceil -3, \text  {dia}^* - 6 -\delta _3]$$.

We regard a target graph $G\in {\mathcal {G}}(\pmb {x}^*)$ with ${\text  {bl}}_2(G)=3$ and diameter $\text  {dia}^*$ as a combination of the main-subtree and the co-subtree joined with an edge. We represent the co-subtree as a chemical bi-rooted tree *T* with $\ell (P_T)=\delta _3$. We represent the main-subtree of a target graph *G* as a tri-rooted tree *T* with $\ell (P_T)=\text  {dia}-4$ so that terminals $r_1(T)$, $r_2(T)$, and $r_3(T)$, correspond to the two leaf 2-branches and the joint-vertex of *G*, respectively.

We start with generating chemical rooted trees and then iteratively extend chemical bi-rooted trees *T* with $\ell (P_T)=1, 2, \dots , \text  {dia}^*-6-\delta _3$, before we combine two chemical bi-rooted trees $T'$ and $T''$ to obtain a chemical tri-rooted tree $T_{1}$ with $\ell (P_{T_{1}})=\delta _i$, and finally, combine a chemical tri-rooted tree $T_{1}$ and a chemical bi-rooted tree $T_2$ with $\ell (P_{T_2})=\delta _3$, to obtain a target graph $G \in {\mathcal {G}}(\pmb {x}^*)$.

Analogously with the case of ${\text  {bl}}_2(G)=2$, we define the set ${\mathcal {T}}(\pmb {x}^*)$ of all bi-rooted trees *T*, the set ${\mathcal {FT}}$ of all rooted trees $T\in {\mathcal {T}}(\pmb {x}^*)$ that can be a 2-fringe-tree of a target graph *G* and the set ${\mathcal {T}}_{\text  {end}}^{(h)}$, $h\in [1, \text  {dia}^* -6 -\delta _3]$, of all bi-rooted trees $T\in {\mathcal {T}}(\pmb {x}^*)$ that can be an end-subtree of a target graph *G* such that $\ell (P_T)=h$.

We generate target graphs $G\in {\mathcal {G}}(\pmb {x}^*)$ by the following steps:

1. Analogously with Step 1 for the case of ${\text  {bl}}_2(G)=2$, compute the set ${\mathcal {FT}}$ by a branch-and-bound algorithm as described in "Step 1: Enumeration of 2-fringe-trees" section, and the set $\text  {W}^{(0)}$ of all vectors $\pmb {w}=(\pmb {w}_{\text  {in}},\pmb {w}_\text  {ex})$ such that $\pmb {w}_{\text  {in}}=\pmb {f}_{{\text  {in}}}(T)$ and $\pmb {w}_\text  {ex}=\pmb {f}_{\text  {ex}}(T)$ for some tree $T\in {\mathcal {FT}}$. For each vector $\pmb {w}\in \text  {W}^{(0)}$, store a sample tree $T_{\pmb {w}}\in {\mathcal {FT}}$, and let $\text  {W}_{\text  {end}}^{(0)} \subseteq \text  {W}^{(0)}$ be the set of feature vectors of possible end-trees with height 2;
2. For each integer $h=1,2,\ldots , \text  {dia}^* -6 -\delta _3$, compute the set $\text  {W}_{\text  {end}}^{(h)}$ of all vectors $\pmb {w}=(\pmb {w}_{\text  {in}},\pmb {w}_\text  {ex})$ such that $\pmb {w}_{\text  {in}}=\pmb {f}_{{\text  {in}}}(T)$ and $\pmb {w}_\text  {ex}=\pmb {f}_{\text  {ex}}(T)$ for some bi-rooted tree $T\in {\mathcal {T}}_{\text  {end}}^{(h)}$. For each vector $\pmb {w}\in \text  {W}_{\text  {end}}^{(h)}$, store a sample tree $T_{\pmb {w}}$;
3. For each integer $h\in [\lceil \text  {dia}^*/2\rceil -2, \text  {dia}^* - 5 -\delta _3]$, compute the set $\text  {W}_{\text  {end}+2}^{(h)}$ of all vectors $\pmb {w}=(\pmb {w}_{\text  {in}},\pmb {w}_\text  {ex})$ such that $\pmb {w}_{\text  {in}}=\pmb {f}_{{\text  {in}}}(T)$ and $\pmb {w}_\text  {ex}=\pmb {f}_{\text  {ex}}(T)$ of some bi-rooted tree *T* with $\ell (P_T)=h$ that represents an end-subtree rooted at the joint-vertex. For each vector $\pmb {w}\in \text  {W}_{\text  {end}+2}^{(h)}$, store a sample tree $T_{\pmb {w}}$;
4. For each integer $\delta _1 \in [\lceil \text  {dia}^*/2\rceil -3, \text  {dia}^* - 6 -\delta _3]$, compute the set $\text  {W}_{\text  {main}}^{(\delta _1+1)}$ of all vectors $\pmb {w}=(\pmb {w}_{\text  {in}},\pmb {w}_\text  {ex})$ such that $\pmb {w}_{\text  {in}}=\pmb {f}_{{\text  {in}}}(T)$ and $\pmb {w}_\text  {ex}=\pmb {f}_{\text  {ex}}(T)$ for some tri-rooted tree *T* that represents the main-subtree such that the length of the path $P_{r_2(T),r_3(T)}$ between terminals $r_2(T)$ and $r_3(T)$ is $\delta _1+1$. For each vector $\pmb {w}\in \text  {W}_{\text  {main}}^{(\delta _1+1)}$, store a sample tree $T_{\pmb {w}}$;
5. We call a pair of vectors $\pmb {w}^1\in \text  {W}_{\text  {main}}^{(\delta _1+1)}$ and $\pmb {w}^2\in \text  {W}_{\text  {end}}^{(\delta _3)}$ *feasible* if it admits a target graph $G\in {\mathcal {G}}(\pmb {x}^*)$ such that $\pmb {w}_{{\text  {in}}}^1+\pmb {w}_{{\text  {in}}}^2\le \pmb {x}^*_{{\text  {in}}}$ and $\pmb {w}_{\text  {ex}}^1+\pmb {w}_{\text  {ex}}^2\le \pmb {x}^*_{\text  {ex}}$. Find the set $\text  {W}_\text  {pair}$ of all feasible pairs of vectors $\pmb {w}^1$ and $\pmb {w}^2$;
6. For each feasible vector pair $(\pmb {w}^1,\pmb {w}^2)\in \text  {W}_\text  {pair}$, construct a corresponding target graph *G* by combining the samples trees $T_{\pmb {w}^1}$ and $T_{\pmb {w}^2}$, which correspond to the main-subtree and the co-subtree of a target graph *G*, respectively, as illustrated in Fig. 12.

Detailed descriptions of the six steps in the above algorithm can be found in Appendix “Case of three leaf 2-branches”.

### Frequency vectors of fictitious trees

Let *T* be a chemical bi-rooted or tri-rooted tree, where we regard a rooted tree *T* as a bi-rooted tree with $r_1(T)=r_2(T)$ for a notational convenience. Recall that our algorithm generates a target graph $G\in {\mathcal {G}}(\pmb {x}^*)$ as a supergraph of *T*, where one of terminals $r_1(T)$ and $r_2(T)$ can be a 2-branch of *G*. We assume that the second terminal $r_2(T)$ will be a 2-branch of *G* in such a case in our algorithms.

For an integer $p\in [1,3]$, let $T[+p]$ denote a fictitious chemical graph obtained from *T* by regarding the degree of terminal $r_1(T)$ as $\deg _T(r_1(T))+p$. Figure 13 (resp., Fig. 14a) illustrates fictitious trees $T[+p]$ in the case of $r_1(T)=r_2(T)$ (resp., $r_1(T)\ne r_2(T)$). The frequency vectors $\pmb {f}_{{\text  {in}}}(T[+p])$ and $\pmb {f}_{\text  {ex}}(T[+p])$ are obtained as follows: Let $d=\deg _T(r_1(T))$, $v_i$, $i\in [1,d]$, denote the neighbors of $r_1(T)$, and $d_i=\deg _T(v_i)$, $m_i=\beta (r_1(T) v_i)$, and $\mu _i=(d, d_i, m_i)$, $\mu '_i=(d+p, d_i, m_i)$, $i\in [1,d]$.

For $$r_1(T)=r_2(T)$$ and $$d'=d+p$$,

- $$\pmb {f}_{{\text  {in}}}(T[+p])= \pmb {f}_{{\text  {in}}}(T) +\pmb {1}_{\text  {dg}d'}-\pmb {1}_{\text  {dg}d}$$,   $$\displaystyle { \pmb {f}_{\text  {ex}}(T[+p])= \pmb {f}_{\text  {ex}}(T) + \sum _{1\le i\le d}( \pmb {1}_{\mu '_i} -\pmb {1}_{\mu _i}) }$$.

For $r_1(T)\ne r_2(T)$ and $d'=d+p$, where $v_d$ denotes the vertex in $P_T$,

- $$\pmb {f}_{{\text  {in}}}(T[+1]) = \pmb {f}_{{\text  {in}}}(T) +\pmb {1}_{\text  {dg}d'}-\pmb {1}_{\text  {dg}d} +\pmb {1}_{\mu '_d}-\pmb {1}_{\mu _d}$$,
- $$\displaystyle { \pmb {f}_{\text  {ex}}(T[+1]) = \pmb {f}_{\text  {ex}}(T) +\sum _{1\le i\le d-1}(\pmb {1}_{\mu '_i}-\pmb {1}_{\mu _i}) }$$.

Let *T* be a chemical tri-rooted tree, where the third terminal $r_3(T)$ is in the backbone path $P_T$ between vertices $r_1(T)$ and $r_2(T)$. Let $T\langle +1\rangle$ denote a fictitious chemical graph obtained from *T* by regarding the degree of terminal $r_3(T)$ as $\deg _T(r_3(T))+1$. Figure 14b illustrates a fictitious tri-rooted tree $T\langle +1\rangle$. The frequency vectors $\pmb {f}_{{\text  {in}}}(T\langle +1\rangle )$ and $\pmb {f}_{\text  {ex}}(T\langle +1\rangle )$ are obtained as follows: Let $d=\deg _T(r_3(T))$, $v_i$, $i\in [1,d]$, denote the neighbors of $r_3(T)$, where $v_{d-1}$ and $v_{d}$ are contained in the path $P_T$. For each index $i\in [1,d]$, let $d_i=\deg _T(v_i)$, $m_i=\beta (r_3(T) v_i)$, $\mu _i=(d, d_i, m_i)$, and $\mu '_i=(d+1, d_i, m_i)$.

Then

77 $$\begin{aligned} \pmb {f}_{{\text  {in}}}(T\langle +1\rangle )&= \pmb {f}_{{\text  {in}}}(T) +\pmb {1}_{\text  {dg}(d+1)}-\pmb {1}_{\text  {dg}d}+ \sum _{ i\in [ d-1,d]}( \pmb {1}_{\mu '_i} -\pmb {1}_{\mu _i}) , \nonumber \\ \pmb {f}_{\text  {ex}}(T\langle +1\rangle )&= \pmb {f}_{\text  {ex}}(T) + \sum _{ i\le [1,d-2]}( \pmb {1}_{\mu '_i} -\pmb {1}_{\mu _i}) . \end{aligned}$$

### Sets of frequency vectors

For an element ${\mathtt{a}}\in \Lambda$ and integers $d \in [0, d_\text  {max}-2]$ and $m\in [d,{\text  {val}}({\mathtt{a}})-1]$, let $\text  {W}_{\text  {inl}}^{(0)}({\mathtt{a}}, d, m)$ (resp., $\text  {W}_{\text  {inl}+3}^{(0)}({\mathtt{a}}, d, m)$) denote the set of frequency vectors $(\pmb {f}_{{\text  {in}}}(T[+2]), \pmb {f}_{\text  {ex}}(T[+2]))$ (resp., $(\pmb {f}_{{\text  {in}}}(T[+3]), \pmb {f}_{\text  {ex}}(T[+3]))$) of a chemical rooted tree *T* such that

- $r_1(T)=r_2(T)$, the height of *T* is at most 2,
- $$\alpha (r_1(T))={\mathtt{a}}$$, $$\deg _T(r_1(T))=d$$, and $$\beta (r_1(T))=m$$.

Recall that $\beta (u)=\sum _{uv\in E}\beta (uv)$, defined in “Preliminary” section.

For an element ${\mathtt{a}}\in \Lambda$ and integers $d \in [1, d_\text  {max}-1]$, $m\in [d,{\text  {val}}({\mathtt{a}})-1]$, and $h\ge 0$, let $\text  {W}_{\text  {end}}^{(h)}({\mathtt{a}}, d, m)$ (resp., $\text  {W}_{\text  {end}+2}^{(h)}({\mathtt{a}}, d, m)$) denote the set of frequency vectors $(\pmb {f}_{{\text  {in}}}(T[+1]), \pmb {f}_{\text  {ex}}(T[+1]))$ (resp., $(\pmb {f}_{{\text  {in}}}(T[+2]), \pmb {f}_{\text  {ex}}(T[+2]))$) of chemical bi-rooted trees *T* such that

- $$\alpha (r_1(T))={\mathtt{a}}$$, $$\deg _T(r_1(T))=d$$, $$\beta (r_1(T))=m$$, $$\ell (P_T)=h$$ and
- if $h=0$ then the height of the tree $T'$ rooted at $r_2(T)$ is 2.

### Case of two leaf 2-branches

#### Step 1: Enumeration of 2-fringe-trees

The main task of Step 1 is to compute for each tuple $({\mathtt{a}},d,m)$ of an element ${\mathtt{a}}\in \Lambda$ and integers $d \in [1,d_\text  {max}-1]$ (resp., $d\in [0,d_\text  {max}-2]$) and $m\in [d,{\text  {val}}({\mathtt{a}})-1]$ (resp., $m\in [d,{\text  {val}}({\mathtt{a}})-2]$), the set $\text  {W}_{\text  {end}}^{(0)}({\mathtt{a}}, d, m)$ (resp., $\text  {W}_{\text  {inl}}^{(0)}({\mathtt{a}}, d, m)$) of all frequency vectors $\pmb {f}(T[+1])$ (resp., $\pmb {f}(T[+2])$) of chemical rooted trees *T* such that $r_1(T)=r_2(T)$, $\alpha (r_1(T))={\mathtt{a}}$, $\deg _T(r_1(T))=d$ and $\beta (r_1(T))=m$.

Step 1 first computes the set ${\mathcal {FT}}$ of all possible chemical rooted trees $T\in {\mathcal {T}}(\pmb {x}^*)$ (where $r_1(T)=r_2(T)$) that can be a 2-fringe-tree of a target graph $G\in {\mathcal {G}}(\pmb {x}^*)$. For this, we design a branch-and-bound procedure where we append a new vertex one by one to construct a rooted tree with only one child. To design a bounding procedure, we derive a property of the structure of chemical rooted trees that can be a 2-fringe-tree of a target graph.

Let $G_0$ be a chemical rooted tree with a terminal $r_0=r_1(G_0)=r_2(G_0)$, where $\pmb {f}_{{\text  {in}}}(\alpha (r_0);G_0)=1$ and $\pmb {f}_{{\text  {in}}}({\mathtt{a}};G_0)=0$, ${\mathtt{a}}\in \Lambda \setminus \{\alpha (r_0)\}$ and $\pmb {f}_{{\text  {in}}}(\gamma ;G_0)=0$, $\gamma \in \Gamma$. For a vector $\pmb {x}= (\pmb {x}_{\text  {in}}, \pmb {x}_\text  {ex})$ with $\pmb {x}_{\text  {in}}, \pmb {x}_\text  {ex}\in {\mathbb {Z}}_+^{  {\Lambda \cup \Gamma \cup \text  {Bc}\cup \text  {Dg}}}$, we call $G_0$ $\pmb {x}$-*extensible* if some chemical acyclic graph $G\in {\mathcal {G}}(\pmb {x})$ contains $G_0$ as a subgraph of a 2-fringe-tree *T* rooted at $r_0$ in *G*.

We use the next condition as a bounding procedure when we generate chemical rooted trees in Step 1.

##### **Lemma 3**

*For a branch-parameter*$k=2$*, let*$\pmb {x}^* = (\pmb {x}_{\text  {in}}^*, \pmb {x}_\text  {ex}^*)$*be a vector with*$\pmb {x}_{\text  {in}}^*, \pmb {x}_\text  {ex}^*\in {\mathbb {Z}}_+^{{\Lambda \cup \Gamma \cup \text  {Bc}\cup \text  {Dg}}}$*, and*$G_0$*be a chemical rooted tree rooted at a vertex*$r_0$*such that*$\pmb {f}(G_0)\le \pmb {x}^*$.

(i) Graph $G_0$ is $\pmb {x}^*$-extensible only when the next holds for any subset $\Lambda '\subseteq \Lambda$:
  78 $$\begin{aligned} \sum _{{\mathtt{a}}\in \Lambda '} (\pmb {x}_\text  {ex}^*({\mathtt{a}}) - \pmb {f}_\text  {ex}({\mathtt{a}}; G_0))&\le \sum _{\begin{array}{c} \gamma =(\mathtt{a,b},m)\in \Gamma : \\ {\mathtt{a}}\in \Lambda ', \mathtt{b}\in \Lambda \setminus \Lambda ' \end{array}} (\pmb {x}_\text  {ex}^*(\gamma ) - \pmb {f}_\text  {ex}(\gamma ; G_0)) \nonumber \\&\quad + 2 \sum _{\begin{array}{c} \gamma =(\mathtt{a,b},m)\in \Gamma : \\ \mathtt{a,b}\in \Lambda ' \end{array}} (\pmb {x}_\text  {ex}^*(\gamma ) - \pmb {f}_\text  {ex}(\gamma ; G_0)). \end{aligned}$$
(ii) Let $G_1$ denote the chemical rooted tree obtained from $G_0$ by appending a new atom with an element $\mathtt{b}\in \Lambda$ to an atom with an element ${\mathtt{a}}\in \Lambda$ in $G_0$ with a multiplicity *q*; i.e., we join an atom ${\mathtt{a}}$ in $G_0$ and a new atom $\mathtt{b}$ with an adjacency-configuration $(\mathtt{a,b},q)$. Then $G_1$ is $\pmb {x}^*$-extensible only when the next holds:
  - $$\pmb {x}_\text  {ex}^*({\mathtt{a}}) - \pmb {f}_\text  {ex}({\mathtt{a}}; G_0)\le \pmb {nb}({\mathtt{a}}) -1$$
  for
  - $$\pmb {nb}({\mathtt{a}})= \displaystyle { \sum _{\begin{array}{c} \gamma =(\mathtt{a,b},m)\in \Gamma : \\ \mathtt{b\ne a}\in \Lambda \end{array} } (\pmb {x}_\text  {ex}^*(\gamma ) - \pmb {f}_\text  {ex}(\gamma ; G_0) ) + 2 \sum _{\gamma =(\mathtt{a,a},m)\in \Gamma } (\pmb {x}_\text  {ex}^*(\gamma ) - \pmb {f}_\text  {ex}(\gamma ; G_0) ) }$$.

##### *Proof*

(i) Assume that $G_0$ is a subgraph of a 2-fringe-tree *T* in some chemical graph $G\in {\mathcal {G}}(\pmb {x}^*)$ so that *T* is rooted at $r_0$. The left-hand side means the number of the remaining 2-external vertices with elements in $\Lambda '$ in the 2-fringe-trees in *G*. Each of such atoms has a neighbor in the connected graph *G*. The right-hand side indicates an upper bound on the number of 2-external edges joining elements in $\Lambda '$ in the 2-fringe-trees in *G*.
(ii) Note that $\pmb {f}_{\text  {ex}[\Lambda \cup \Gamma ]}(G_1) = \pmb {f}_{\text  {ex}[\Lambda \cup \Gamma ]}(G_0)+\pmb {1}_\mathtt{b}+\pmb {1}_{\gamma }$. For $\Lambda '=\{{\mathtt{a}}\}$, the left-hand side in Eq. (78) is $\pmb {x}^*_\text  {ex}({\mathtt{a}})- \pmb {f}_\text  {ex}({\mathtt{a}}; G_0)$, which remains unchanged if $\mathtt{a\ne b}$ (resp., is reduced by 1 if $\mathtt{a= b}$); and the right-hand side in (78) is $\pmb {nb}({\mathtt{a}})$, which is reduced by 1 if $\mathtt{a\ne b}$ (resp., is reduced by 2 if $\mathtt{a= b}$). That is, the left-hand side minus the right-hand side in (78) is always reduced by 1. This gives the required necessary condition for $G_1$ to be $\pmb {x}^*$-extensible.

$$\square $$

Figure 15 illustrates all graph structures of rooted trees *T* with height at most 2 and only one child satisfying the size constraint (1). For each element ${\mathtt{a}}\in \Lambda$, we enumerate chemical trees $T\in {\mathcal {T}}(\pmb {x}^*)$ rooted at vertex *r* with $\alpha (r)={\mathtt{a}}$ that has only one child by a branch-and-bound algorithm. Let ${\mathcal {T}}_{\mathtt{a}}$ denote the set of resulting rooted trees for each root element ${\mathtt{a}}\in \Lambda$.

We next enumerate chemical trees $T\in {\mathcal {T}}(\pmb {x}^*)$ rooted at vertex *r* with $\alpha (r)={\mathtt{a}}$ that has two or three children by generating a combination of two or three graphs in ${\mathcal {T}}_{\mathtt{a}}$. During generating graphs, our bounding procedure tests whether the current graph satisfies the necessary condition in Lemma 3(ii).

Finally, we compute the following sets:

for each element ${\mathtt{a}}\in \Lambda$, integers $d\in [1,d_\text  {max}-1]$, $m\in [d,{\text  {val}}({\mathtt{a}})-1]$, the set $\text  {W}_{\text  {end}}^{(0)}({\mathtt{a}}, d, m)$ of frequency vectors $\pmb {f}(T[+1])$ for rooted trees $T\in {\mathcal {T}}_{\mathtt{a}}$ with $\deg _T(r)=d$ and height 2;

for each element ${\mathtt{a}}\in \Lambda$, integers $d\in [0,d_\text  {max}-2]$, $m\in [d,{\text  {val}}({\mathtt{a}})-2]$, the set $\text  {W}_{\text  {inl}}^{(0)}({\mathtt{a}}, d, m)$ of frequency vectors $\pmb {f}(T[+2])$ for rooted trees $T\in {\mathcal {T}}_{\mathtt{a}}$ with $\deg _T(r)=d$ and height at most 2.

For each vector $\pmb {w}\in \text  {W}_{\text  {end}}^{(0)}({\mathtt{a}}, d, m)$ (resp., $\pmb {w}\in \text  {W}_{\text  {inl}}^{(0)}({\mathtt{a}}, d, m)$), we store a sample tree $T_{\pmb {w}}$.

We remark that the size of the set ${\mathcal {FT}}$ depends on the vector $\pmb {x}^*$. However, since the height of trees is limited to 2, the degree is at most 3 or 4, and the size constraint (1) on fringe trees in "Our target graph class" section, the size of the set ${\mathcal {FT}}$ is fairly limited.

#### Step 2: Generation of frequency vectors of end-subtrees

The main task of Step 2 is to compute the following sets in the ascending order of $h=1,2,\ldots , \delta _2$:

For elements ${\mathtt{a}}\in \Lambda$, integers $d \in [1,d_\text  {max}-1]$, $m \in [d ,{\text  {val}}({\mathtt{a}})-1]$, and $h\in [ 1, \delta _2 ]$, the sets $\text  {W}_{\text  {end}}^{(h)}({\mathtt{a}}, d , m )$ of all frequency vectors $\pmb {f}(T[+1])$ of chemical bi-rooted trees $T\in {\mathcal {T}}(\pmb {x}^*)$ such that $\alpha (r_1(T))={\mathtt{a}}$, $\deg _T(r_1(T))=d$, $\beta (r_1(T))=m$ and $\ell (P_T)=h$.

Observe that each vector $\pmb {w}=(\pmb {w}_{{\text  {in}}},\pmb {w}_{\text  {ex}})\in \text  {W}_{\text  {end}}^{(h)}({\mathtt{a}}, d , m )$ is obtained from a combination of vectors $\pmb {w}'=(\pmb {w}'_{{\text  {in}}},\pmb {w}'_{\text  {ex}})\in \text  {W}_{\text  {inl}}^{(0)}({\mathtt{a}}, d -1, m')$ and $\pmb {w}''=(\pmb {w}''_{{\text  {in}}},\pmb {w}''_{\text  {ex}}) \in \text  {W}_{\text  {end}}^{(h-1)}(\mathtt{b}, d'', m'')$ such that

- $$m' \le {\text  {val}}({\mathtt{a}}) - 2$$, $$1\le m - m' \le {\text  {val}}(b) -m'' $$,
- $$\pmb {w}_{{\text  {in}}} = \pmb {w}'_{{\text  {in}}} + \pmb {w}''_{{\text  {in}}} + \pmb {1}_\gamma + \pmb {1}_\mu \le \pmb {x}_{{\text  {in}}}^*$$, $$\pmb {w}_{\text  {ex}} = \pmb {w}'_{\text  {ex}} + \pmb {w}''_{\text  {ex}} \le \pmb {x}_{\text  {ex}}^*$$
- for $$\gamma = ({\mathtt{a}}, \mathtt{b}, m - m' ) \in \Gamma $$ and $$\mu = (d+1, d''+1, m - m') \in \text  {Bc}$$.

Figure 16 illustrates this process of computing a vector $\pmb {w}\in \text  {W}_{\text  {end}}^{(h)}({\mathtt{a}}, d , m )$.

For each vector $\pmb {w}\in \text  {W}_{\text  {end}}^{(h)}({\mathtt{a}}, d , m )$ obtained from a combination $\pmb {w}'\in \text  {W}_{\text  {inl}}^{(0)}({\mathtt{a}}, d -1, m')$ and $\pmb {w}'' \in \text  {W}_{\text  {end}}^{(h-1)}(\mathtt{b}, d'', m'')$, we construct a sample tree $T_{\pmb {w}}$ from their sample trees $T_{\pmb {w}'}$ and $T_{\pmb {w}''}$.

#### Step 3: Enumeration of feasible vector pairs

A *feasible pair* of vectors is defined to be a pair of vectors $\pmb {w}^i= (\pmb {w}^i_{{\text  {in}}}, \pmb {w}^i_{\text  {ex}}) \in \text  {W}_{\text  {end}}^{(\delta _i)} ({\mathtt{a}}_i,d_i,m_i)$, ${\mathtt{a}}_i\in \Lambda$, $d_i\in [1,d_\text  {max}-1]$, $m_i\in [d_i,{\text  {val}}({\mathtt{a}}_i)-1]$, $i = 1,2$ that admits an adjacency-configuration $\gamma = ({\mathtt{a}}_1, {\mathtt{a}}_2, m) \in \Gamma$ and a bond-configuration $\mu = (d_1 + 1, d_2 + 1, m) \in \text  {Bc}$ with an integer $m \in [1, \min \{3, {\text  {val}}({\mathtt{a}}_1) - m_1, {\text  {val}}({\mathtt{a}}_2) - m_2\} ]$ such that

- $$\pmb {x}^*_{{\text  {in}}} = \pmb {w}^1_{{\text  {in}}} + \pmb {w}^2_{{\text  {in}}} + \pmb {1}_{\gamma } + \pmb {1}_{\mu }$$ and $$\pmb {x}^*_{\text  {ex}} = \pmb {w}^1_{\text  {ex}} + \pmb {w}^2_{\text  {ex}}$$,

or equivalently $\pmb {w}^1$ is equal to the vector $(\pmb {x}^*_{{\text  {in}}} - \pmb {w}^2_{{\text  {in}}}-\pmb {1}_{\gamma } -\pmb {1}_{\mu }, \pmb {x}^*_{\text  {ex}} - \pmb {w}^1_{\text  {ex}})$, which we call the $(\gamma , \mu )$-*complement* of $\pmb {w}^2$, and denote it by $\overline{\pmb {w}^2}$.

The main task of Step 3 is to enumerate all feasible vector pairs $(\pmb {w}^1,\pmb {w}^2)$, $\pmb {w}^i \in \text  {W}_{\text  {end}}^{(\delta _i)}({\mathtt{a}}_i,d_i,m_i)$ with ${\mathtt{a}}_i\in \Lambda$, $d_i\in [1,d_\text  {max}-1]$, $m_i\in [d_i,{\text  {val}}({\mathtt{a}}_i)-1]$, $i = 1,2$.

To efficiently search for a feasible pair of vectors in two sets $\text  {W}_{\text  {end}}^{(\delta _i)}({\mathtt{a}}_i,d_i,m_i)$, $i = 1,2$, we first compute the $(\gamma , \mu )$-complement vector $\overline{\pmb {w}}$ of each vector $\pmb {w}\in \text  {W}_{\text  {end}}^{(\delta _2)}({\mathtt{a}}_2,d_2,m_2)$ for each pair of $\gamma = ({\mathtt{a}}_1, {\mathtt{a}}_2, m) \in \Gamma$ and $\mu = (d_1 + 1, d_2 + 1, m) \in \text  {Bc}$ with $m \in [1, \min \{3, {\text  {val}}({\mathtt{a}}_1) - m_1, {\text  {val}}({\mathtt{a}}_2) - m_2\} ]$, and denote by $\overline{\text  {W}_{\text  {end}}^{(\delta _2)}}$ the set of the resulting $(\gamma , \mu )$-complement vectors. Observe that $(\pmb {w}^1,\pmb {w}^2)$ is a feasible vector pair if and only if $\pmb {w}_1=\overline{\pmb {w}_2}$. To find such pairs, we merge the sets $\text  {W}_{\text  {end}}^{(\delta _1)}({\mathtt{a}}_1,d_1,m_1)$ and $\overline{\text  {W}_{\text  {end}}^{(\delta _2)}}$ into a sorted list $L_{\gamma , \mu }$. Then each feasible vector pair $(\pmb {w}^1,\pmb {w}^2)$ appears as a consecutive pair of vectors $\pmb {w}_1$ and $\overline{\pmb {w}_2}$ in the list $L_{\gamma , \mu }$.

#### Step 4: Construction of chemical graphs

The task of Step 4 is to construct for each feasible vector pair $\pmb {w}^i\in \text  {W}_{\text  {end}}^{(\delta _i)}({\mathtt{a}}_i,d_i,m_i)$, $i = 1,2$ such that $\pmb {w}^1$ is equal to the $(\gamma = ({\mathtt{a}}_1, {\mathtt{a}}_2, m), \mu )$-complement vector $\overline{\pmb {w}^2}$ of $\pmb {w}^2$, construct a target graph $T_{(\pmb {w}_1, \pmb {w}_2)}\in {\mathcal {G}}(\pmb {x}^*)$ by combining the sample trees $T_i=T_{\pmb {w}^i}$ of vectors $\pmb {w}^i$ with an edge $e=r_1(T_1)r_1(T_2)$ such that $\beta (e)=m$. Figure 11 illustrates two sample trees $T_i$, $i=1,2$ to be combined with a new edge $e=r_1(T_1)r_1(T_2)$.

### Case of three leaf 2-branches

#### Step 1: Enumeration of 2-fringe-trees

The main task of Step 1 is to compute the following sets:

for each tuple $({\mathtt{a}},d,m)$ of an element ${\mathtt{a}}\in \Lambda$ and integers $d \in [1,d_\text  {max}-1]$ (resp., $d\in [0,d_\text  {max}-2]$ and $d\in [0,d_\text  {max}-3]$) and $m\in [d,{\text  {val}}({\mathtt{a}})-1]$ (resp., $m\in [d,{\text  {val}}({\mathtt{a}})-2]$ and $m\in [d,{\text  {val}}({\mathtt{a}})-3]$), the set $\text  {W}_{\text  {end}}^{(0)}({\mathtt{a}}, d, m)$ (resp., $\text  {W}_{\text  {inl}}^{(0)}({\mathtt{a}}, d, m)$ and $\text  {W}_{\text  {inl}+3}^{(0)}({\mathtt{a}}, d, m)$) of all frequency vectors $\pmb {f}(T[+1])$ (resp., $\pmb {f}(T[+2])$ and $\pmb {f}(T[+3])$) of chemical rooted trees *T* such that $r_1(T)=r_2(T)$, $\alpha (r_1(T))={\mathtt{a}}$, $\deg _T(r_1(T))=d$ and $\beta (r_1(T))=m$. For each vector $\pmb {w}\in \text  {W}_{\text  {end}}^{(0)}({\mathtt{a}}, d, m)$ (resp., $\pmb {w}\in \text  {W}_{\text  {inl}}^{(0)}({\mathtt{a}}, d, m)$ and $\pmb {w}\in \text  {W}_{\text  {inl}+3}^{(0)}({\mathtt{a}}, d, m)$), we store a sample tree $T_{\pmb {w}}$. This step can be designed in a similar way as Step 1 for the case of ${\text  {bl}}_2(G)=2$.

#### Step 2: Generation of frequency vectors of end-subtrees

Analogously with Step 2 for the case of ${\text  {bl}}_2(G)=2$, Step 2 computes the following sets in the ascending order of $h=1,2,\ldots , \text  {dia}^* -6 -\delta _3$:

For elements ${\mathtt{a}}\in \Lambda$, integers $d \in [1,d_\text  {max}-1]$, $m \in [d ,{\text  {val}}({\mathtt{a}})-1]$, $i=1,2$, and $h\in [ 1, \text  {dia}^* -6 -\delta _3]$, the sets $\text  {W}_{\text  {end}}^{(h)}({\mathtt{a}},d,m)$ of all frequency vectors $\pmb {f}(T[+1])$ of chemical bi-rooted trees $T\in {\mathcal {T}}(\pmb {x}^*)$ such that $\alpha (r_1(T))={\mathtt{a}}$, $\deg _T(r_1(T))=d$, $\beta (r_1(T))=m$ and $\ell (P_T)=h$.

For each vector $\pmb {w}\in \text  {W}_{\text  {end}}^{(h)}({\mathtt{a}}, d, m)$, we construct a sample tree $T_{\pmb {w}}$ from their sample trees $T_{\pmb {w}'}$ and $T_{\pmb {w}''}$.

#### Step 3: Generation of frequency vectors of end-subtrees with two fictitious edges

The main task of Step 3 is to compute the following sets:

For elements ${\mathtt{a}}\in \Lambda$, integers $d \in [1,d_\text  {max}-2]$, $m\in [d,{\text  {val}}({\mathtt{a}})-2]$ and $h\in [\lceil \text  {dia}^*/2\rceil -2, \text  {dia}^* - 5 -\delta _3]$, the sets $\text  {W}_{\text  {end}+2}^{(h)}({\mathtt{a}},d,m)$ of all frequency vectors of bi-rooted trees $T[+2]$ such that $\alpha (r_1(T))={\mathtt{a}}$, $\deg _T(r_1(T))=d$, $\beta (r_1(T))=m$ and $\ell (P_T)=h$. For each vector $\pmb {w}\in \text  {W}_{\text  {end}+2}^{(h)}({\mathtt{a}},d,m)$, we store a sample tree $T_{\pmb {w}}$. This step can be designed in a similar way as Step 3 for the case of ${\text  {bl}}_2(G)=2$.

#### Step 4: Enumeration of frequency vectors of main-subtrees

For an element ${\mathtt{a}}\in \Lambda$, and integers $d \in [2,d_\text  {max}-1]$, $m \in [d, {\text  {val}}({\mathtt{a}}) -1]$, and $\delta _1 \in [\lceil \text  {dia}^*/2\rceil -3, \text  {dia}^* - 6 -\delta _3]$, define $\text  {W}_{\text  {main}}^{(\delta _1+1)}({\mathtt{a}}, d, m)$ to be the set of the frequency vectors $\pmb {f}(T\langle +1\rangle )$ of chemical tri-rooted trees *T* such that

- $$\alpha (r_1(T))={\mathtt{a}}$$, $$\deg _T(r_1(T))=d$$, $$\beta (r_1(T))=m$$, $$\ell (P_T)=\text  {dia}^*-4$$ and
- the length of the path $P_{r_2(T),r_3(T)}$ between vertices $r_2(T)$ and $r_3(T)$ is $\delta _1+1$.

See Fig. 12 for the structure of a main-tree. Such a chemical tri-rooted graph *T* corresponds to the main-subtree of a target graph $G\in {\mathcal {G}}(\pmb {x}^*)$.

The main task of Step 4 is to compute the sets $\text  {W}_{\text  {main}}^{(\delta _1+1)}({\mathtt{a}}, d, m)$, ${\mathtt{a}}\in \Lambda$, $d \in [2,d_\text  {max}-1]$, $m \in [d, {\text  {val}}({\mathtt{a}}) -1]$, $\delta _1 \in [\lceil \text  {dia}^*/2\rceil -3, \text  {dia}^* - 6 -\delta _3]$. Each vector $\pmb {w}\in \text  {W}_{\text  {main}}^{(\delta _1+1)}({\mathtt{a}}, d, m)$ can be obtained from a combination of vectors $\pmb {w}^1\in \text  {W}_{\text  {end}+2}^{(\delta _1+1)}({\mathtt{a}}, d-1, m'')$ and $\pmb {w}^2\in \text  {W}_{\text  {end}}^{(\delta _2)}({\mathtt{a}}', d', m')$ such that $\delta _1 + \delta _2 = \text  {dia}^* - 4$ and $\delta _1 \ge \delta _2$, as illustrated in Fig. 17. For each vector $\pmb {w}\in \text  {W}_{\text  {main}}^{(\delta _1+1)}({\mathtt{a}}, d, m)$, we store a sample tree $T_{\pmb {w}}$. This step can be designed in a similar way as Step 3 for the case of ${\text  {bl}}_2(G)=2$.

#### Step 5: Enumeration of feasible vector pairs

Analogously with the case of ${\text  {bl}}_2(G)=2$, a *feasible pair* of vectors is defined to be a pair of vectors $\pmb {w}^1= (\pmb {w}^1_{{\text  {in}}}, \pmb {w}^1_{\text  {ex}}) \in \text  {W}_{\text  {main}}^{(\delta _1+1)}({\mathtt{a}}_1,d_1, m_1)$, and $\pmb {w}^2= (\pmb {w}^2_{{\text  {in}}}, \pmb {w}^2_{\text  {ex}}) \in \text  {W}_{\text  {end}}^{(\delta _3)}({\mathtt{a}}_2,d_2, m_2)$, $\delta _1 \in [\lceil \text  {dia}^*/2\rceil -3, \text  {dia}^* - 6 -\delta _3]$, ${\mathtt{a}}_i\in \Lambda$, $d_i\in [1,d_\text  {max}-1]$, $m_i\in [d_i,{\text  {val}}({\mathtt{a}}_i)-1]$, $i = 1,2$ that admits an adjacency-configuration $\gamma = ({\mathtt{a}}_1, {\mathtt{a}}_2, m) \in \Gamma$ and a bond-configuration $\mu = (d_1 + 1, d_2 + 1, m) \in \text  {Bc}$ with an integer $m \in [1, \min \{3, {\text  {val}}({\mathtt{a}}_1) - m_1, {\text  {val}}({\mathtt{a}}_2) - m_2\} ]$ such that

- $$\pmb {x}^*_{{\text  {in}}} = \pmb {w}^1_{{\text  {in}}} + \pmb {w}^2_{{\text  {in}}} + \pmb {1}_{\gamma } + \pmb {1}_{\mu }$$ and $$\pmb {x}^*_{\text  {ex}} = \pmb {w}^1_{\text  {ex}} + \pmb {w}^2_{\text  {ex}}$$.

Step 5 computes the set of all feasible vector pairs $(\pmb {w}^1,\pmb {w}^2)$ by using a sorting algorithm as in the Step 4 for the case of ${\text  {bl}}_2(G)=2$.

#### Step 6: Construction of chemical graphs

Analogously with Step 4 for the case of ${\text  {bl}}_2(G)=2$, Step 6 constructs a target graph $T_{(\pmb {w}_1, \pmb {w}_2)}\in {\mathcal {G}}(\pmb {x}^*)$ for each feasible vector pair $(\pmb {w}^1,\pmb {w}^2)$ by combining the sample trees $T_i=T_{\pmb {w}^i}$ of vectors $\pmb {w}^i$ with a new edge $e=r_1(T_1)r_1(T_2)$.

## Abbreviations

ANN: Artificial neural network
MILP: Mixed integer linear programming
